# Research Progress of Polyacrylamide Gels for Oilfield Applications: A Systematic Review

**DOI:** 10.3390/gels12100903

**Published:** 2026-10-07

**Authors:** Ruiqiang Dong, Guanyu Chen, Daoyong Li, Xinyue Cui, Qianbing Lin, Xingyao Gao, Shiyu Zhang, Lianghui Guo, Fei He

**Affiliations:** 1 Sinopec Key Laboratory of Enhanced Oil Recovery for Fractured-Vuggy Reservoirs, Urumqi 830011, China; 2 School of Petroleum Engineering, China University of Petroleum (Beijing) at Karamay, Karamay 834000, China; 3 School of Chemical Engineering and Environment, China University of Petroleum (Beijing) at Karamay, Karamay 834000, China; 4 Xinjiang Key Laboratory of Multi-Medium Pipeline Safety Transportation, Karamay 834000, China; 5 Consulting Center of Xinjiang Oilfield Company, Karamay 834000, China

**Keywords:** polyacrylamide gel, profile control, water shut-off, cross-linking system, nano-composite modification, artificial intelligence

## Abstract

In the middle-to-late stage of reservoir development, widespread high-permeability channeling exacerbates ineffective water circulation and impairs crude oil recovery. This work explores polyacrylamide (PAM) gels intended for oilfield profile control and water shut-off. It comprehensively compares gelation behaviors and performance metrics of diverse cross-linking and modification strategies, and pinpoints critical unresolved research gaps. Four representative PAM-gel systems are analyzed in detail, including organic-crosslinked, metal-crosslinked, nano-composite-modified, and delayed-crosslinker formulations. The results show that polyethyleneimine (PEI) cross-linked gels achieve a storage modulus of 780 kPa at 110 °C, which is superior to the conventional hydroquinone-hexamethylenetetramine (HQ-HMTA) system. Metal-crosslinked gels are prone to syneresis and structural instability above 70 °C. Nanocomposite modification significantly enhances gel comprehensive properties: nano-silica improves thermal resistance to 155.5 °C; modified nano-graphite doubles storage modulus (G′), shortens gelation time by half, and maintains 76.5% of initial modulus after 60-day aging at 130 °C; TOCN-reinforced gels preserve structural integrity under high-salinity environments at 140 °C for 108 h. Delayed-release cross-linkers can extend gelation time up to 17 days to meet the demands of deep in situ profile control. Furthermore, the emerging data-driven paradigm of artificial-intelligence-assisted gel optimization is also reviewed, together with its current limitations. Future research priorities cover intelligent responsive gel materials, environmentally benign cross-linkers, and unified evaluation specifications for heterogeneous reservoirs. This paper reviews over 100 core publications from 2000 to 2026 based on the Web of Science and Engineering Village databases. Different from existing studies, this work emphasizes the molecular crosslinker mechanisms, nanomodification reinforcement, and field performance variations of PAM gel systems, and concludes the major technical bottlenecks for their application in harsh reservoirs. Meanwhile, it quantitatively compares the performance of modified PAM gel formulations and clarifies the applicable temperature ranges of different crosslinker schemes, providing technical references for the development of PAM plugging agents for complex oil reservoirs.

## 1. Introduction

### 1.1. Research Background and Practical Engineering Challenges

China holds abundant unconventional oil and gas resources. Against the backdrop of growing global energy demand, efficient exploitation of these resources has become a core strategic priority for the domestic petroleum industry. Nevertheless, complex geological characteristics, including low porosity-permeability, substantial in situ stress differences, and strong reservoir heterogeneity, impose major technical barriers to hydrocarbon recovery [1].

During the middle-to-late stage of oilfield production, water flooding is widely implemented to enhance oil recovery [2]. Reservoir heterogeneity coupled with long-term water injection readily generates high-permeability channels and fractures that form preferential water-flow pathways. Injected water tends to propagate along these low-resistance zones, triggering ineffective water circulation and severe water channeling [3]. This phenomenon manifests as rapid water-cut escalation, diminished oil output, and large-scale residual oil entrapment underground, which brings considerable energy waste and increases production costs [4].

Conventional mechanical plugging and cement-slurry water-shutoff technologies can mitigate channeling to a limited extent, yet they suffer from prominent practical drawbacks: complicated field operations, high costs, strict well-structure requirements, and poor formation adaptability [5,6]. Under such circumstances, chemical plugging technologies exhibit promising application prospects, among which polyacrylamide (PAM) gel systems are the most widely deployed [7].

PAM gels are generally formulated from partially hydrolyzed polyacrylamide (HPAM) and crosslinkers. The initial sol features low viscosity and can readily penetrate formation pores and fractures. Subjected to reservoir temperature, in situ crosslinker proceeds gradually, converting the sol into a three-dimensional network gel with favorable viscoelasticity [8]. The resulting gel selectively occludes high-permeability water-channeling zones, diverts subsequent injected water toward unswept low-permeability zones, and ultimately improves sweep efficiency and crude oil recovery [9].

HPAM benefits from accessible raw materials, facile synthesis, low cost, mature large-scale manufacturing, and excellent thickening capacity, rendering it a dominant polymer for water-flooding-based enhanced oil recovery (EOR) [10]. Its high molecular weight and intra-chain electrostatic repulsion further strengthen thickening performance, while crosslinker reactions build gel networks with considerable mechanical strength [11]. Owing to low viscosity and low injection pressure, PAM gels can penetrate deep into reservoirs and achieve selective fracture plugging. Combined with a low resistance factor and high residual resistance factor, they realize deep mobility control [12]. Furthermore, gelation time and gel strength can be flexibly tuned by adjusting polymer concentration, crosslinker type, and temperature to accommodate diverse reservoir conditions. Notably, PAM-derived materials serve multiple oilfield purposes such as fracturing-fluid thickeners and profile-modulation flooding agents. This review focuses exclusively on PAM gels for reservoir water shut-off and profile control; other application scenarios will not be discussed in detail [13,14].

### 1.2. Existing Bottlenecks and Current Research Progress

Despite widespread field application, practical deployment of PAM gel systems still faces multiple technical constraints. Gelation behavior is governed synergistically by injection parameters, formation temperature, and salinity, which creates challenges for precise regulation of crosslinker kinetics and gel quality (see Section 2.1.2 for detailed gelation mechanisms) [15]. Most in situ-formed gels rely on underground reactions between polymers and crosslinkers; high-concentration polymer solutions suffer severe shear degradation during injection, which degrades the final plugging performance [16]. In addition, PAM gels exhibit insufficient long-term stability and are vulnerable to formation-fluid erosion and chemical degradation [17]. For highly heterogeneous fractured reservoirs with intricate pore-throat architectures, uneven gel distribution frequently occurs and further impairs plugging efficiency [18]. Safety and environmental concerns also remain unresolved. Gel systems are often used together with oxidizing gel breakers (e.g., ammonium persulfate), which may induce corrosion of downhole pipelines and facilities [19]. Moreover, particle-agent plugging mechanisms during hydraulic fracturing are still poorly understood, and field operations largely rely on empirical experience [20].

Multiple material-oriented optimization strategies have been proposed to address the above limitations. Functional-monomer grafting enables molecular modification of PAM matrices. For instance, tannic-acid-modified pre-crosslinked particle gels construct dual-network hydrogels that retain stable swelling under 120 °C high-salinity conditions, achieving plugging efficiency above 90% [21]. Nano-composite gels and smart-responsive gels represent alternative routes to improve reservoir adaptability. Prior research demonstrated that composite hydrogels consisting of PAM, scleroglucan, and oxidized cellulose nanofibers can maintain structural integrity for 108 h under 140 °C saline environments [7]. Formulation-driven optimization mainly centers on delayed-release crosslinkers for controllable gelation; sodium styrene sulfonate (SSS) functions as a polymerization retarder to effectively prolong gelation periods [22].

Recent advances in EOR technologies have further enriched the optimization pathways of polymer-based oil displacement and plugging systems. A large number of emerging strategies, including nano-reinforced polymers, hybrid injection techniques, capsule polymer flooding, and machine learning-based performance prediction, have been systematically summarized in recent reviews, revealing achievable recovery ranges, wettability alteration effects, and key controlling factors for nano-modified polymer systems [23]. Although these studies mainly focus on non-gelling polymer flooding rather than in situ cross-linked gel plugging, they highlight universal technical bottlenecks including nanoparticle agglomeration, formation retention, high synthesis cost, and insufficient long-term field scalability, which are also critical challenges restricting the further development of high-performance PAM plugging gels.

Research on PAM-gel-based water shut-off and profile-control technologies has advanced rapidly in recent years. Existing reviews have summarized general EOR technical portfolios and clarified functional design principles of polymer-microsphere composite systems for deep profile modification [5,24]. Nevertheless, existing reviews rarely comprehensively integrate molecular design, nano-composite modification, and process regulation to jointly resolve compound challenges of PAM gels under high-temperature, high-salinity, and strongly heterogeneous reservoir settings, including poorly controlled gelation, inferior long-term stability, and insufficient deep plugging capacity.

### 1.3. Research Methodology of This Review

Accordingly, this review focuses on modification strategies and synergistic performance optimization of PAM gels adapted to complex reservoir environments. To ensure comprehensiveness and pertinence, a two-tiered literature collection strategy was adopted in this work.

First, a macroscopic bibliometric analysis was performed based on 347,341 PAM gel-related publications retrieved from the Web of Science (WOS) database, aiming to clarify the global research panorama and developmental trends in this field.

Second, targeted literature screening was conducted across the WOS Core Collection and Engineering Village databases. The retrieval period covered 2000–2026, and all searches were completed on 10 July 2026. Core keywords regarding PAM materials, crosslinkers, nanocomposite modification, and oilfield EOR applications were combined with Boolean operators for accurate retrieval.

Subsequently, duplicate records were eliminated, and the remaining publications were screened stepwise by title, abstract, and full text. Strict inclusion and exclusion criteria were implemented: only peer-reviewed English journal articles with original experimental data concerning oilfield profile control and water shutoff PAM gels were retained, whereas reviews, conference abstracts, patents, and non-oilfield studies were excluded.

After rigorous screening, 105 highly relevant core papers were systematically analyzed. On this basis, this review summarizes the mainstream technical routes for developing high-performance and long-lifespan PAM plugging gels, including polymer molecular structure optimization, crosslinker screening, nanomaterial synergistic modification, and injection parameter regulation for harsh reservoir conditions.

### 1.4. Bibliometric Overview of Global PAM-Gel Research

Bibliometric statistics reveal clear temporal and geographical patterns within global PAM-gel research. The developmental trajectory can be divided into three distinct phases. The pre-2007 initial phase produced 31 196 papers (9.0% of the total), during which European and North American research groups laid the fundamental theoretical framework for PAM gels. The 2007–2016 rapid-growth phase yielded 102 202 papers (29.4% of the total); rising publication outputs from Asia promoted a multi-polarized global research landscape. The 2017–2026 explosive-development phase contributed 213 943 papers (61.6% of the total), and Asia emerged as the leading continental contributor. Cumulative publication metrics rank Asia in first place with 129 148 records, followed by Europe (109 272 records) and North America (76 775 records). South America, Oceania and Africa remain comparatively minor research regions. Within Asia, China dominates the research output, generating 61 360 publications merely over the 2017–2026 period. Divergent research priorities arise from regional industrial demands. Asian researchers (predominantly from China, Iran, Saudi Arabia, and Russia) focus on oil-field-oriented high-temperature-salinity-resistant PAM gels for profile control and water shut-off, where nano-composite modification stands out as a prevailing research hotspot [25]. European and North American teams carry out diversified investigations covering oilfield applications, water treatment, biomedical hydrogels, and fundamental mechanism exploration. Japanese and Korean scholars emphasize molecular-level design of novel functional gels, whereas studies from South American and African institutions mainly respond to local demands for petroleum exploitation and wastewater treatment [25]. The statistical results derived from the above bibliometric retrieval are plotted in Figure 1.

These bibliometric outcomes outline the macroscopic developmental landscape of PAM-gel research. To further unpack the underlying material-scale mechanisms, subsequent sections systematically elaborate gelation mechanisms, cross-linking-system characteristics, and nano-composite modification strategies for oilfield-applied PAM gels. These bibliometric outcomes outline the macroscopic developmental landscape of PAM-gel research. To further unpack the underlying material-scale mechanisms, subsequent sections detail gelation mechanisms, cross-linking-system characteristics, and nano-composite modification strategies for oilfield-applied PAM gels.

The remainder of this review is structured as follows. Section 2 systematically elaborates three gelation mechanisms (covalent cross-linking, ionic coordination cross-linking and physical cross-linking), The remainder of this review is structured as follows. Section 2 details three gelation mechanisms (covalent cross-linking, ionic coordination cross-linking and physical cross-linking), and compares compatibility, gelation kinetics and plugging performance among organic cross-linkers, metal-ion cross-linkers and special functional cross-linkers. Section 3 summarizes recent advances in nano-composite-modified PAM gels, focusing on reinforcement mechanisms and performance improvements of nano-silicon dioxide (nano-SiO_2_), graphene-derived materials, TEMPO-oxidized cellulose nanofibers (TOCN) and tetrapod zinc-oxide whiskers (T-ZnOw), with particular emphasis on temperature-salinity tolerance, mechanical properties, long-term stability and plugging efficiency under harsh reservoir conditions. Section 4 draws the principal conclusions of this review. Section 5 puts forward prospects for future research, including intelligent responsive gel materials, environmentally benign cross-linkers, and standardized evaluation specifications for heterogeneous reservoirs.

## 2. PAM Gel System

During the late stage of oilfield development, due to the presence of fractures or high-permeability formations, injected water tends to channel along high-permeability pathways, compromising displacement efficiency [26]. Studies have demonstrated that gel plugging technology has emerged as an effective method for enhancing oil recovery, significantly reducing operational costs while further improving oil recovery rates. Among these, polyacrylamide gel technology is widely applied due to its advantages, including excellent injectability, highly selective plugging, controllable gelation time, and robust plugging strength [27].

### 2.1. Gelation Mechanism of Polyacrylamide Gel

#### 2.1.1. PAM

PAM is a water-soluble polymeric compound with a linear structure, formed through homo-polymerization of acrylamide (AM) monomers or copolymerization with other monomers [28]. The chemical structures of PAM and HPAM are illustrated in Figure 2 [9].

PAM exhibits a rigid, glassy polymer state at room temperature, being colorless, non-toxic, and non-corrosive. It is soluble in water but insoluble in ethanol. Additionally, PAM demonstrates excellent thermal stability and hydrophilicity. Based on the ionization characteristics of the functional groups on the PAM macromolecular chains, PAM can be classified into four categories: non-ionic polyacrylamide (NPAM), cationic polyacrylamide (CPAM), anionic polyacrylamide (APAM), and amphoteric polyacrylamide (AmPAM).

#### 2.1.2. Gelation Mechanism of Polyacrylamide

The gelation of polyacrylamide involves using crosslinkers to bridge linear PAM chains into a three-dimensional network structure, which encapsulates water molecules to form a gel. Based on the gelation mechanism, it can be classified into covalent crosslinkers, coordinate crosslinkers, and physical crosslinkers [9].

1. Covalent Crosslinker Copolymerization

Covalent crosslinker copolymerization involves utilizing the amide groups (-CONH_2_) on the molecular chain as a reactive site, which undergoes nucleophilic substitution or condensation reactions with active groups (such as amino groups, aldehyde groups, etc.) in crosslinker molecules under suitable conditions, forming stable covalent bridge bonds (e.g., -CH_2_-NR-, -CO-NR-, etc.). These covalent bonds form at multiple points between PAM chains and expand into a three-dimensional network, thereby transforming the linear polymer into a crosslinked gel with a spatially stable structure [29]. Taking the reaction between PAM and PEI under suitable conditions as an example, as shown in Figure 3, the imine nitrogen atoms in the PEI molecule act as nucleophilic centers and attack the carbonyl carbon atoms in the -CONH_2_ on the PAM chains, undergoing nucleophilic substitution reactions. Through intermolecular dehydration, stable amide-type covalent bridge bonds (-CO-NR-) are formed. These covalent bonds directly connect the PAM and PEI molecular chains, constructing a three-dimensional network structure through multi-point covalent crosslinking, thereby forming a gel system with spatial stability.

2. Ionic Coordination Crosslinker

For partially HPAM, which contains carboxyl groups (-COO−), gelation can occur through ionic coordination with polyvalent metal ions (such as Cr^3+^, Al^3+^, Zr^4+^). The high-valence metal central ions coordinate and complex with the negatively charged carboxyl groups (-COO−) on the HPAM molecular chains, replacing the originally coordinated water molecules and forming stable coordination bonds [30]. A single high-valence metal central ion can simultaneously bind to carboxyl groups on two or more different HPAM chains, ultimately forming an “ionic bridge” network with metal ion clusters as crosslinker nodes. This network encapsulates water molecules to form a gel system [31]. Prior to crosslinking with the polymer, Cr3+ first forms polynuclear hydroxy-bridged complex ions through complexation, hydrolysis, and polynuclear hydroxy-bridging interactions. The ionic coordination complexation reaction between hydrated chromium ions ([Cr(H_2_O)_6_]^3+^) and HPAM is illustrated in Figure 4. Using trivalent chromium ions (Cr^3+^) as the crosslinker center, their vacant orbitals can accept lone pair electrons from the oxygen atoms in the -COO− groups on the partially HPAM molecular chains, forming coordination bonds. A single Cr^3+^ ion can simultaneously coordinate with multiple carboxyl groups on two or more HPAM chains, thereby connecting discrete linear polymers into an integrated three-dimensional network structure and forming a gel with spatial stability and viscoelasticity.

3. Physical Crosslinker

Physical-crosslinker hydrogels are formed through non-covalent interactions such as hydrogen bonding, hydrophobic association, and electrostatic attraction [32]. Hydrogen bonds strengthen intermolecular associations, regulate mechanical properties, and confer thermos-responsive behavior to gel networks. Unlike stable covalent cross-links, these non-covalent interactions are reversible and sensitive to temperature, pH, and salinity, leading to strong environmental dependence. For oilfield PAM gels, physical cross-linking seldom serves as the primary network-building mechanism; nanofillers and functional additives are mostly applied as auxiliary reinforcers to increase non-covalent sites and cross-linking density upon covalent or ionic-coordination main networks.

Reported reinforcing performances of nanofillers are strongly affected by preparation routes, which leads to inconsistent experimental results. Some typical gelation mechanisms of PAM gels and their key characteristics are shown in Table 1, Qin et al. [33] obtained strengthened GO/PAM composite gels via in situ preparation driven by hydrogen-bond interactions. However, direct physical mixing of GO and PAM causes competitive hydrogen-bond occupation, damages the cross-linking network, and reduces gel strength, demonstrating that the fabrication method determines whether GO brings reinforcement or deterioration. Qiang Li et al. [25] incorporated CTAB-modified C-SiO_2_ into PAM-PEI systems to improve gel stability via ionic and hydrogen-bond effects. Paulo César França da Câmara et al. [34] further found that crude glycerol (CG) can form combined ionic-hydrogen-bond interactions to compact PAM-PEI hydrogel networks.

Notwithstanding these promising laboratory outcomes, additive-mediated physical reinforcement faces practical constraints for HTHS reservoirs. Non-covalent interactions readily decay under high-temperature, high-salinity, and extreme-pH environments. Accordingly, nanofiller-enabled physical enhancement functions only as a complementary strategy rather than a standalone gel-forming approach for oilfield profile control and water shut-off.

**Table 1:** Typical gelation mechanisms of PAM gels and their key characteristics.

Gelation Mechanism	Type of Action	Reaction Site	Characteristic	Typical System
Covalent cross-linked copolymerization	Formation of covalent bridging bonds via nucleophilic substitution or condensation reactions	PAM molecular chain amide group (–CONH_2_)	Covalent bonds exhibit high chemical stability and a robust network structure.	PAM-PEI System
Ion coordination crosslinker	Ion coordination complexation: formation of an ion bridge network	HPAM molecular chain carboxyl group (–COO^−^)	The coordination bond is strong, giving the gel good viscoelastic properties; the gelation behavior is readily influenced by temperature.	HPAM–Cr^3+^ system
Ion coordination crosslinker	Non-covalent interactions such as hydrogen bonding, hydrophobic association, and electrostatic attraction	Polymer chain polar groups and hydrophobic segments	It is reversible, sensitive to temperature, pH, and mineralization degree, and highly dependent on the environmental conditions; it serves as a supplementary enhancer for the system.	PAM/Graphene Oxide Composite System

### 2.2. The Effect of Crosslinker Type on Gels

As a key component of polyacrylamide gel systems, crosslinkers drive linear polymer chains to construct three-dimensional network architectures through chemical bonding or non-covalent physical interactions with PAM molecular chains. They further determine gel strength, long-term stability, and adaptability to reservoir environments [35]. Since physical interactions mainly serve as auxiliary reinforcement for gel networks, practical oil-field plugging gels largely depend on crosslinker-triggered covalent or ionic-coordination reactions. Based on oilfield application scenarios and published research data, mainstream crosslinkers for polyacrylamide gels can be classified by their chemical properties and gel-formation mechanisms. Different crosslinker categories show substantial differences in reaction kinetics, applicable reservoir conditions, and final gel performance. Widely investigated and field-deployed crosslinker families for oilfield use include organic crosslinkers [36,37,38,39,40], metal-ion-based crosslinkers, and specially functionalized crosslinkers.

#### 2.2.1. Organic Crosslinker

Organic cross-linking reactions generate robust covalent linkages between amide moieties of polymer chains and cross-linker molecules, bridging individual polymer strands to build thermally resilient three-dimensional network architectures [41,42]. Of the available organic cross-linkers, phenol-formaldehyde (PF)-based and PEI-crosslinked gel systems have garnered substantial interest owing to their capacity to yield stable gels under high-temperature reservoir environments. PEI stands out as one of the most extensively investigated organic cross-linking agents: it conforms to green chemistry requirements as an environmentally benign reagent, displays broad compatibility with diverse polymers, and possesses plentiful nucleophilic sites along its backbone that facilitate efficient cross-linking with polymer amide groups [39]. As schematically depicted in Figure 5, the -CONH_2_ groups on PAM chains undergo nucleophilic substitution with amine moieties (-NH_2_, -NHR) from PEI. This reaction releases ammonia (NH_3_) as a by-product and forms new amide bonds (-C-NH-), covalently connecting PAM and PEI molecular chains. Progressive cross-linking among linear polymer segments ultimately assembles the three-dimensional gel network [43].

The interaction mechanism between the amine groups of PEI and the amide/carboxyl groups of partially hydrolyzed polyacrylamide was investigated in depth using nuclear magnetic resonance (NMR) techniques. From the diffusion-ordered spectroscopy (DOSY) spectra shown in Figure 6, the PAM/PEI1300 mixture was shown to exhibit one diffusion coefficient of 5 × 10^−12^ m^2^s^−1^ (in black), which was lower than those of PAM and PEI alone (blue and red), indicating the presence and formation of a higher molecular weight component. The spectra showed the formation of higher molecular weight components when PAM and PEI were mixed for a sufficient time at room temperature. Ghriga et al. [44] found that the NOESY spectra confirmed that the spatial proximity between the protons of the amide groups of the polymer and the amine groups of the crosslinker is independent of the temperature, revealing the strong nature of the interaction between PAM and PEI.

To further enhance the stability of gels and broaden their applicability in high-temperature and high-pressure environments, Wang et al. [40] designed and synthesized Fe_3_O_4_@PEI using a one-pot method to produce ferrofluid, which was then cross-linked with PAM to prepare magnetic smart polymer gels. In the absence of Fe_3_O_4_@PEI, PAM molecules in the solution primarily exist in a disordered helical structure. Upon the introduction of Fe_3_O_4_@PEI, PAM molecules undergo an amidation reaction withFe_3_O_4_@PEI, forming a honeycomb-like three-dimensional network structure that enhances the gel’s shear resistance, thereby effectively blocking fluid flow in highly permeable formations, as shown in Figure 7.

When the Fe_3_O_4_@PEI concentration was increased from 2 wt% to 8 wt%, the initial viscosity of seamless polymer gel-X (SPG-X) increased from 130 mPas to 161 mPas, giving SPG-X good injectability and making it suitable for on-site application in oilfields. As the concentration of Fe_3_O_4_@PEI added to SPG-X increased from 2 wt% to 8 wt%, the gel strength of SPG-X rose from 38,990 mPas to 137,165 mPas on the fifth day, as shown in Figure 8. This is because Fe_3_O_4_@PEI increases the crosslinker density of SPG-X, forming a denser three-dimensional network structure, thereby enhancing gel strength. Although gel strength decreased slightly after the gelation period exceeded 5 days, it remained at a high level even after 30 days. This indicates that the addition of Fe_3_O_4_@PEI confers excellent long-term stability to the SPG.

Although PEI offers excellent performance, its high cost limits its widespread application in oilfield extraction. Phenol-formaldehyde crosslinkers are inexpensive, but due to the toxicity of phenol and the carcinogenic risks associated with formaldehyde, they pose serious environmental and safety hazards [36]. To address this issue, researchers have sought less toxic alternatives to phenol/formaldehyde. Zhang et al. [37] sought low-toxicity alternatives to phenol/formaldehyde, synthesized the low-toxicity, water-soluble hydroxymethyl polyphenol Water-soluble hydroxymethyl polyphenols (THMBPA), and then used THMBPA as the main crosslinker and polyethylene polyamine TET as the auxiliary crosslinker. They investigated the effects of pH, crosslinker ratio, temperature, and salt mass fraction on the gelation time of the gel system, and examined the long-term stability of this gel system at different temperatures. Li et al. [45] isolated cellulose nanofibers through 2,2,6,6-tetramethylpiperidine-1-oxyl radical oxidation (TEMPO-oxidation) pre-treatment followed by mechanical disintegration. Then, a series of shape memory TOCN/PAM/Gelatin hydrogels with varying TOCNs and gelatin contents were prepared through a facile in situ free-radical polymerization method. After the polymerization, TOCNs, gelatin, and PAM were distributed homogeneously in the hydrogels. Such a composite hydrogel with good shape memory behavior and enhanced mechanical strength would be an attractive candidate for a wide variety of applications. Liu et al. [38] found through their research that common alternatives include HMTA and hydroquinone (HQ). Their gelation mechanisms are shown in the figure below; Figure 9a shows that HMTA decomposes under acidic conditions or at high temperatures to produce formaldehyde and NH_3_. Figure 10 shows that formaldehyde undergoes a nucleophilic substitution reaction with HQ to form 2,5-dihydroxymethylquinone. In Figure 9c, 2,5-dihydroxymethylquinone molecules condense to form a phenolic prepolymer, which acts as a crosslinker working with the polymer. Figure 9d: Under heating or acidic conditions, the hydroxymethyl groups (-CH_2_OH) on the phenolic prepolymer undergo a condensation reaction with the -CONH_2_ on the side chains of the polymer (PAM), releasing one molecule of water to form a stable nitrogen-methylene bridge (-CH_2_-NH-). Multiple reaction sites on the phenolic prepolymer link multiple PAM chains, thereby forming a stable, uniformly distributed three-dimensional network gel structure. Lu et al. [46] have presented a development of modified nano-graphite (MG) as a crosslinker to enhance the temperature and salt resistance of gel systems and enable their use in deep oil and gas reservoirs. The novel nano-graphite hybrid crosslinked gel was formed by combining PAM as the polymer matrix with HMTA, HQ, and MG as the crosslinkers.

Cui et al. [29] compared PAM cross-linked with PEI (PAM-PEI) and PAM cross-linked with HQ-HMTA (PAM-HQ-HMTA) and found that the gelation times for the PAM-PEI and PAM-HQ-HMTA gels were reduced from 120 h and 7 h to 6 h and 1 h, respectively. Therefore, the gel crosslinker time for HQ-HMTA was longer than that for the PEI crosslinker, and the strength of all gel samples reached Code I after the crosslinking process was completed. Ma et al. [7] developed a mechanically stable and high-temperature-resistant composite hydrogel (STP) by incorporating biodegradable scleroglucan (Slg) and TOCN in the PAM hydrogel.

Figure 10 shows the rheological behavior of the gel samples at 110 °C. The PAM-PEI gel has a maximum G′ of 780 kPa, which is higher than that of the PAM-HQ-HMTA gel [47]. This modulus disparity primarily stems from fundamental differences in crosslinker mechanisms and network architectures. For PEI-based systems, the crosslinker occurs via a trans-amidification reaction between the primary amine groups of PEI and the amide groups of PAM, forming stable covalent amide bonds that create a dense, highly interconnected three-dimensional network [44]. This reaction pathway is direct and efficient, with the branched structure of PEI providing multiple nucleophilic sites per molecule, enabling simultaneous crosslinking at numerous points along the PAM chains [48]. In contrast, the HQ-HMTA system operates through a multi-stage, stepwise mechanism: HMTA first thermally decomposes to generate formaldehyde, which then undergoes nucleophilic substitution with HQ to form hydroxymethyl derivatives; these intermediates further condense into phenolic prepolymers before finally reacting with PAM to establish crosslinks [49]. This cascade of sequential reactions inherently limits the achievable crosslink density, as the effective concentration of reactive intermediates at polymer sites is constrained by diffusion and reaction kinetics. Consequently, PEI-crosslinked gels exhibit superior viscoelastic properties, with studies reporting storage moduli exceeding 2689 Pa under comparable conditions [5]. Furthermore, the high-density amine functionality of PEI allows for additional stabilization mechanisms—such as hydrogen bonding and electrostatic interactions—that synergistically reinforce the gel network beyond covalent crosslinker alone [32]. In contrast, gels crosslinked solely with phenolic systems show more limited elastic recovery, although incorporating both PEI and phenolic resin in a triple-synergy formulation can partially bridge this performance gap by establishing a multimodal crosslinker network [50]. Therefore, the higher G′ of the PAM-PEI gel is attributed to its combination of direct amide-bond formation, high branching density of the crosslinker, and auxiliary non-covalent interactions that collectively yield a tighter, more resilient three-dimensional network at elevated temperatures.

#### 2.2.2. Metal Crosslinker

Metal crosslinkers bind to polyacrylamide molecules through ionic bonds. Common multivalent metal ions such as Cr^3+^, Zr^4+^, and Al^3+^ are used. The chemical bonding force of the metal crosslinker system is strong, providing better temperature resistance [51]. However, compared to organic crosslinked gels, metal crosslinked gels usually exhibit lower thermal stability and poorer shear resistance [52]. In addition, at high temperatures (>70 °C), the reaction rate between metal crosslinkers and polymers is too fast, making it impossible to place the gel system in the target area, limiting its application in high-temperature reservoirs.

Figure 11 illustrates the rheological property changes of the Cr^3+^ gel system containing 1% polyacrylamide and 0.2% chromic acetate at 140 °C over a period of 0 to 15 days [53]. The results indicate that the absolute viscosity of the system gradually decreases with time, while the G′ and G″ remain essentially stable. This is due to the gradual hydrolysis of the amide groups on the side chains of polyacrylamide to carboxyl groups under high-temperature conditions, leading to excessive crosslinking between the C-O bonds of -COOH and Cr^3+^. This exacerbates the synergistic effect of the system, which is also a reason for gel dehydration and shrinkage [53]. Although the gel maintains a high strength, its water control capability has significantly decreased, making it difficult to effectively perform fluid diversion functions.

Carboxyl groups (-COOH) on polyacrylamide form coordinate bonds with Cr^3+^. Zhang et al. [53] reported that crosslinker reactions enable polyacrylamide to construct a three-dimensional network structure and further produce a gel. The three-dimensional network binds water into the gel matrix, maintaining moderate mechanical strength even under high-temperature conditions, as illustrated in Figure 12.

Figure 13 shows the influence of retarder (EDTA-4Na) concentration on gelation rate. In the base gel (40,000 mg/L HPAM, 4000 mg/L phenolic resin, 2 wt% nanobentonite, 1:10 Cr^3+^/HPAM), crosslinking began within 2 min at 20 °C, marked by a sharp viscosity increase. Adding 0.5 wt% retarder (S1) introduced a pre-gelation phase (32 min) with shear-thinning behavior, where viscosity dropped from 97.4 to 57.8 mPa·s, before a sudden rise. Crosslinker time and temperature increased to 32 min and 69.3 °C. With 1 wt% (S2), time and temperature rose to 36 min and 70.9 °C; at 2 wt% (S3), to 41 min and 75.4 °C; and at 3 wt% (S4), to 46 min and 77.8 °C. However, further increases to 4 wt% (S5) and 5 wt% (S6) did not further extend crosslinker time. Although the gel maintains a high strength, its water control capability has significantly decreased, making it difficult to effectively perform fluid diversion functions.

When chromium acetate is used as a crosslinker, polyacrylamide can form a dense dendritic three-dimensional network structure (Figure 14a). These dendritic structures provide the gel with strong skeletal support, endowing it with high mechanical strength. However, they also lead to an increased crosslinker density within the network, causing the gel to shrink after high-temperature treatment. A current alternative to Cr^3+^-phenolic resins is the use of lysine crosslinker [56], as depicted in Figure 14a; the bulk gel has a dense network structure. The crosslinked gel’s swelling and deswelling/syneresis behavior significantly impacts the gel strength and plugging efficiency. The crosslinked gel, as a polyelectrolyte, could absorb water from the surrounding rock and keep swelling till reaching the equilibrium swelling ratio, or completely fill the pores or tiny fractures before reaching the equilibrium swelling ratio. The gels could act as a pore-filling material to fully plug the high-permeability channels. Therefore, we also tested the swelling behavior of the bulk gel in different brines at 80 °C, as shown in Figure 15b.

#### 2.2.3. Special-Functionalized Crosslinker

In PAM-based profile-control and water-shut-off gels for oilfields, cross-linkers are critical for constructing the gel’s three-dimensional (3D) network, regulating gelation behavior, and determining mechanical strength, reservoir environmental resistance, and field-application performance. Conventional polyacrylamide-gel cross-linkers that have been widely studied and field-deployed are mainly divided into two categories: metal-ion cross-linkers and synthetic organic cross-linkers. Cross-linkers possessing unique chemical functions to achieve favorable thermal-salt stability, controllable gelation for deep-reservoir conditions, low formation damage, and environmental responsiveness are defined as special-functionalized cross-linkers.

Against the technical demands of water-shut-off and profile-modification for high-temperature, high-salinity deep/ultra-deep fractured low-permeability oil reservoirs, research on special-functionalized cross-linkers concentrates on three major branches: nano-composite cross-linking agents, environmentally-responsive cross-linking agents, and delayed-controlled-release cross-linking agents. Notably, these specially functionalized cross-linkers are generally developed as targeted optimization schemes to remedy the inherent drawbacks of conventional metal-ion and synthetic organic cross-linkers, rather than completely replacing the traditional cross-linking systems.

1. Nano-composite crosslinker

Nanomaterials can function as highly efficient nanofillers and composite crosslinker assistants to tailor polymer gels from both structural and performance perspectives [57]. Graphene oxide and other 2D nanomaterials strengthen gel networks and optimize gelation and thermal tolerance for ultra-deep high-temperature reservoirs [58]. Metal-oxide nanoparticles alleviate inherent limitations of metal-ion cross-linkers and improve gel performance under high-temperature, high-salinity reservoir conditions [59]. Compared with alternative fillers such as carbon nanotubes and nano-clay, nanographene and its oxide derivatives show advantages in production scalability, cost controllability, and environmental compatibility, making them promising candidates for fabricating high-performance water-blocking nano-composite gels. Rational tuning of nanoparticle parameters enables further optimization of key gel properties [60].

As illustrated in Figure 16, Qin et al. [33] fabricated polyacrylamide-graphene oxide (PAMGO) nano-composite gels via in situ radical polymerization of AM monomers with pre-dispersed GO nanosheets, using PEI as the cross-linker. The gel formation proceeds in two principal stages: establishment of the covalent primary network and subsequent reinforcement contributed by GO nanosheets. The covalent cross-linking between PAM and PEI follows the trans-amidation mechanism described in Section 2.2.1, which builds the fundamental polymer skeleton of the gel [58]. The in situ incorporated GO nanosheets bring multiple reinforcing effects: abundant oxygen-containing moieties (hydroxyl, epoxy, carboxyl groups) on GO surfaces form stable hydrogen-bond interactions with N- and O-containing sites on PAM chains. These create extra physical cross-linking sites beyond the covalent network and substantially raise the overall cross-linking density [33]. Furthermore, GO nanosheets occupy voids within the gel network, reduce internal porosity, and consolidate structural integrity [61].

By contrast, simple physical blending of PAM and GO produces an opposite effect. Hydrogen bonds formed between amide groups of PAM and oxygen-bearing groups on GO compete for potential PEI reaction sites. This suppresses covalent cross-linking density and ultimately causes severe deterioration in gel strength.

Figure 17 provides an intuitive comparison of the test results of three sets of core samples: The breakthrough pressure of pure PAM-L gel is 15.55 MPa; the in situ polymerized PAMGO-L nano-composite gel achieves a breakthrough pressure of 21.71 MPa, representing a significant increase of 20.71% compared to pure PAM gel; whereas the physically mixed PAM/GO gel used as a control exhibits a breakthrough pressure of only 3.72 MPa, indicating a decrease of 76.07% relative to pure PAM gel. However, simple physical mixing of GO severely reduces the crosslinker density and sealing capacity of the gel. This provides crucial experimental support for the application of this PAMGO composite gel in oilfield water shutoff and profile adjustment, as well as drilling leakage prevention [29].

Figure 18, as the core characterization diagram of gel rheological properties, focuses on the test results of G′ and G″, further verifying the core advantages of the composite gel from the perspective of intrinsic material properties [29]. This figure presents the rheological sweep curves of pure PAM gel and PAMGO composite gel, intuitively demonstrating the significant differences between the two in terms of structural strength and stability. Compared with traditional pure PAM gel, the in situ composite PAMGO gel exhibits prominent advantages. Throughout the entire tested frequency range, the G′ of the composite gel remains significantly higher than its loss G″, indicating a more stable and pronounced elastic solid characteristic. Its maximum G′ reaches 279.48 Pa, far exceeding that of pure PAM gel (195 Pa). This data directly demonstrates that the bulk strength, deformation resistance, and shear resistance of the composite gel have achieved a qualitative improvement. This advantage enables the gel to maintain an intact three-dimensional cross-linked network under long-term flushing and shear disturbances by formation fluids, avoiding structural damage and plugging failure. Fundamentally, this extends the on-site plugging validity period, compensating for the shortcomings of pure PAM gel such as loose structure, poor stability, and susceptibility to dispersion. It also completely avoids the defect of physical mixture gels where crosslinker sites are occupied, and performance is significantly degraded, making it the core intrinsic advantage of this nano-composite gel to meet the long-term operational requirements of oilfields.

2. Delayed-release crosslinker

Gelation time is a core parameter for the success of gel conformance control technology [62]. For crosslinked gels, all systems exhibited rapid gelation, which is suitable for near-wellbore treatments. However, for deep conformance control, if the gelation time is too short, the gel system is prone to premature crosslinking before reaching the target reservoir location [63]. Premature gelation not only hinders deep migration but also risks blocking mixing tanks or wellbores, leading to operational failure. Meanwhile, electrostatic adsorption and hydrogen bonding during transport cause significant loss of active components, undermining deep water shutoff. To address these two challenges, delayed gelation systems have become essential for deep conformance control. This not only prevents further migration into the deeper formation but may also cause blockages in mixing tanks or wellbores due to premature gel formation, directly leading to the failure of conformance control operations. Additionally, during the migration of the gel system through the formation, significant loss of active components occurs due to electrostatic adsorption and hydrogen bonding interactions, resulting in ineffective deepwater shutoff. To address these two industry pain points, delayed gelation systems are urgently needed for deep conformance control. Consequently, delay-retardant crosslinkers have emerged as a core category of specialized functional crosslinkers tailored to meet the demands of oilfield deep conformance control [64,65].

Dai, Wang et al. [66,67] conducted research and found that the delayed-release crosslinker is influenced by factors such as hydrolysis degree, the amount of PEI, salt concentration, and pH value. The hydrolysis degree influences the stretched state of the HPAM chains in the solution. In addition, a more stretched HPAM chain exposes more amide groups as potential crosslinker sites. However, it is important to note that a higher hydrolysis degree does not always result in improved gelation. A higher hydrolysis degree reduces the number of amide groups available for crosslinking [68]. Figure 19 depicts a trend that is consistent with the results obtained with the mixed brine. The gelation time of the HPAM/PEI system increased while the gel strength decreased with the increasing single-salt concentration. Lower-concentration systems were significantly less tolerant to salt at equivalent salinity. Table 2 shows that the most favorable conditions for the HPAM/PEI gel system were the neutral and alkaline conditions, with the best results obtained at pH~10.5. However, it failed to gel under highly alkaline conditions (pH~13).

Studies by Guo et al. [67,69] revealed that hydrogen-bond-mediated pre-association imparts delayed-gelation features to the HPAM/PEI system. Such behavior originates from a two-stage interaction mechanism between HPAM and PEI. At the initial stage, PEI mainly restricts HPAM chain conformation by forming hydrogen bonds with a small fraction of carboxyl groups (-COOH), which induces coiling of HPAM backbones and maintains low solution viscosity [70]. As the reaction evolves, covalent trans-amidation cross-linking (see Section 2.2.1) gradually takes over, where carboxylate groups along HPAM chains react with PEI to generate large-size aggregates within the system (Figure 20). This structural evolution accounts for the subsequent rise in viscosity and gel strength. As displayed in Figure 21, well-developed dense networks are obtained at both 30 °C and 100 °C, confirming effective gelation across this temperature interval. Although the network formed at 100 °C is slightly less compact than its 30 °C counterpart, it still reaches gel strength code G, demonstrating satisfactory thermal stability of the HPAM/PEI gel system.

Apart from the intrinsic two-stage kinetics of HPAM/PEI, gelation delay can also be realized by suppressing polymer reaction rates. Xu et al. [22] reported that adding low dosages of SSS (0.1–0.8 wt %) allows continuous modulation of gelation time in the range of 40–100 min relative to the SSS-free control.

Table 3 summarizes the performance mechanisms of various crosslinkers.

**Table 3:** Key properties of main cross-linker categories for oilfield PAM gels.

Crosslinkers	Typical System	Gelation Time, Gelling Time	Gel Strength	Long-Term Stability	Applicable Temperature
Organic crosslinker	PEI, HQ-HMTA, phenolic compounds	Duration adjustable from a few tens of minutes to several tens of hours	Reach Code I level; the PEI system exhibits an energy storage modulus of 780 kPa at 110 °C.	The modified PEI gel maintains high strength even after 30 days of aging.	High-temperature reservoirs (70–150 °C)
Metal ion crosslinker	Cr^3+^, Al^3+^, Zr^4+^	At 140 °C for 6–27 h	Achieves H-class strength	The modulus remains essentially stable within 15 days at 140 °C; however, when the temperature exceeds 70 °C, dehydration and water release are likely to occur.	Practical application for low-to-medium-temperature reservoirs below 70 °C; enables gel formation at 140 °C in laboratory conditions.
Nano-composite crosslinker	PAM-GO and CTAB-modified SiO_2_ composite system	PAM-PEI: 25–64 h; PAMGO-PEI: 4–14 h	Core breakthrough pressure: up to 21.71 MPa	Good stability at 30 °C	The verified temperature range is relatively narrow.
Sustained-release delayed crosslinker	HPAM-PEI sustained-release system; SSS polymerization inhibition system	Maximum duration adjustable up to 17 days (60 °C, mineralization level: 6000 mg/L)	Sydansk F-Class–High-Distortion Non-Flowing Gel	The system remains stable at 60 °C for up to 20 days; PEI can be released uniformly within 25 days.	Optimal temperature: 60 °C; 50–90 °C–normal gelation occurs; >70–90 °C–performance significantly declines

### 2.3. Comparison of Plugging Performance of PAM Systems in Oil Fields

To intuitively compare the performance and applicable scenarios of various crosslinkers, mainstream oilfield plugging crosslinkers are classified herein, and their key properties are summarized in Table 4, including gelation time, strength grade, long-term stability, and applicable temperature range.

As summarized in Table 4, conventional cross-linkers feature low cost for routine reservoirs but suffer from poor adaptability to HTHS conditions. By contrast, specialized functionalized cross-linkers are developed to overcome such drawbacks, targeting HTHS compatibility, deep-reservoir delayed gelation and environmental responsiveness. Distinct performance among subtypes enables targeted formulation selection for practical reservoir conditions.

It should be noted that the data compiled in Table 4 are extracted from independent laboratory investigations, where experimental parameters (including polymer concentration, crosslinker loading, salinity, pH, aging duration, shear history, and characterization protocols) vary substantially across individual studies. Test temperatures also span a wide range from 30 °C up to 200–224 °C. For this reason, this table is intended only for qualitative comparison of performance boundaries rather than absolute quantitative benchmarks for field-scale formulation design. Direct numerical comparison of these datasets should be treated with caution, as differing experimental setups may introduce ambiguity in performance interpretation.

According to Table 4, organic cross-linkers exhibit favorable thermal stability and high gel strength, suitable for HTHP high-permeability reservoirs at 70–150 °C [67,72] with widely tunable gelation time. Although metal-containing cross-linkers can gel at 140 °C within 6–27 h, excessive cross-linking kinetics above 70 °C prevent delivery of working fluid to target intervals [55]. Consequently, metal-ion cross-linked gels are mainly restricted to medium-low-temperature reservoirs below 70 °C despite their high-temperature gel-forming capability in laboratory tests.

Specialized functionalized cross-linkers deliver targeted performance gains while retaining inherent limitations. Nano-composite cross-linkers achieve a high breakthrough pressure of 21.71 MPa yet possess a narrow verified temperature window [73]. Hydrophobic-association cross-linkers offer self-healing properties and remain stable at 70 °C without irreversible bond degradation below 250 °C. Delayed-release cross-linkers realize gelation times up to 17 days for deep profile control, whereas gel performance degrades markedly above 70–90 °C [62]. Supramolecular intelligent cross-linkers support rapid gelation and outstanding shear resistance at ultra-high temperatures, showing promise for 200–224 °C ultra-deep reservoirs [74].

It should be emphasized that temperature alone is an insufficient metric for evaluating the field applicability of crosslinkers. Although metal-ion-crosslinked gels deliver favorable performance below 70 °C under laboratory conditions, real-world field performance is governed by a complex interplay of reservoir and operational factors: formation water salinity and ionic composition, divalent ion concentration, reservoir pH, matrix permeability, fracture aperture, rock mineralogy, in situ oil saturation, polymer adsorption-retention, pumping-induced shear degradation, wellbore residence time, and formation fluid compatibility [53]. Temperature serves only as one key boundary constraint; laboratory-validated high-temperature formulations do not guarantee satisfactory field outcomes if they fail to withstand high-divalent brine, intense mechanical shear, or strong rock-surface adsorption [53].

Beyond thermal-performance-based material ranking, crosslinker selection must balance rheological performance against environmental and occupational risks. PEI forms robust covalent networks with good thermal stability, yet poses handling hazards from amine irritancy, limited biodegradability, and special treatment requirements for residual PEI in produced water. Phenolic crosslinkers carry well-documented risks of toxic/carcinogenic free phenol-formaldehyde species, subsurface persistence, and strict produced-water treatment demands [42]. Cr^3+^-based gels, while far less hazardous than Cr^6+^, require proper management of chromium-bearing flowback fluids due to concerns over mineral adsorption, formation damage, and tubular corrosion [42]. For nano-composite-modified systems, performance gains are accompanied by unresolved uncertainties regarding nanoparticle persistence, porous-media migration, and formation-damage risks. Newer delayed-release crosslinkers reduce on-site handling risks, but their degradation byproducts and long-term environmental impacts remain poorly characterized [11]. Overall, gel system screening must comprehensively account for intrinsic material properties, multi-dimensional reservoir constraints, and environmental-safety trade-offs.

**Table 4:** Summary Table of Core Parameters.

Crosslinker Category	Gelation Time	Strength Grade	Long-Term Stability	Temperature Range	Ref
Nano-composite Crosslinker	PAM/PEI: 25–64 h; PAMGO/PEI: 4–14 h	21.71 MPa breakthrough pressure	Stable at 30 °C	30 °C (no wide range data)	[33]
Hydrophobic Association Crosslinker	8 h for basic self-healing, 12 h for stable strength (70 °C)	0.67 MPa tensile stress; 16.26 MPa compressive stress; 3.45 MPa pressure bearing	No degradation/dehydration/shrinkage after long-term placement at 70 °C	70 °C (medium-low temp); no irreversible bond damage below 250 °C	[75]
Delayed Slow-Release Crosslinker	Adjustable to 17 d (60 °C, 6000 mg/L salinity)	Grade F (Sydansk classification), high-deformability non-flowing gel	Sustained-release crosslinker (SRC) emulsion stable for 20 d (60 °C); PEI uniformly released within 25 d	Optimal 60 °C; normal gelation 50–90 °C; strength drops significantly above 70 °C	[11]
Supramolecular Intelligent Crosslinker	10 min for stable hydrogel (60 °C)	122 mPa·s viscosity after 147 min shearing (200 °C, 170 s^−1^)	No obvious degradation after 147 min continuous shearing (200 °C)	200–224 °C (ultra-high temperature)	[76]
Organic Crosslinker	Adjustable from tens of minutes to tens of hours (70–150 °C)	Highest grade (Code I)	Modified PEI system maintains high strength 30 d after gelation	70–150 °C (high temp reservoirs)	[29,40]
Metal Crosslinker	Adjustable 6–27 h (140 °C)	Cr^3+^ Grade H	G′ and G″ basically stable within 15 d (140 °C); thermal stability lower than organic crosslinked gel	Excessively fast reaction above 70 °C; suitable for medium-low temp reservoirs below 70 °C	[31]

Figure 22 summarizes the reservoir adaptability and gel performance of four crosslinker categories, with data extracted from four independent literature sources [25,77,78,79]. It should be emphasized that different characterization methods for gel strength were adopted across these four studies, which prevents direct quantitative cross-comparison of gel strength values. The gel strength of the organic crosslinker was characterized by G′ via oscillatory shear rheology [77]. The gel performance of the metal ion crosslinker was evaluated using breakthrough pressure, rather than rheological G′ data [78]; The gel strength of the nano-composite crosslinker was measured as G′ by oscillatory shear rheology [25]; While the gel strength of the sustained-release delayed crosslinker was determined as G′ using a high-temperature, high-pressure rheometer [79].

The mineralization and temperature data are comparable, but the gel strength metrics are not consistent. Therefore, the gel strength values in the figure are only reported as originally presented in each reference and cannot be used for strict quantitative comparison. The figure mainly demonstrates the difference in reservoir tolerance among the four crosslinker systems.

### 2.4. Environmental Adaptability of Crosslinkers

Different cross-linking systems vary greatly in reservoir compatibility, operational safety, ecological risks, and produced-water disposal demands [80]. Most current investigations prioritize gel mechanical performance, gelation kinetics, thermo-salinity resistance, and plugging efficiency [11,12,80]. By contrast, environmental performance is frequently only described via vague qualitative labels such as “eco-friendly” or “low-toxic”. For field applications, the ecological risk of PAM gels is multi-faceted, encompassing toxicity, biodegradability, environmental persistence, and corrosivity. Accordingly, balanced and systematic environmental assessments are urgently needed for PEI-based, Cr^3+^-based, phenolic-formaldehyde, nano-composite-modified, and novel delayed-release cross-linking systems, instead of relying merely on oversimplified qualitative descriptions [80].

Table 5 compiles key environmental-safety metrics for these five cross-linking categories based on published laboratory and field studies, covering acute/chronic toxicity, biodegradability, environmental persistence, and corrosivity.

From the form, we can draw the following conclusions:

- PEI-cross-linked gels exhibit excellent viscoelasticity and thermal stability up to 110–130 °C with low matrix damage risk. Nevertheless, branched PEI is poorly biodegradable, and residual free PEI in flow-back water causes chronic aquatic toxicity, requiring specialized wastewater treatment for onshore and offshore fields [11,80].
- Cr^3+^-based gels achieve rapid gelation and high strength for medium-low-temperature reservoirs. Though less toxic than Cr^6+^, non-biodegradable Cr^3+^ may oxidize into toxic Cr^6+^ under aerobic surface conditions [5]. Strict discharge regulations restrict their deployment in eco-sensitive regions.
- Phenol-formaldehyde (HQ-HMTA) gels feature prominent high-temperature resistance [42]. Their major hazard arises from volatile, suspected-carcinogenic unreacted monomers during mixing. While cross-linked resin matrices are less toxic, recalcitrant resin fragments persist underground. Low-toxicity phenolic alternatives (e.g., THMBPA) have been proposed, yet their long-term environmental fate remains unvalidated.
- Nano-composite-modified PAM gels (nano-SiO_2_, MG, TOCN, T-ZnOw) possess enhanced thermo-salinity tolerance and ageing stability [7]. However, non-biodegradable inorganic nanofillers may agglomerate to plug pore throats. Insufficient field data on nanoparticle migration and ecotoxicity impedes their large-scale implementation.
- Novel delayed-release and biomass-derived cross-linkers enable tunable gelation for deep in situ conformance control with low acute toxicity. Still, degradation pathways, by-product toxicology, and produced-water disposal remain largely lab-scale, lacking field ecological datasets [8].

In summary, simple labels such as “green” or “low-toxicity” cannot guide practical material selection [3]. Future evaluation should integrate standardized environmental-safety tests with rheology, gelation kinetics, and core-flood experiments, covering toxicity, biodegradability, persistence, corrosivity, and formation-damage risks. Moreover, reservoir geochemistry (salinity, pH, mineralogy) modulates actual ecological hazards; site-specific risk assessment is therefore indispensable for gel-system screening.

## 3. Modified Gel Formulation System

Nanomaterials possess intrinsic features including small-size effects, large specific surface areas, high surface activity, and tunable physicochemical properties. They have become critical functional materials to address application bottlenecks in harsh oil reservoirs and upgrade conventional functional materials within oilfield chemistry. Nanomaterials such as nano-SiO_2_, nano-titanium dioxide, graphene, and nano-cellulose can improve multiple gel properties, such as temperature resistance, salt tolerance, mechanical strength, viscoelastic modulus, and plugging efficiency [84,85,86]. When nanoparticles are incorporated into gel solutions, they embed within polymer coils. As gelation proceeds, nanoparticles aggregate and form micro-scale arrangements, which enhance the structural strength and apparent viscosity of gel solutions.

### 3.1. SiO_2_ Modification

Among various nano-modified functional materials applicable to oilfield development scenarios, nano-SiO_2_ is currently the most systematically researched and field-matured mainstream inorganic nanomaterial. This material consists of amorphous silica particles with a particle size ranging from 1 to 100 nm. It offers advantages such as a wide range of raw material sources and low preparation costs, while simultaneously possessing excellent chemical and thermal stability. Its surface is rich in a large number of active silanol groups, allowing for flexible surface functionalization modification according to the application requirements of the target system. Additionally, nano-SiO_2_ exhibits prominent mechanical rigidity, enabling it to simultaneously achieve multiple key functions, including physical reinforcement, auxiliary crosslinker, and microstructure regulation in organic polymer systems [87]. Ahmed et al. [88] conducted a systematic review of the latest research advances in the application of nanomaterials in oilfield water shutoff and profile control. They pointed out that nano-SiO_2_, through its surface-rich active sites, forms multiple interactions with polymer chains, which can significantly enhance the thermal stability and salt resistance of gels.

Under the harsh reservoir conditions of HTHS, polymer systems face key technical bottlenecks such as easy hydrolysis and segment curling of polymer chains, collapse and destruction of cross-linked network structures, insufficient long-term thermal stability, and easy dehydration shrinkage. Given the prominent modification and enhancement potential of nano-silicon dioxide, its application for strengthening and modifying HPAM-based gel systems has become a research hotspot in the field of oilfield chemistry. Existing studies have conducted extensive systematic experiments on the performance regulation rules and modification enhancement mechanisms of nano-SiO_2_ for HPAM-based gel systems.

Zou et al. [89] introduced CTAB-modified (C-SiO_2_) into PAM/PEI gels. The study demonstrated that the incorporation of SiO_2_ enables the carbonyl groups in PAM molecules to form ionic bonds with the quaternary ammonium groups on the surface and hydrogen bonds with silanol groups. The synergistic effect of these interactions maintains the extended conformation of PAM molecular chains under high-salt conditions, preventing curling and thereby enhancing the gel’s network structure.

Experiments by Liu et al. further demonstrated that the addition of SiO_2_ significantly affects the gelation kinetics and mechanical properties of the gel. As temperature or content increases, gel time decreases: adding 1% SiO_2_ under 80 °C conditions reduces gel time to 8 h; adding 1% SiO_2_ under 150 °C conditions reduces it to 6 h. Additionally, the addition of 0.3 wt% SiO_2_ can increase the surface viscosity of the gel by more than twice [89].

Furthermore, hydrogen bonds in SiO_2_ can also slow down the hydrolysis of amide groups. Liu et al. [89] systematically investigated the effect of nano-SiO_2_ on the thermal stability of PAM/HQ-HMTA composite gels using differential scanning calorimetry (DSC). DSC test results showed that the bound water content in gels without SiO_2_ was 22.5%, whereas after adding 0.3 wt% SiO_2_, the bound water content increased to 39.9%. The maximum withstanding temperature of the gel was elevated from 137.8 °C to 155.5 °C. This confirms that binding with water molecules and hydrated protons through hydrogen bonds and electrostatic interactions increases the proportion of bound water, thereby enhancing the gel’s water-holding capacity and thermal stability. Xu et al. [90] further investigated the stability mechanism of nano-reinforced HPAM hydrogels using DSC combined with molecular dynamics simulation. The results indicate that SiO_2_ enhances the interaction between water molecules and the colloidal phase, which is the primary reason for improving the stability of the nano-composite hydrogel. Wu et al. [91] confirmed through DSC testing that the addition of nanomaterials can enhance the water-holding capacity of gels and effectively inhibit dehydration shrinkage. The gel strength with 0.4 wt% nanomaterials was 4 times higher than that of the gel without nanomaterials. Nanoparticle-reinforced gels also exhibited excellent extensibility and toughness, with a high yield stress, which is beneficial for plugging performance in field applications. The portion of bound water in the gel without adding nanoparticles is around 21.32%, while it almost doubled for the gel with 0.4 wt% of nanoparticles. Generally, the bound water is tightly bonded to the network of the gel, and it is hard to be extruded from the gel; this is probably why the addition of nanoparticles can inhibit the syneresis of the gel. Zareie et al. [92] evaluated the effects of nanoparticle concentration on the crosslinker enthalpy, crystallinity, morphology, and viscoelastic properties of crosslinked polyacrylamide hydrogels using DSC, X-ray diffraction (XRD), scanning electron microscopy (SEM), and rheological analysis. The study results indicated that the introduction of nanoparticles significantly altered the hydrogel network structure, and the dual-network hybrid hydrogels exhibited effective improvements in network stiffness, viscoelasticity, and mechanical properties.

In terms of rheological properties, within the frequency range of 0.1 to 100 rad/s, the ratio of G′ to G″ increases with the addition of silica nanoparticles to the gel solution [93]. In the gel solution, -CONH_2_ groups from the polymer crosslink with -CH_2_OH groups from the crosslinker, which facilitates the formation of a uniform porous network structure that provides the primary storage space for water. Through oscillation experiments, hydrophilic pyrogenic silica, characterized by silanol groups on its particle surface, can form hydrogen bonds with carboxyl and amide functional groups of cross-linked polymers. It can be seen from Figure 23 that the gelation time of the base gel remains almost unchanged with the addition of hydrophilic nano-silica (HCSIL200). At a dosage of 0.4 wt%, as shown in Table 6, the USS nearly doubles, and a further increase is observed at 0.5 wt%. Besides, when the HCSIL200 dosage reaches 0.5 wt%, the USS value increases by nearly three times compared with the base gel [94].

**Figure 23:**
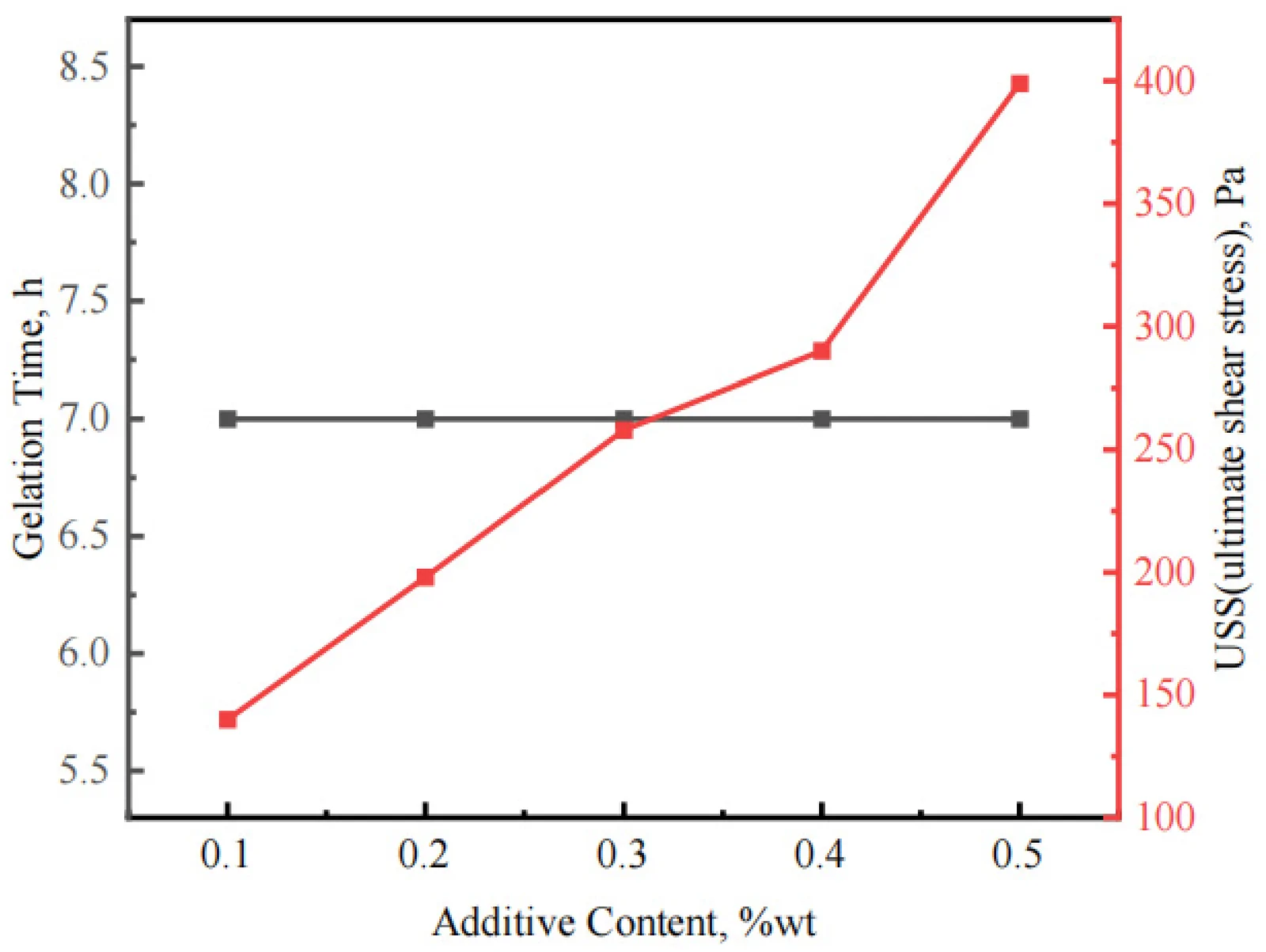
Effect of nano-SiO_2_ additives on gelation time and USS [94] (Reproduced with permission from A. Telin, F. Safarov, R. Yakubov, et al. *Gels*; published by MDPI, 2024).

**Table 6:** Results of oscillation experiments.

Composition	Elastic Modulus G′, Pa
Base gel	36.1
Gel + 0.4%wt HCSIL200	57.3

Data from *Thermal Degradation Study of Hydrogel Nanocomposites Based on Polyacrylamide and Nanosilica Used for Conformance Control and Water Shutoff*. Gels; A. Telin, F. Safarov, R. Yakubov, et al.; MDPI; 2024.

A large number of hydroxyl groups on the SiO_2_ surface significantly enhances the hydrophilicity and thermal stability of the gel. These hydroxyl groups can not only form hydrogen bonds with polymer chains but also retard the hydrolysis of amide groups. As shown by the DSC experimental results in Figure 24, the bound water content in the gel without SiO_2_ is 22.5%, whereas it increases to 39.9% after adding 0.3 wt% SiO_2_, further confirming the role of SiO_2_ in improving the thermal stability of the gel. Figure 25 shows the micro-characterization images of gel samples prepared with and without the addition of 0.2 wt% silica nanoparticles. It can be seen in Figure 25c,d that the one with nanoparticles has a well-ordered structure, similar to lamellae. Whereas, for the gelant without nanoparticles, its network structure is sprawling and some parts of the backbone are quite thin, as shown in Figure 26.

The reinforcement mechanisms of diverse nanomaterials differ substantially. For TiO_2_-modified systems, existing studies indicate that incorporating 0.01 wt% TiO_2_ remarkably enlarges the peak area of EOH, which promotes the generation of surface titanium-hydroxyl groups. Such structural evolution improves the thermal stability of polymer gels and compacts the micro-network architecture.

By contrast, for nano-SiO_2_-containing gel systems, SiO_2_ nanoparticles tend to rearrange and align regularly within polymer chain bundles and gel networks after incorporation. Takeno et al. [37] investigated composite systems consisting of polyacrylic acid-tannic acid (PAAS) gel, clay, and SiO_2_. Their results demonstrated that Young’s modulus rises with increasing SiO_2_ loading, which implies a higher number density of effective cross-linking points within the gel network. Nevertheless, their QCM and FTIR characterizations further revealed that SiO_2_ nanoparticles do not participate in chemical-bond-mediated network construction; namely, no chemical cross-links are formed between SiO_2_ and other gel components. Complementary SAXS measurements showed that clay platelets can be well-dispersed inside the gel matrix, while the electrostatic repulsion between SiO_2_ and PAAS significantly modulates the spatial distribution of PAAS polymer chains [41].

In summary, nano-SiO_2_ delivers prominent reinforcement in HPAM/PEI gel systems, yet its working mechanism is strongly system-dependent. Under certain formulations, SiO_2_ can interact with polymer matrices via ionic bonds and hydrogen bonds to participate in network assembly; in other scenarios, it merely acts as an inert physical filler or structural regulator without chemical bonding. The divergent conclusions among published studies are closely associated with variables including nanoparticle surface modification, polymer matrix type, and experimental characterization approaches. Therefore, clarifying the intrinsic interaction modes between nanomaterials and polymer matrices is essential for interpreting reinforcement behaviors and guiding the performance optimization of nano-composite PAM gels.

### 3.2. Graphene Modification

Nanographene possesses characteristics such as high-temperature resistance, high specific surface area, easy modifiability, and rigid structure. Its surface can be chemically modified to introduce active functional groups, enabling chemical bonding with polymers, thereby making it an ideal reinforcing phase for optimizing gel performance [95]. The core function of incorporating MG is to upgrade the gel from physical filler reinforcement to ‘chemical crosslinker strengthening’, with specific benefits manifested in three aspects: First, shortening the gelation time and improving crosslinker efficiency [58]. Secondly, significantly enhance the gel’s mechanical strength and thermal stability to adapt to the harsh environment of deep oil reservoirs [29]. Thirdly, optimize the plugging performance and water retention capacity to extend the gel’s validity period. Its synergistic enhancement mechanism stems from the functional synergy after chemical modification, as shown in Figure 27 [46]. Sulfonic acid groups (-SO_3_H) and hydroxyl groups (-OH) are grafted onto the surface of nano-graphite via a diazotization reaction. The hydroxyl groups undergo chemical crosslinking with the -CONH_2_ groups of PAM, forming a dense three-dimensional network. The MG layers are embedded within this network, where their inherent rigidity enhances the network strength. Meanwhile, the sulfonic acid groups improve the gel’s hydrophilicity, converting free water into bound water through hydrogen bonding, thereby inhibiting high-temperature dehydration and further consolidating structural stability.

In relevant modification studies, Michael et al. [96] first used graphite particles for gel reinforcement, but employed a physical doping method, which could only moderately enhance gel strength through particle filling and could not address structural degradation under high-temperature and high-salinity conditions. To overcome this limitation, Lu et al. [46] innovatively chemically modified nano-graphite, transforming it from a filler phase to a crosslinker. The core objective was to improve the durability and stability of the reinforcement effect through chemical bonding, thereby creating a differentiated innovation compared to the traditional research path focused on polymer modification. The addition of MG achieved a significant breakthrough in the core performance of the gel. Table 7 presents a comparison of the core performances between conventional gels and hybrid gels.

In terms of gelation performance, the addition of MG significantly reduces gelation time and enhances mechanical strength. As the MG concentration increases, the gelation time gradually decreases, reaching an optimal value of 4 h at 0.2 wt% MG, which is 50% shorter than that of conventional gels. This is because MG provides more crosslinker sites, thereby improving reaction efficiency. Rheological tests (Figure 28) show that the G′ and G″ of MG hybrid gels are increased by more than 2 times, and G′ remains greater than G″, maintaining good solid-like characteristics. Figure 29 indicates that after aging at 140 °C for 60 days, the modulus of the hybrid gel in Figure 29a shows no significant decrease, whereas the modulus of the conventional gel in Figure 29b exhibits a significant attenuation, confirming that MG substantially enhances the mechanical stability of the gel.

In contrast to the covalent bonding pathway where nano-graphite is used as a crosslinker core through chemical modification, Cao et al. [97] employed GO nanoparticles as reinforcing phases to physically modify conventional partially hydrolyzed polyacrylamide (HPAM) polymer flooding systems via non-covalent interactions. This approach does not require altering the original on-site construction process of polymer flooding and can be directly adapted to existing oilfield injection procedures, making it more suitable for large-scale promotion and application in medium-to-high permeability reservoirs. The core efficiency-enhancing mechanism originates from the synergistic non-covalent interactions between oxidized graphite nanoparticles and polyacrylamide molecules. Spectral analysis confirmed that oxygen-containing functional groups (such as hydroxyl and carboxyl groups) on the surface of GO nanosheets can form stable electrostatic hydrogen bonds with amide/carboxyl groups on HPAM molecular chains, accompanied by a small amount of covalent crosslinker. This hydrogen bonding interaction differs from irreversible covalent bonds in chemical crosslinkers, exhibiting dynamic reversibility—when the gel system is subjected to high shear forces during formation flow, hydrogen bonds can temporarily break to dissipate energy; once the shear stress is eliminated, hydrogen bonds can rapidly re-form and recover [98]. This dynamic self-healing mechanism fundamentally alleviates the irreversible viscosity loss problem of conventional polyacrylamide systems during formation flow processes.

Based on this modified system, the study conducted multiple indoor physical simulation experiments to systematically verify the enhancement of its core performance. Rheological viscosity-increasing performance is the core fundamental property of polymer flooding systems, directly determining the system’s oil-water mobility ratio control capability and macroscopic sweep volume. The study clarified the viscosity-increasing effect of the modified system through gradient concentration rheological tests. Using the HAAKE MARS 60 high-temperature and high-pressure rheometer, the apparent viscosity was measured across the complete shear rate range of 0.01~1000 s^−1^, fully matching actual field conditions such as wellhead pumping and formation seepage in oilfields. Additionally, three key shear rates were selected: 0.01 s^−1^ (formation low-velocity seepage), 7.34 s^−1^ (reservoir characteristic shear rate), and 1000 s^−1^ (wellhead high-shear), to quantify the influence of polymer concentration on the system’s apparent viscosity. The core test results are shown in Figure 30.

The final experiment comprehensively evaluated the viscosity-enhancing effect, field injectivity, and economic cost, and selected 0.20% as the base polymer concentration for subsequent modified experiments with graphene oxide nanoparticles. Further rheological property tests of the graphene oxide nanoparticle-modified system were conducted, as shown in the following Figure 31.

Test results indicate that all modified systems retain the typical shear-thinning pseudoplastic fluid characteristics. Compared to pure polymer systems, the apparent viscosity of the modified systems is significantly increased at the same shear rate. As the dosage of graphene oxide nanoparticles increases, the overall rheological curves shift upward, confirming that graphene oxide nanoparticles can combine with polymer molecules through hydrogen bonding, strengthen the three-dimensional network structure of the polymer, and effectively improve the rheological thickening performance of the system.

### 3.3. Nanocellulose Modification

TOCNs are nano-scale modified materials derived from plant fibers. Their surfaces are rich in hydroxyl (-OH) functional groups, and they possess core properties including high mechanical strength, high aspect ratio, excellent biocompatibility, and renewability with biodegradability [7]. This unique structural advantage endows TOCN with excellent application value in the modification of oilfield water-shutoff gels: on one hand, the high aspect ratio structure can serve as a skeleton support to enhance the physical stability of the gel; on the other hand, the surface-active functional groups are readily capable of undergoing crosslinker reactions with gel matrices such as PAM and Slg, while also meeting the demand for environmentally friendly materials in green oilfield development. It can specifically address key drawbacks of traditional PAM gels, including weak mechanical strength, insufficient high-temperature stability, and poor biodegradability [100].

The modification of gels by TOCN is achieved through a synergistic multi-mechanism involving chemical crosslinking, physical support, and hydrogen bonding, all of which have been confirmed by experimental characterization methods. In terms of chemical crosslinking, the hydroxyl groups on the TOCN surface can react with PAM monomers (AM) and the crosslinker N,N′-methylenebisacrylamide (MBA) to form C-O-C covalent bonds. As the TOCN content increases, the crosslinker density of the gel significantly improves. Regarding physical support, TOCN, as a nanoscale skeletal material, is uniformly dispersed within the gel matrix. Through fiber interweaving, it forms a physical support bridge structure, effectively densifying the gel network. SEM characterization results show that the STP-5 sample with high TOCN content exhibits a uniform porous network morphology, whereas the STP-1 sample with low TOCN content has a loose network structure (see Figure 32b,f). In terms of hydrogen bond synergy, TOCN can form a large number of hydrogen bonds with PAM molecules, further enhancing the structural stability of the gel network.

The introduction of TOCN significantly optimizes multiple core properties of the gel. Through the synergistic effect of increased crosslinker density and physical support, the compressive strength and G′ of the gel are effectively improved (Figure 33). G′ exhibits a concurrent increasing trend, and the strain tolerance limit of all samples reaches 1000%, which meets the usage standards for high-shear oilfield production environments [7]. In samples with low TOCN content, insufficient crosslinker reaction sites result in a loose network structure, leading to weak mechanical properties. In contrast, samples with high TOCN content form a dense and stable three-dimensional cross-linked network through the synergistic effect of TOCN, thereby achieving significant optimization of mechanical properties [7].

The incorporation of TOCN also significantly enhanced the high-temperature stability and salt tolerance of the gel. Its microstructure transitioned gradually from a smooth and loose state to a uniform and dense porous network structure. The swelling ratio decreased from approximately 1100% to 280% (swelling curves are shown in Figure 34c). Swelling is a physical phenomenon where gels absorb liquid and undergo volume expansion in a solvent. This change indicates that a more compact and stable cross-linked network structure has been formed. The composite gel can maintain structural integrity for up to 108 h in a 140 °C saline environment (high-temperature durability is shown in Figure 34a), demonstrating excellent long-term thermal stability.

Existing relevant studies further verify the universal advantages of TOCN in the field of gel modification: Chen et al. prepared double-network gels using a composite method of TOCN and PAM, effectively enhancing the tensile strength of the gel to meet the mechanical performance requirements of water-blocking materials; Li et al. [45] incorporated TOCN into the PAM/gelatin hydrogel system, which not only improved the mechanical properties of the hydrogel but also endowed the material with rapid shape memory function Yang et al. [43] successfully enhanced the shear deformation resistance of gels by reinforcing the hydrogel network density with TOCN. The aforementioned research results indicate that TOCN can optimize the comprehensive performance of gels through various modification strategies, providing diversified technical directions for the development of high-performance oilfield water-blocking materials.

### 3.4. T-ZnOw Modification

T-ZnOw are three-dimensional nano-reinforcing materials with a tetrapod structure, characterized by needle lengths of 5 μm to 30 μm and root diameters of 0.5 μm to 5 μm. They possess low density, high specific surface area, excellent mechanical support, and good dispersion stability. Compared to traditional spherical nanoparticles (e.g., nano-SiO_2_) or two-dimensional nanosheets (e.g., graphene oxide), the three-dimensional tetrapod structure of T-ZnOw offers unique advantages in gel network reinforcement. Geometrically, spherical nanoparticles primarily anchor onto polymer chains through point contact or hydrogen bonding, with their reinforcing effect dependent on uniform dispersion within the matrix. Two-dimensional sheet materials such as GO, although capable of forming hydrogen bonds or covalent bonds with polymer chains via surface functional groups, exhibit reinforcement mainly along the in-plane direction of the sheets. In contrast, the tetrapod structure enables the establishment of multi-dimensional physical anchoring points within the gel network—each needle can independently embed between polymer chain bundles, forming a space-bridging structure analogous to a three-dimensional rivet. Its synergistic advantages in structure and performance make it an excellent nanofiller for modifying polyacrylamide gel systems. can optimize the gel network structure at the micro level through multi-dimensional binding with polyacrylamide molecular chains, compensating for the performance shortcomings of pure polyacrylamide gels in complex reservoir environments. This significantly enhances the gel’s temperature resistance, shear resistance, structural stability, and long-term service capability, providing a performance improvement pathway for its efficient application in oil and gas development fields.

Ma et al. [7] explored the reinforcement of polyacrylamide gels using tetrapod zinc oxide whiskers to enhance their structural stability and bound water content. Thermal stability tests conducted at 130 °C for 60 days, as revealed by micro-characterization results in Figure 35, showed that unmodified gels exhibited a strength reduction to grade E after aging, with an elastic modulus retention rate of only 44.6% (maximum elastic modulus 6.2 Pa), significant dehydration, a peak inflection temperature of 126.72 °C, increased microscopic network defects, and a loose structure. In contrast, modified gels containing 0.15 wt% maintained a strength of grade F after aging, achieved an elastic modulus retention rate of 76.5% (maximum elastic modulus 21.8 Pa), showed no significant dehydration, had a peak inflection temperature elevated to 156.67 °C, and possessed an intact and dense microscopic network. This confirms that the incorporation significantly densifies the polyacrylamide gel network, forming a more compact three-dimensional cross-linked structure; it exhibits stronger interactions with polymer chains, thereby effectively inhibiting the degradation and structural collapse of polymer chains under high-temperature conditions. By providing rigid support to suppress polymer chain degradation, it increases the bound water content of the gel, reduces high-temperature dehydration shrinkage, and ensures its long-term water-blocking performance.

Apart from gel-reinforcement purposes, polymer-nano system combinations can also deliver synergistic enhanced-oil-recovery benefits in carbonate reservoirs via wettability alteration and interfacial-tension reduction [101,102].

The reinforcement performance of these nanomodified fillers is not determined merely by their intrinsic material structures. Particle morphology, surface functional groups, and filler loading dosage jointly dominate the comprehensive performance of final PAM gels. It should be noted that promising mechanical and thermal performance obtained under laboratory conditions cannot be directly extrapolated to real reservoir environments. Table 8 summarizes the key features and performance enhancement effects of these representative nano-modifiers.

### 3.5. Artificial-Intelligence-Assisted Modification of PAM Gels for Oilfield Applications

Different from the above-mentioned material-driven modification strategies (nano-SiO_2_, graphene, TOCN and T-ZnOw) that rely on laboratory trial-and-error for formula tuning, artificial intelligence (AI) provides an emerging data-driven paradigm for oilfield-oriented PAM-gel research. Strong interactions among multiple variables, including nanoparticle dosage, cross-linker concentration, salinity, and temperature, generate numerous experimental combinations and increase laboratory workload. Recent advances in AI have enabled new approaches for simulating gelation behavior, predicting gel performance, and optimizing operating parameters for profile-control and water-shut-off PAM gels.

Combining AI with reservoir numerical simulation enhances prediction and decision-making for plugging treatments. Digital-twin models can numerically reproduce the full process of PAM gels, covering transportation, in situ reactions, and plugging behavior inside porous media. Arrhenius-based kinetic models such as the Hurd–Letteron and Marfo models are widely used to characterize gelation of nano-composite-reinforced PAM gels [103].

Machine-learning algorithms represented by artificial neural networks (ANN) can predict the distribution and plugging efficiency of gel agents in heterogeneous formations, supporting quantitative optimization of operational parameters [104]. For example, Xianchao et al. [105] coupled reservoir-wellbore numerical models to forecast gel performance for horizontal-well water shut-off and achieved pre-evaluation of field treatment responses.

Deep learning and reinforcement learning can mine information from historical operational datasets to optimize gel formulations and injection schemes, facilitating reservoir-targeted customized treatment designs. These advances push water-shut-off technology from passive plugging toward proactive intelligent regulation, providing new insights for efficient and sustainable hydrocarbon production.

Nevertheless, AI-assisted PAM-gel research still faces critical limitations. Most models are trained on small-scale laboratory datasets, and inconsistent experimental standards hinder model generalization and cross-dataset transfer. Although AI supports performance prediction and parameter optimization, it cannot interpret underlying modification mechanisms such as hydrogen-bond evolution and network reconstruction. Accordingly, AI serves as a powerful auxiliary tool instead of a substitute for experimental characterization and mechanism-focused research. Future efforts should prioritize building standardized shared datasets, developing mechanism-informed machine-learning frameworks, and strengthening the linkage between laboratory gel models and field reservoir data to promote the on-site application of AI-optimized PAM-gel systems.

## 4. Conclusions

This paper reviews the research progress of PAM gels in the fields of oilfield profile control and hydraulic isolation, and systematically summarizes the crosslinker mechanisms, system property differences, enhancement rules of nano-modification, and artificial intelligence-assisted optimization technologies of PAM gels. The main conclusions are as follows:

1. PAM water shutoff gels can form three-dimensional network structures through covalent crosslinkers, ionic coordination crosslinkers, and physical crosslinkers, and the reservoir adaptability of each crosslinker system varies significantly. Compared with the traditional HQ-HMTA system, PEI organic crosslinked gels have better viscoelasticity, with an elastic modulus up to 780 kPa at 110 °C; the Cr^3+^ ionic crosslinker system forms gel too rapidly and tends to swell above 70 °C, so it is only suitable for medium- and low-temperature reservoirs. At present, a single crosslinker system can hardly meet the comprehensive performance requirements of ultra-high temperature and high salinity deep reservoirs, and high-performance crosslinkers still require further research.
2. Nano-composite modification can effectively improve the temperature and salt resistance, mechanical strength, and long-term stability of PAM gels. Nano-SiO_2_ can raise the temperature resistance of gels to 155.5 °C; modified nano-graphite achieves a modulus retention rate of 76.5% after aging at 130 °C for 60 days; TOCN enables gels to remain stable for 108 h in a high-salt environment at 140 °C; T-ZnOw can suppress the collapse of gel networks at high temperatures via physical anchoring. However, existing studies have inconsistent interpretations of the nano-reinforcement mechanism, and experimental and evaluation standards are not unified, making it difficult to compare data horizontally and restricting the optimization and application of modified systems.
3. Artificial intelligence provides a new data-driven paradigm for the formulation optimization of PAM gels. Combined with numerical simulation and digital twin technology, it can realize gel property prediction and intelligent parameter optimization, effectively supplementing traditional experimental research. Nevertheless, current models rely on small-scale laboratory data with insufficient generalization and mechanistic interpretability, and lack large-scale field verification. They can only serve as auxiliary tools and cannot replace mechanistic research and experimental characterization.
4. The application of existing PAM gels in complex deep reservoirs still faces prominent bottlenecks. It is difficult to precisely control gelation time under high temperature and high salinity conditions, and the delayed performance of conventional delayed crosslinkers declines sharply above 90 °C. Meanwhile, academic research mostly focuses on the short-term plugging performance of gels, while studies on the long-term stability and degradation mechanism of gels under high temperature and high salinity conditions are insufficient, failing to meet the engineering requirements for long-term plugging in oilfields.
5. Environmental and construction safety should be included as core screening indicators for gel formulations. PEI, Cr^3+^, phenolic, and nano-modified systems each have advantages and disadvantages in terms of toxicity, degradability, formation damage, corrosivity, and produced water treatment difficulty. Future research and development of PAM gels should prioritize green materials with low toxicity, easy degradation, low formation damage, and good compatibility with produced water, so as to achieve coordinated improvement of plugging performance and environmental safety performance.

## 5. Outlook

To address the above limitations and promote the practical application of PAM gel systems, future research should be arranged in three stages according to short-, medium-, and long-term objectives, so as to form a systematic research path from methodology establishment to field verification.

### 5.1. Short-Term Objectives (0–2 Years)

Aiming at the core problems commonly existing in current gel systems for oilfields, including short effective duration, limited temperature and salt resistance, and poor field adaptability, multi-dimensional modification and optimization research will be carried out to focus on breaking through the technical bottleneck of long-term stability of gels. With nanoparticle modification technology as the core optimization method in this stage, systematic investigation will be conducted on the regulation mechanism of nanoparticles of different types, particle sizes, and doping amounts on gelation strength, crosslinker stability, aging performance, and long-term plugging performance of gels, so as to clarify the optimal process parameters and action mechanism of nanoparticle modification. Meanwhile, artificial intelligence-assisted modification research ideas will be introduced. Based on machine learning and data fitting algorithms, a performance prediction model for nanoparticle-modified gels will be established to rapidly screen modified formulas suitable for conventional oilfield working conditions, replacing the traditional single and inefficient trial-and-error test mode and greatly shortening the formula development cycle.

### 5.2. Medium-Term Objectives (3–5 Years)

On the basis of the short-term modification technology and accumulated experimental data, a scientific, systematic, and comprehensive standardized evaluation system for gel performance will be established. Fully considering the differences in complex field working conditions of oilfields, covering reservoir conditions such as different blocks, temperatures, salinity, permeability, and pore structures, core evaluation indicators including gelation performance, plugging performance, aging resistance, shear resistance, and long-term stability of gels will be defined, and the test methods, evaluation criteria, and grading systems for each indicator will be unified. At the same time, the artificial intelligence prediction model will be continuously optimized by incorporating multi-condition experimental data to improve the prediction accuracy of gel performance under complex reservoir conditions. Standardized evaluation specifications, databases, and intelligent prediction tools will be formed to provide technical support for the targeted selection and optimization of gel systems.

### 5.3. Long-Term Objectives (5–10 Years)

Relying on the previous modification technology research and standardized evaluation system, an integrated precise matching system of gel formula-performance-oilfield working conditions will be constructed to realize intelligent and customized development and field application of gel systems. Through big data analysis and iteration of artificial intelligence algorithms, the coupling law among oilfield working conditions, gel formulas, and service performance will be accurately revealed, forming an intelligent closed loop that can automatically match the optimal gel formula according to actual field reservoir conditions, accurately predict application effects, and reversely guide formula optimization.

## Figures and Tables

**Figure 1:**
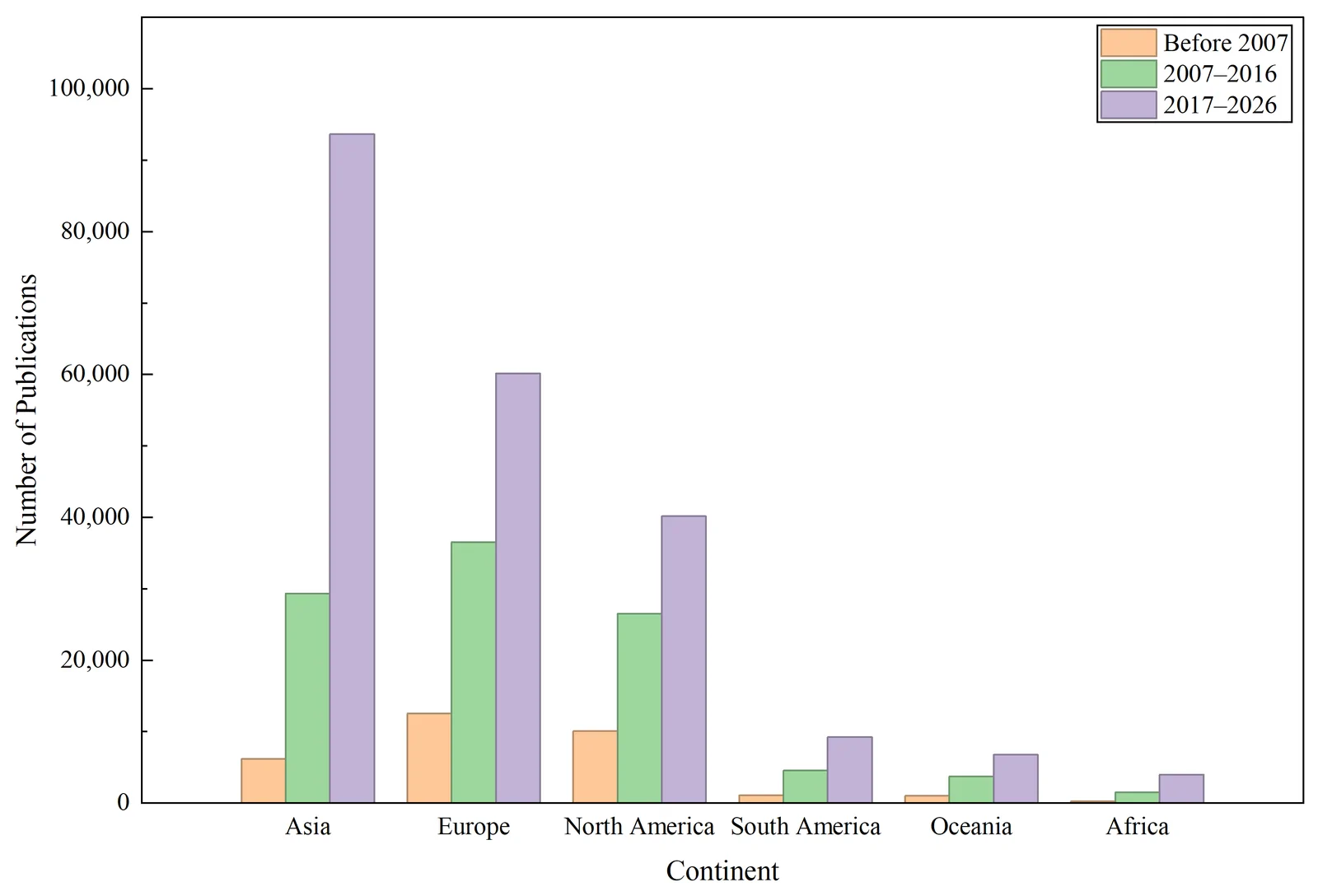
Publication distribution of PAM gel research by continent and period [25] (Reproduced with permission from Qiang Li, Xiang Yu, Lei Wang, et al. *Colloids and Surfaces A: Physicochemical and Engineering Aspects*; published by Elsevier, 2022).

**Figure 2:**
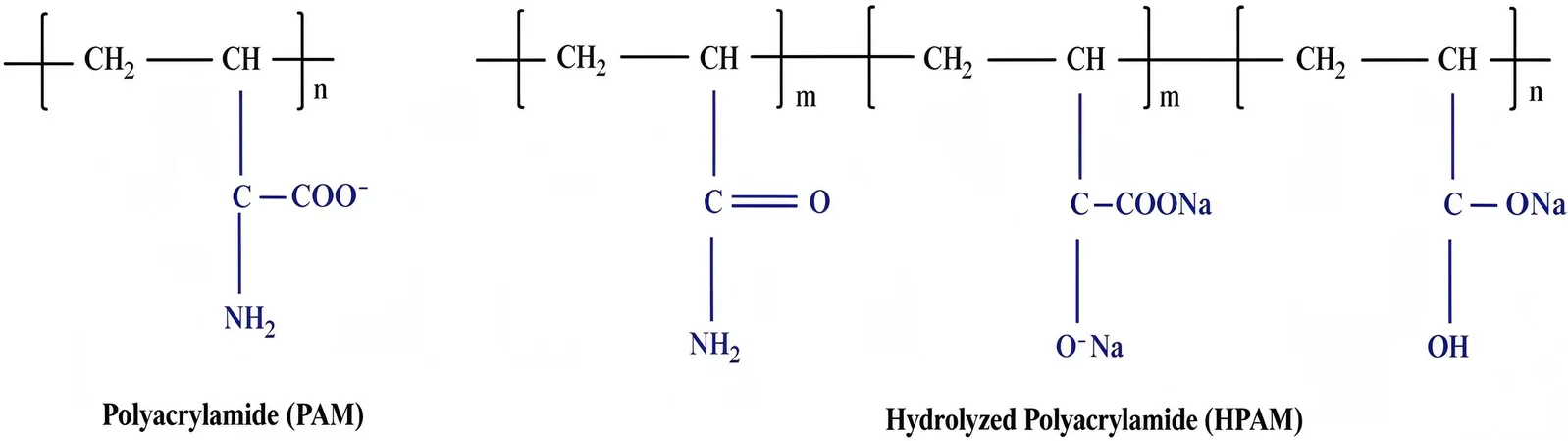
Chemical structure of PAM and HPAM [9] (Reproduced with permission from Wenkang Kang, Xu Kang, Zafar A. Lashari, et al. *Advances in Colloid and Interface Science*; published by Elsevier, 2021).

**Figure 3:**
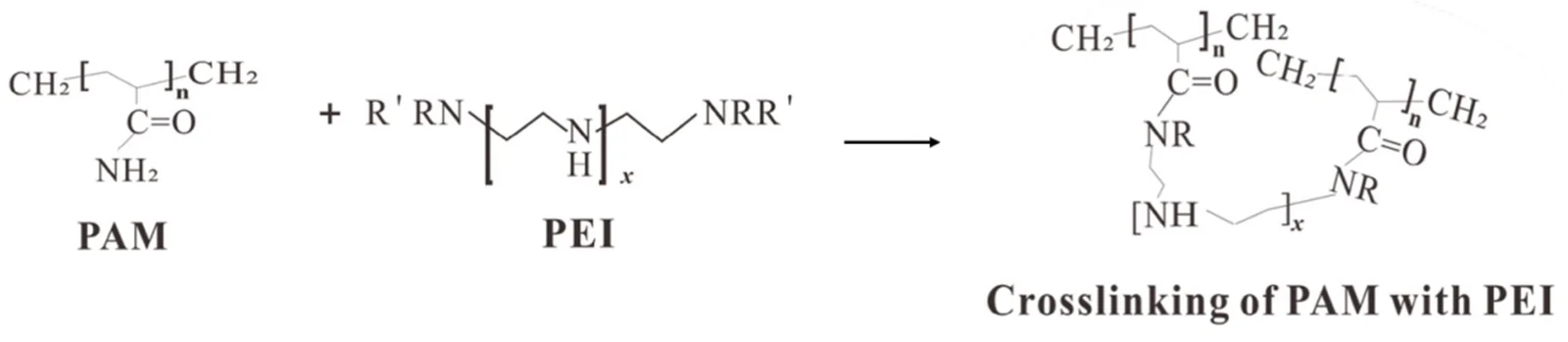
Gelation mechanism via covalent bonding: Crosslinker between PAM and PEI [29] (Reproduced with permission from Xinyue Cui, Chao Wang, Wen Huang, et al. *Journal of Physics and Chemistry of Solids*; published by Elsevier, 2025).

**Figure 4:**
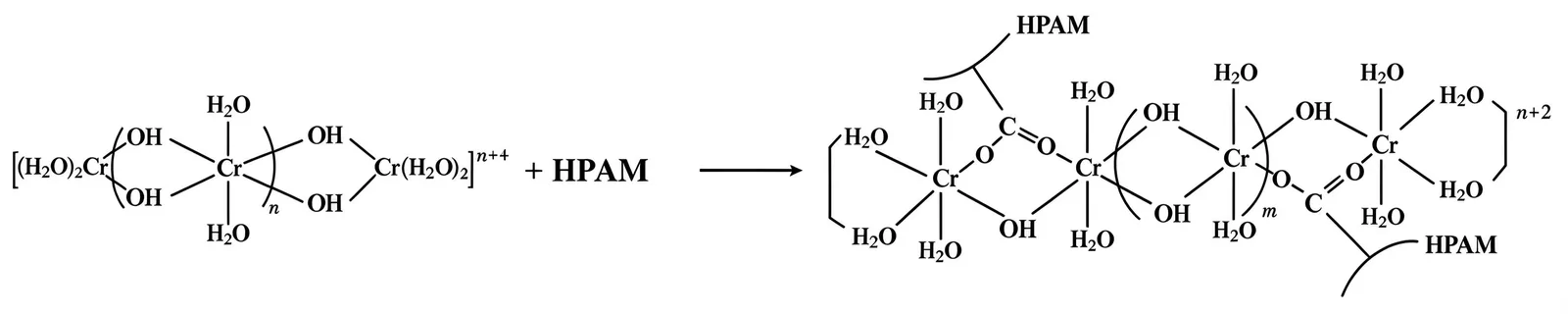
Mechanism of ionic coordination crosslinker: Crosslinker between HPAM and hydrated chromium ions [Cr(H_2_O)_6_]^3+^ [31] (Reproduced with permission from Hong Zhang, Hao Yang, B. Sarsenbekuly, et al. *Advances in Colloid and Interface Science*; published by Elsevier, 2020).

**Figure 5:**
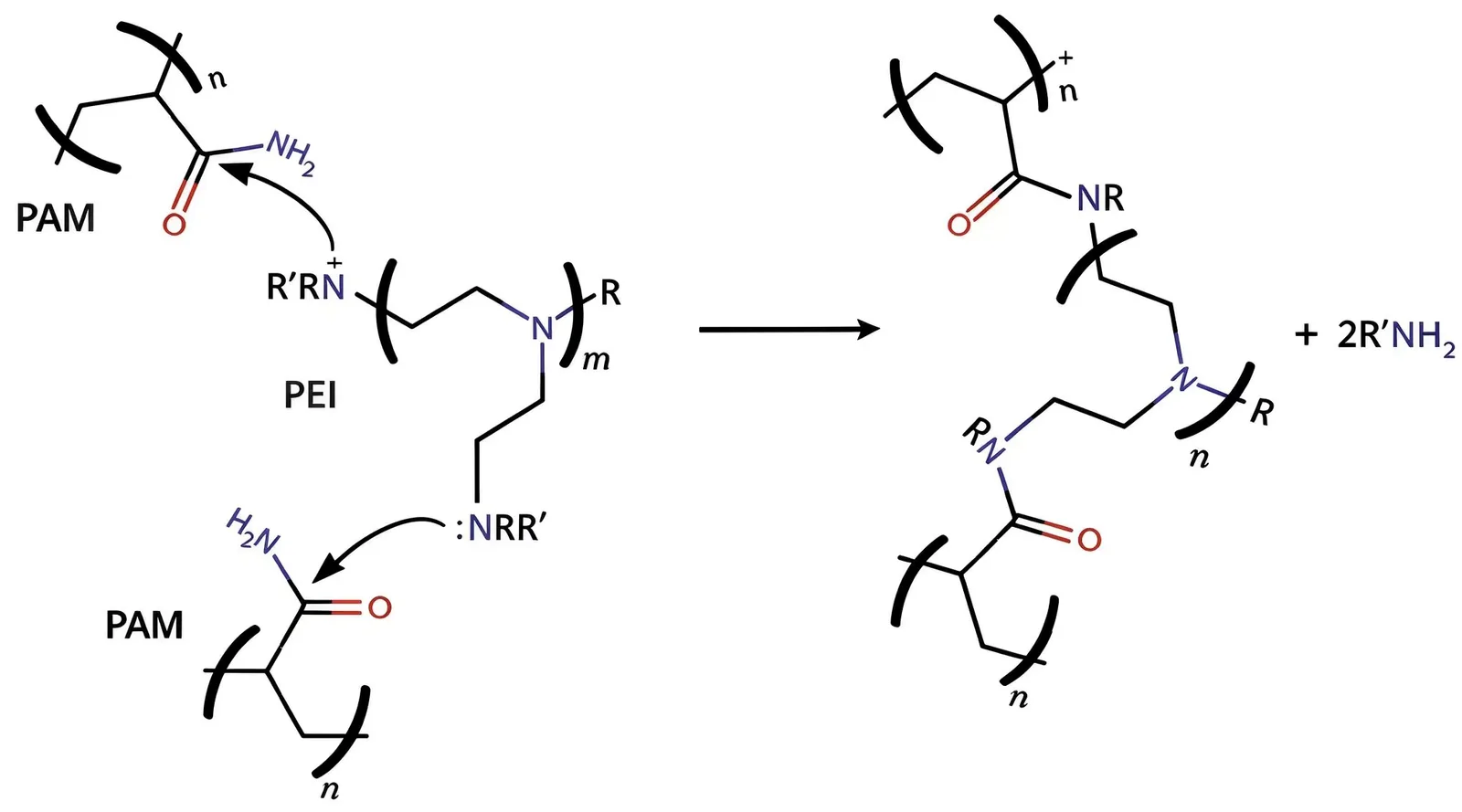
Schematic diagram of the crosslinker mechanism between PAM and PEI [43] (Reproduced with permission from Bo Yang, Jing Mao, Jun Zhao, et al. *Polymers*; published by MDPI, 2019).

**Figure 6:**
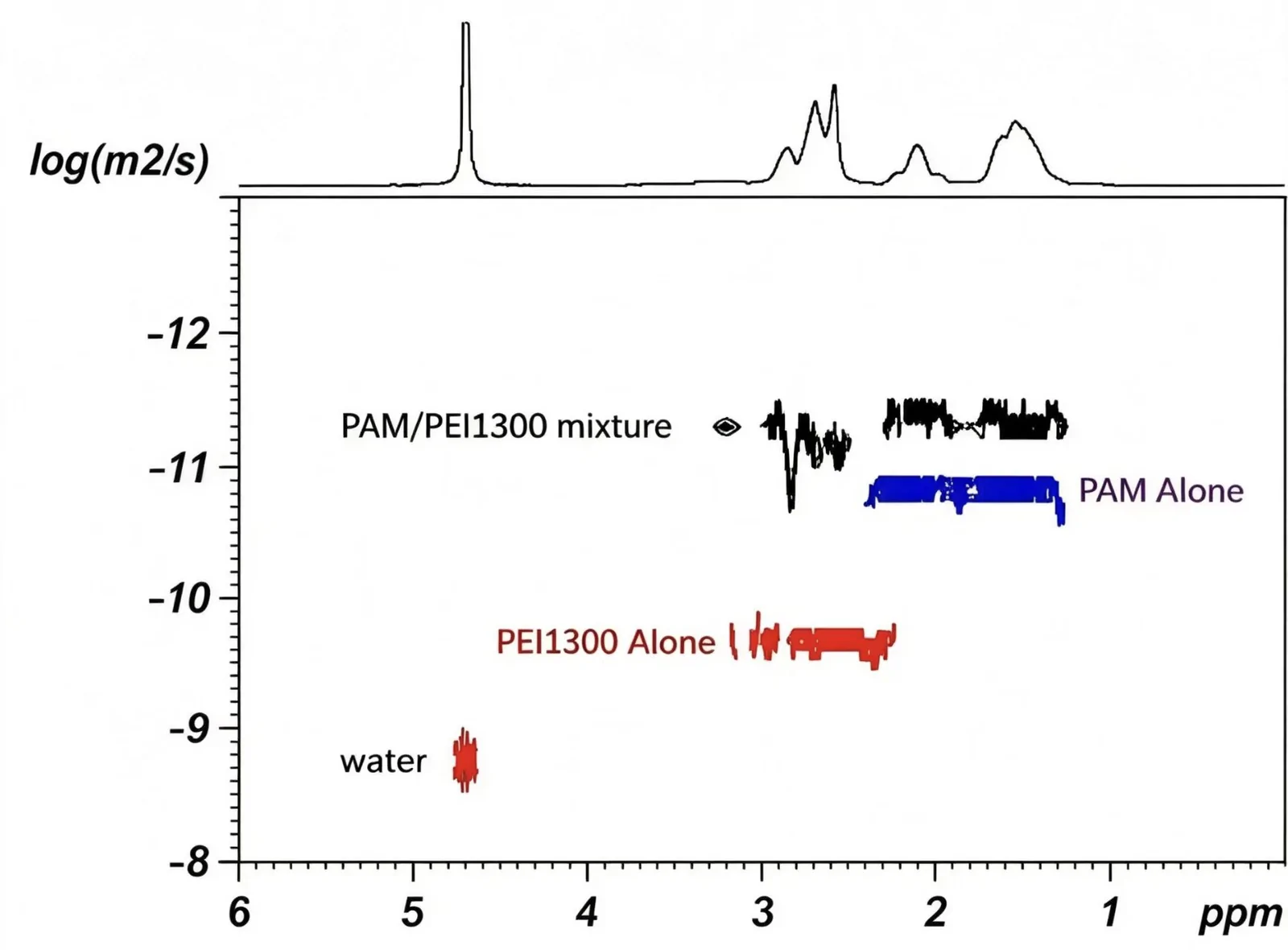
NMR DOSY spectra at 25 °C of PAM/PEI1300 [44] (Reproduced with permission from M. A. Ghriga, A. Khoukh, S. E. I. Lebouachera, et al. *Soft Matter*; published by the Royal Society of Chemistry, 2022).

**Figure 7:**
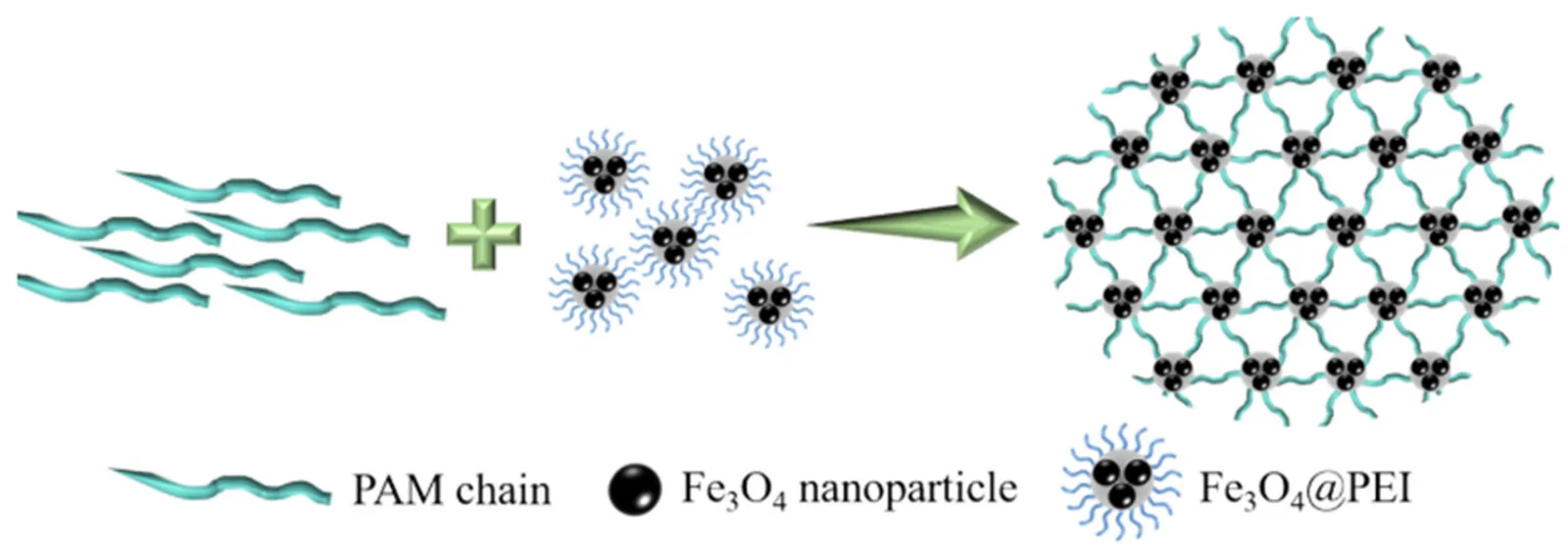
Schematic diagram of the reaction mechanism between polyacrylamide and Fe_3_O_4_@PEI [40] (Reproduced with permission from Kai Wang, Yu Guo, Jian Wen, et al. *Journal of Molecular Liquids*; published by Elsevier, 2024).

**Figure 8:**
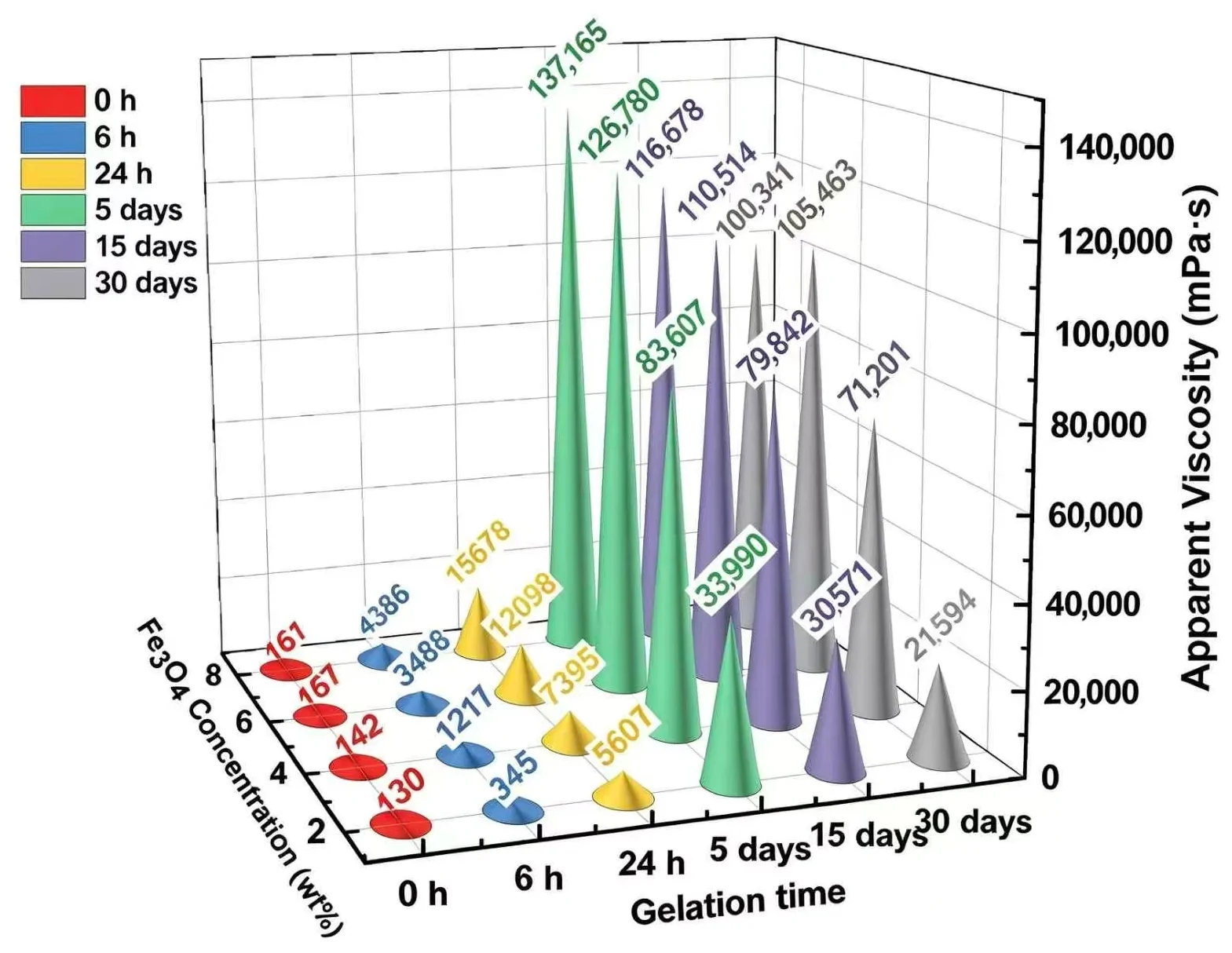
Effect of Fe_3_O_4_@PEI concentration on the apparent viscosity of magnetic intelligent polymer gels at different gelation times [40] (Reproduced with permission from Kai Wang, Yu Guo, Jian Wen, et al. *Journal of Molecular Liquids*; published by Elsevier, 2024).

**Figure 9:**
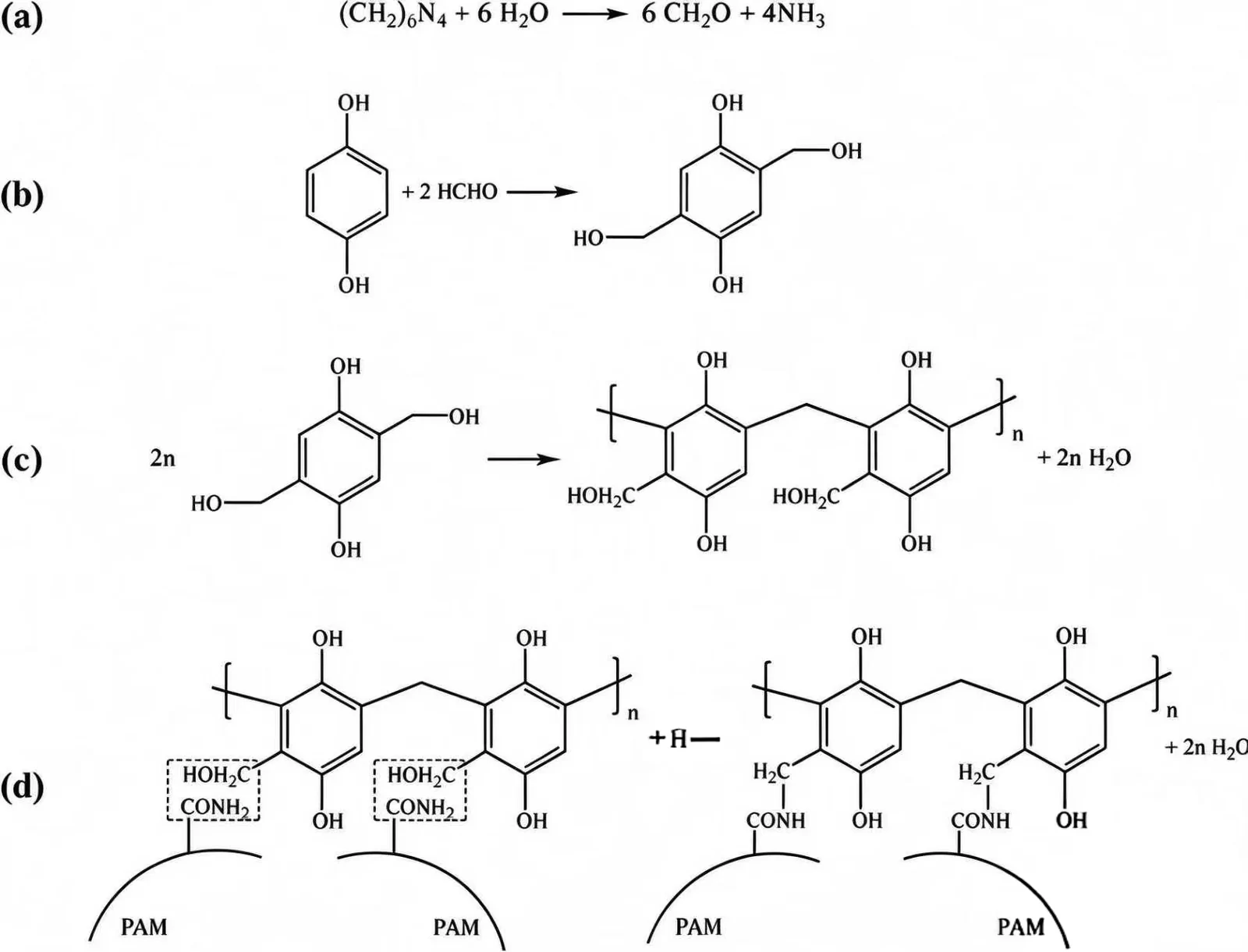
Schematic diagram of the gelation mechanism of hexamethylenetetramine (HMTA) and hydroquinone (HQ) as crosslinkers. (**a**) Chemical equation of HMTA decomposition reaction. (**b**) Chemical equation of the formation of 2,5-dihydroxymethyl hydroquinone. (c) Chemical equation of the polycondensation reaction. (**d**) Chemical equation of the crosslinking process. [38] (Reproduced with permission from Ying Liu, Chunming Dai, Kaipu Wang, et al. *Journal of Industrial and Engineering Chemistry*; published by Elsevier, 2016).

**Figure 10:**
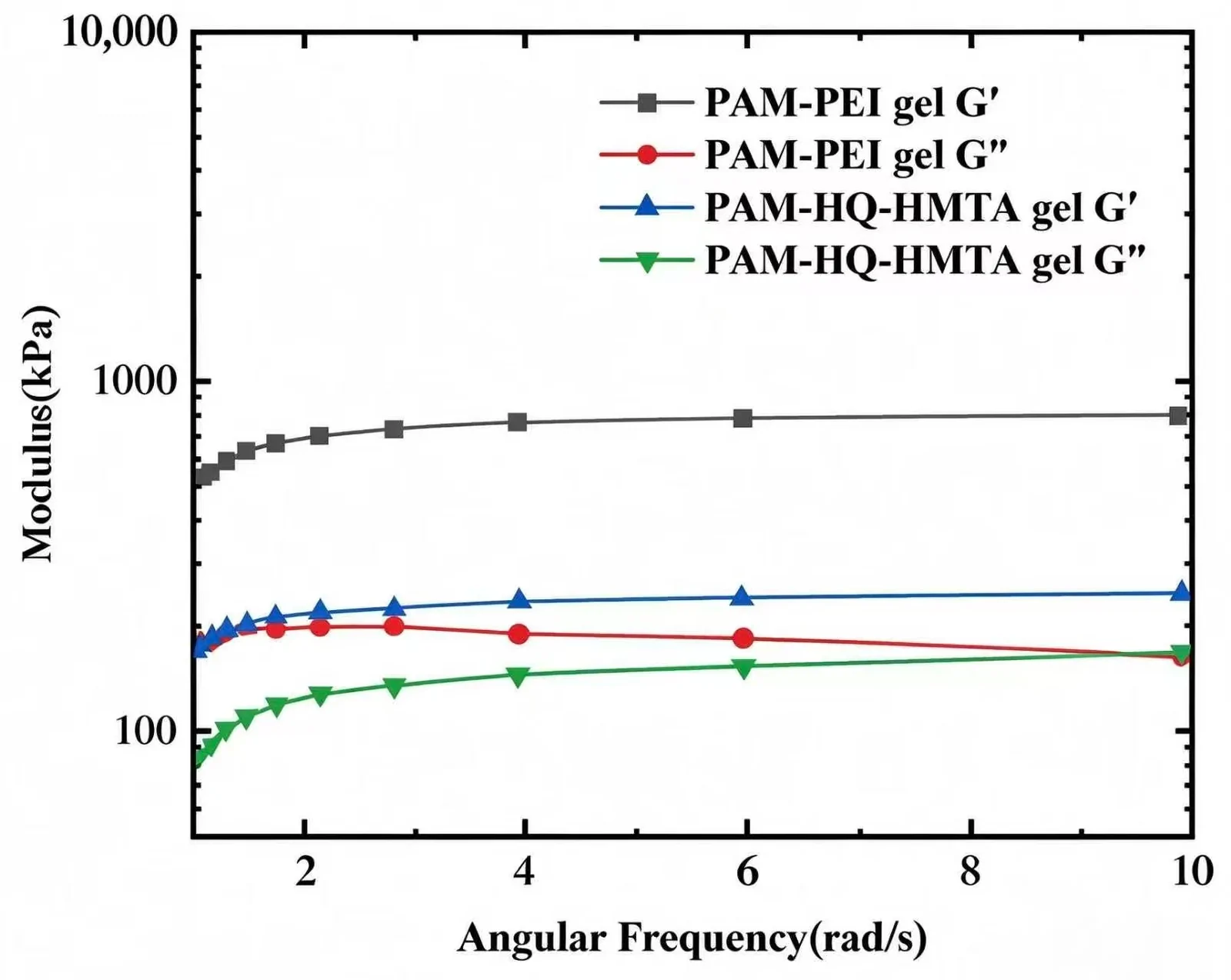
Storage (G′) and loss (G″) moduli of gel samples crosslinked with PEI and HQ-HMTA [47] (Reproduced with permission from Wen Qiao, Guang Zhang, Jun Li, et al. *Gels*; published by MDPI, 2022).

**Figure 11:**
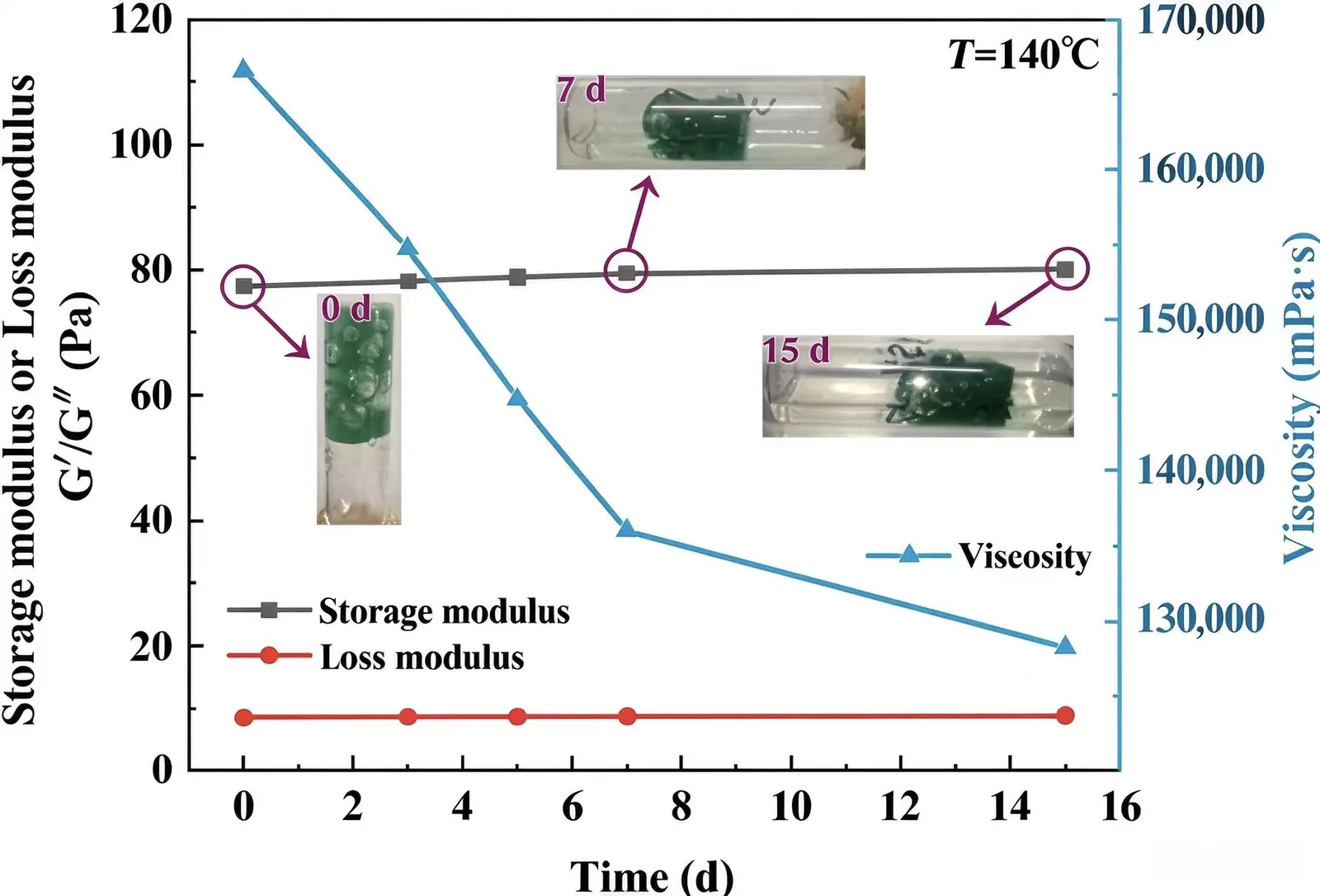
Gelation performance of the Cr^3^gel versus time. The gelation performance includes G′, G″, and viscosity. The polyacrylamide content was 1%, and the chromium acetate content was 0.2%. The photograph of the gels was taken after they had been heated in the oven at 140 °C [54] (Reproduced with permission from Guang Zhang, Lina Chen, Jian Ge, et al. *Colloids and Surfaces A: Physicochemical and Engineering Aspects*; published by Elsevier, 2015).

**Figure 12:**
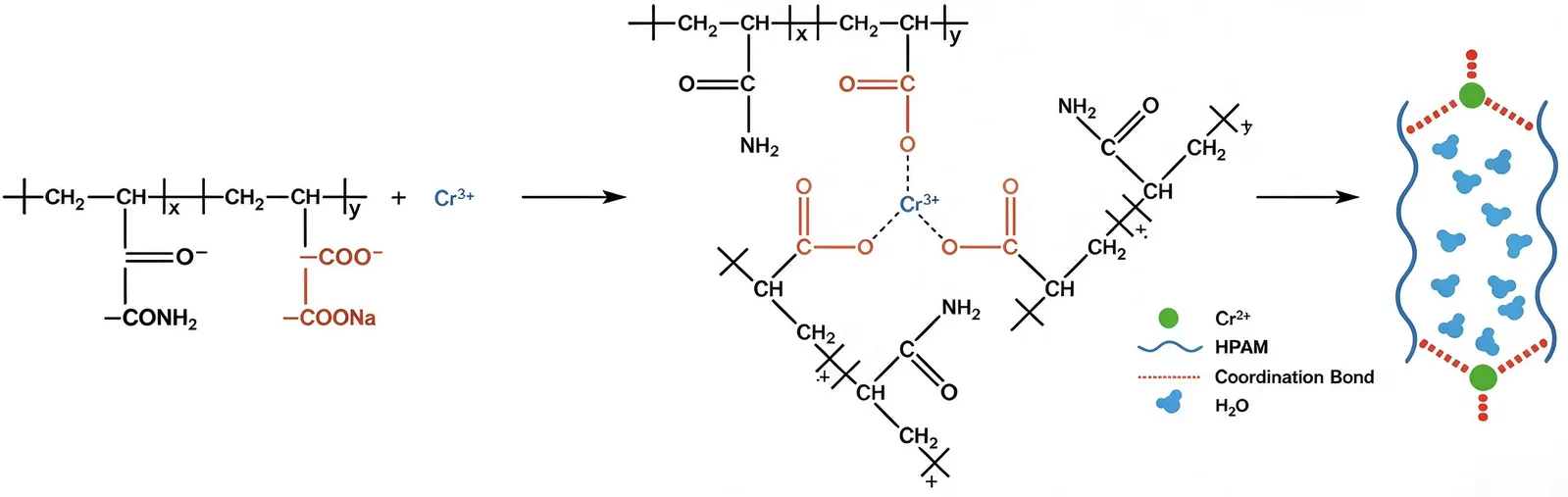
Crosslinker mechanism of Cr^3+^ [55] (Reproduced with permission from Behrouz Shahmohammadi, Mohammad Hossein Akhlaghi, Hamid Rastegar Moghadam, et al. *Nanoscale and Advanced Materials*; published by Avens Publishing Group, 2025).

**Figure 13:**
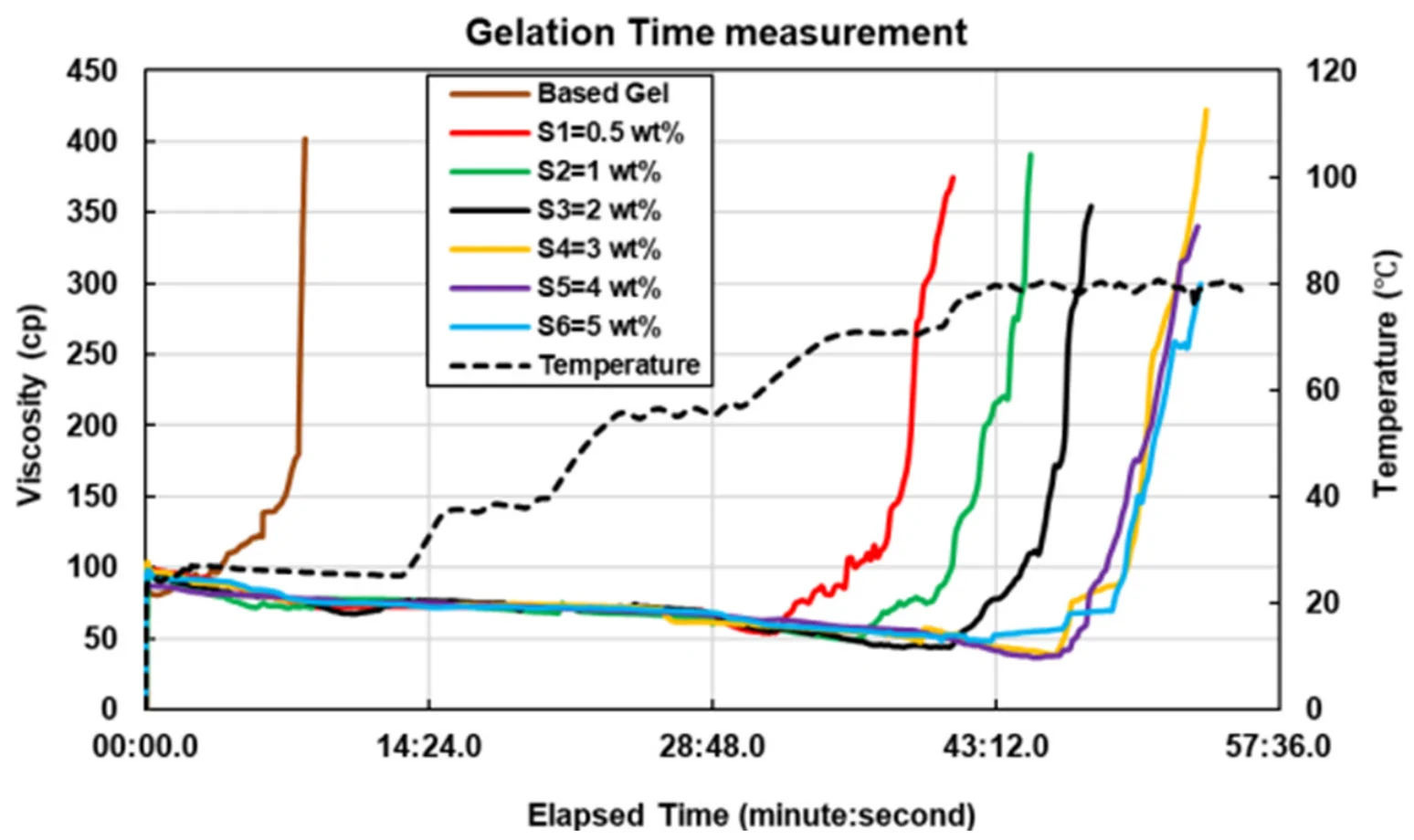
Effect of different concentrations of an industrial retarder on gelation time [56] (Reproduced with permission from Tao Song, Baojun Bai, Y. Eriyagama, et al. *ACS Applied Materials & Interfaces*; published by American Chemical Society, 2023).

**Figure 14:**
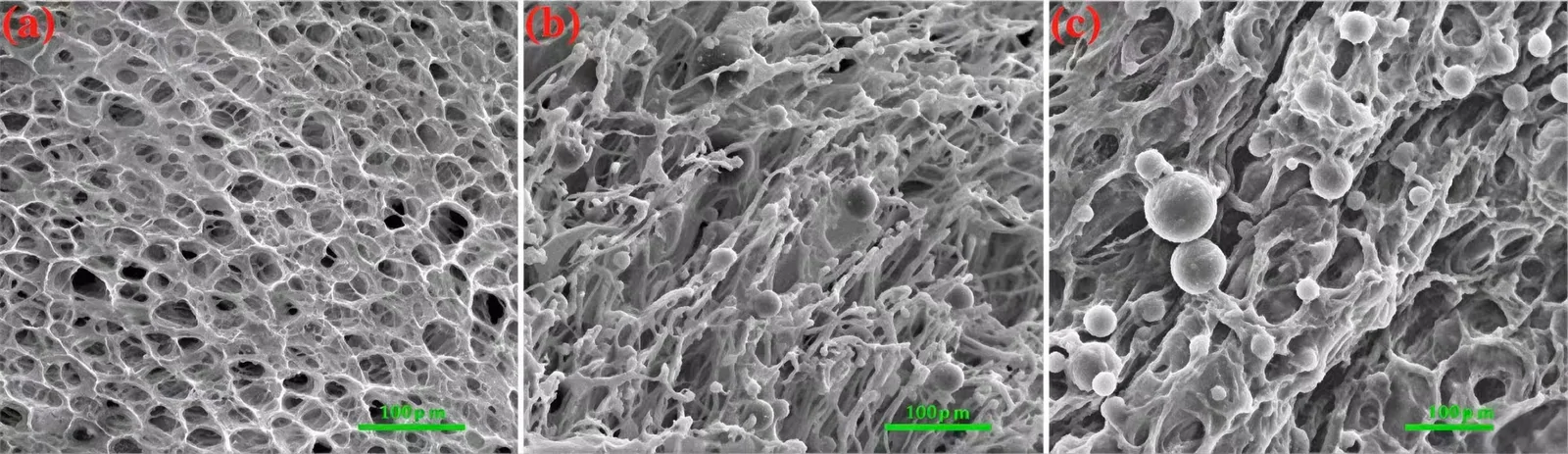
Microstructures of gels formed by different crosslinker types: (**a**) 0.2% chromium acetate, (**b**) 2% phenolic resin, and (**c**) 0.2% chromium acetate and 2% phenolic resin [54] (Reproduced with permission from Guang Zhang, Lina Chen, Jian Ge, et al. *Colloids and Surfaces A: Physicochemical and Engineering Aspects*; published by Elsevier, 2015).

**Figure 15:**
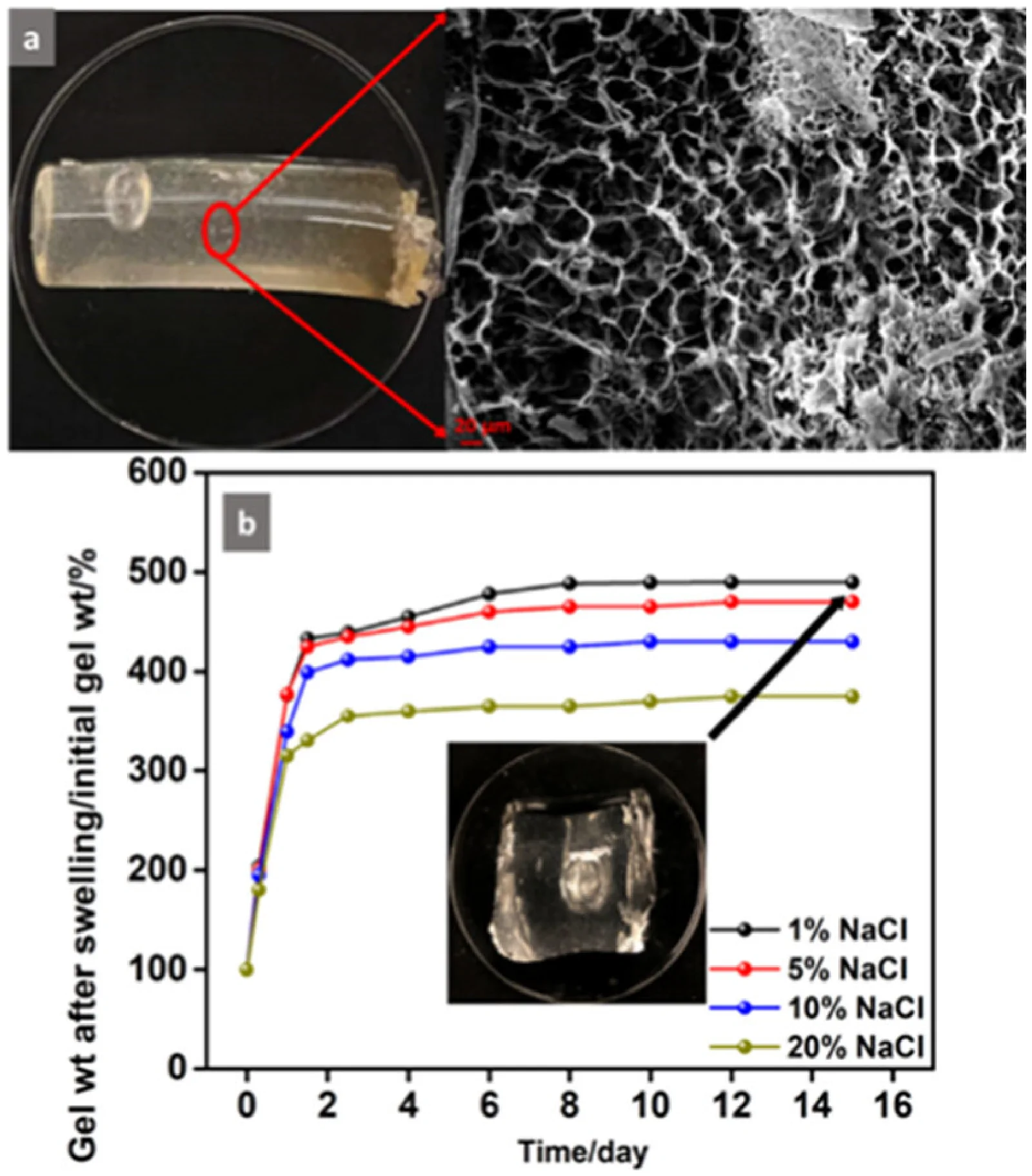
Microstructure and bulk swelling characteristics of crosslinked gel (**a**) Microstructure of the crosslinked gel and (**b**) swelling behavior of the crosslinked bulk gel [56] (Reproduced with permission from Tao Song, Baojun Bai, Y. Eriyagama, et al. *ACS Applied Materials & Interfaces*; published by American Chemical Society, 2023).

**Figure 16:**
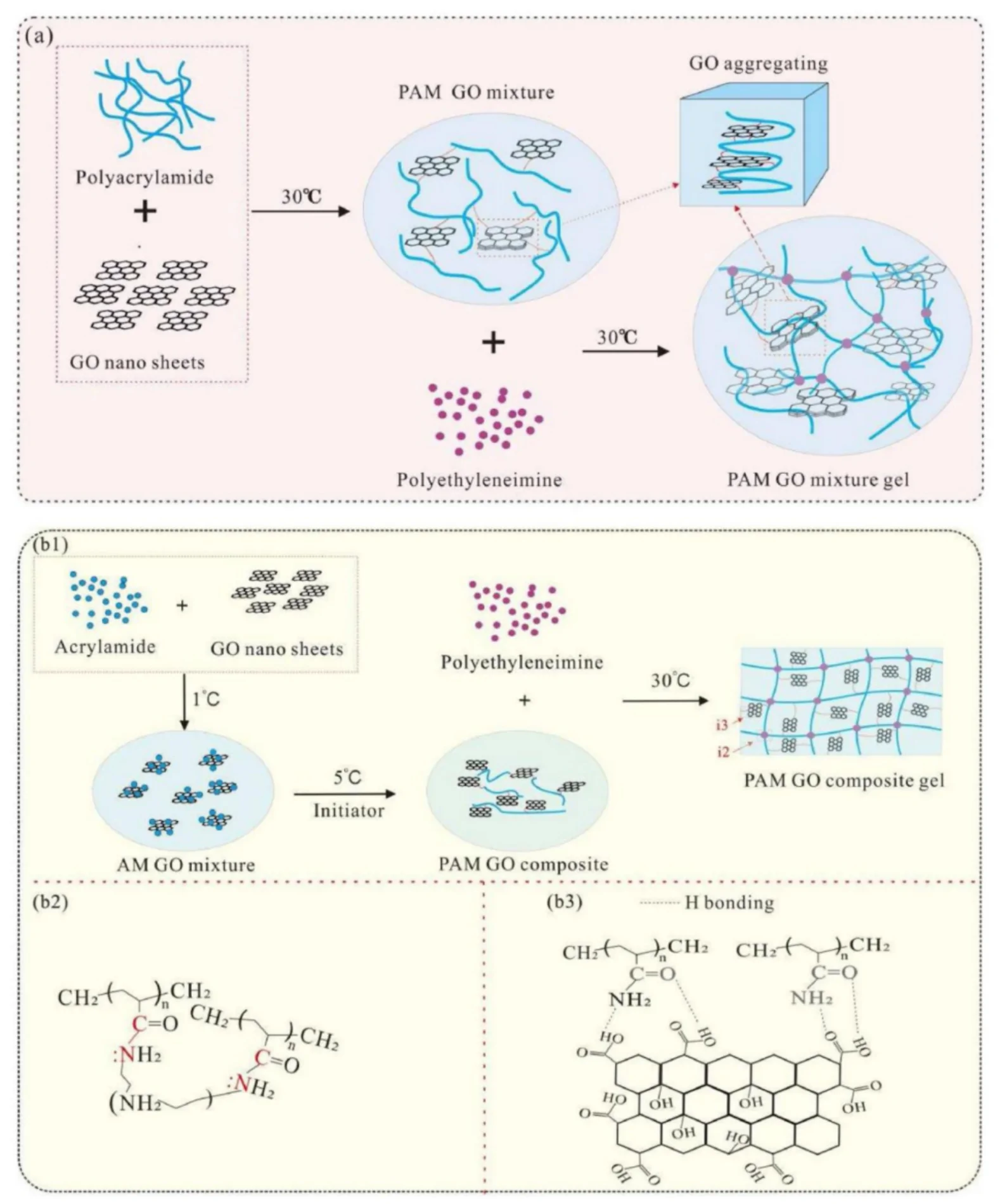
Proposed interaction of graphene oxide in the mixture gel (**a**) and composite gel (**b1**); crosslinker mechanism for PAM and PEI (**b2**) and H-bonding types between GO and PAM polymer chains in composite gel (**b3**) [33] (Reproduced with permission from Dong Qin, Wei Gao, Shuai Zhang, et al. *Geoenergy Science and Engineering*; published by Elsevier, 2023).

**Figure 17:**
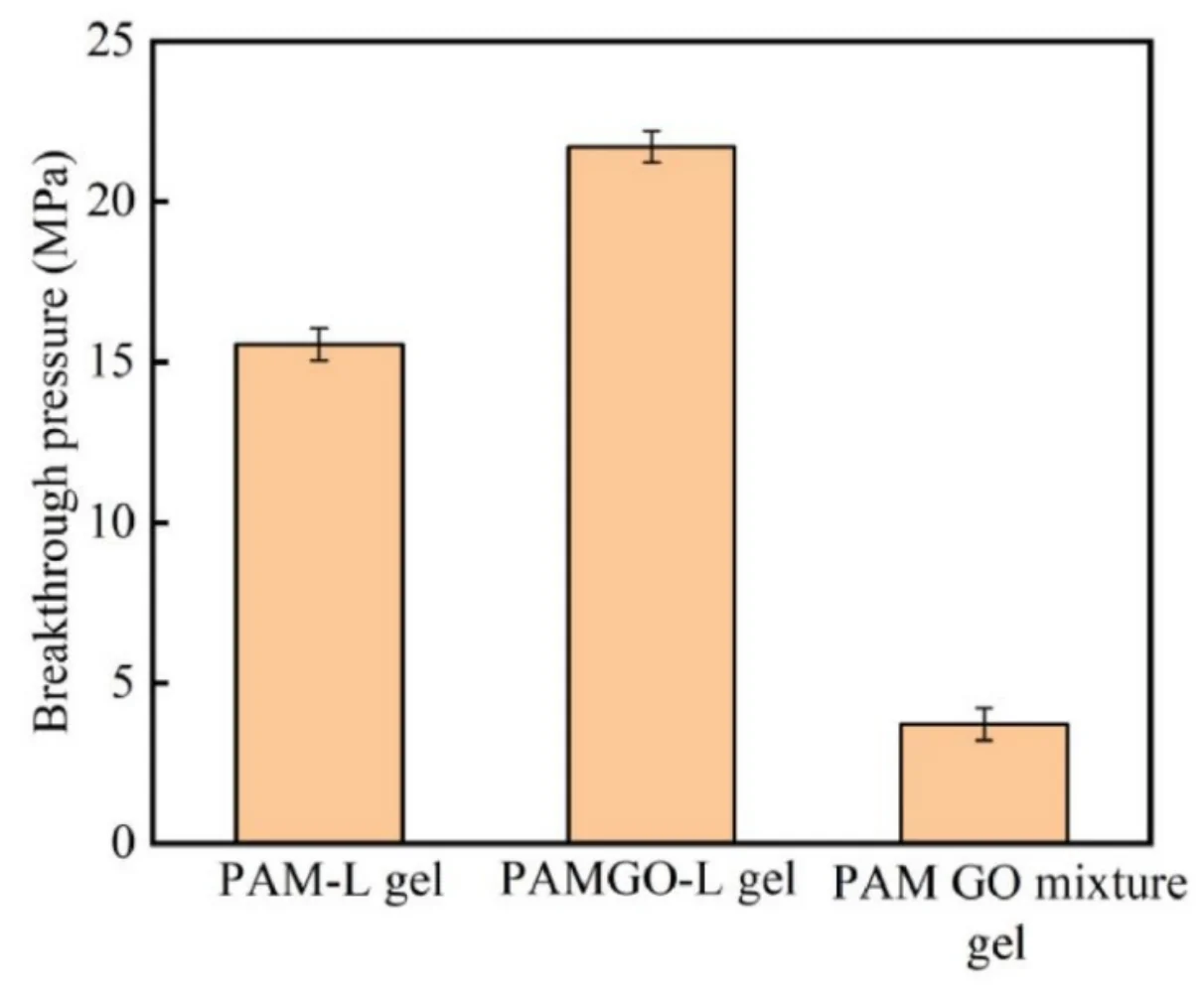
Gel breakthrough pressure test [29] (Reproduced with permission from Xinyue Cui, Chao Wang, Wen Huang, et al. *Journal of Physics and Chemistry of Solids*; published by Elsevier, 2025).

**Figure 18:**
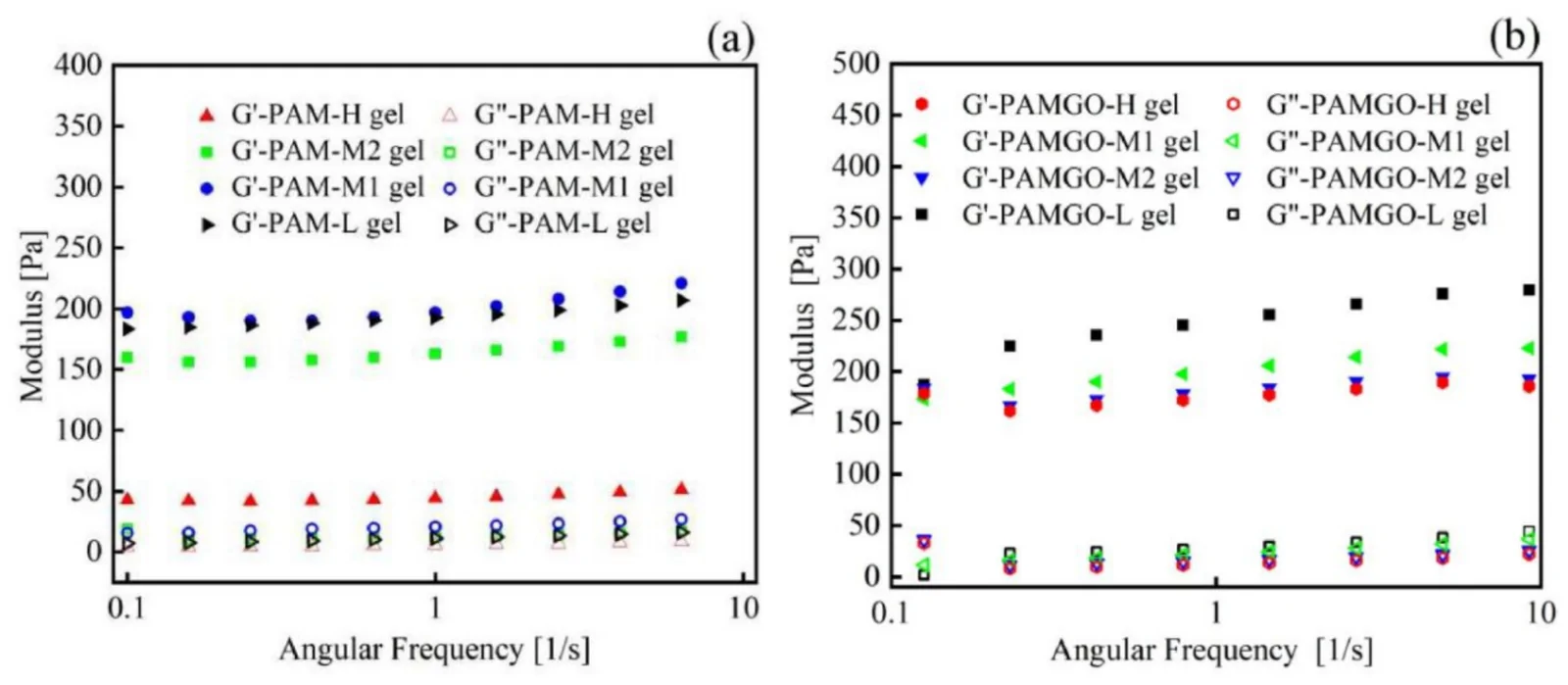
G′ and G″ of PAM (**a**) and PAMGO (**b**) [29] (Reproduced with permission from Xinyue Cui, Chao Wang, Wen Huang, et al. *Journal of Physics and Chemistry of Solids*; published by Elsevier, 2025).

**Figure 19:**
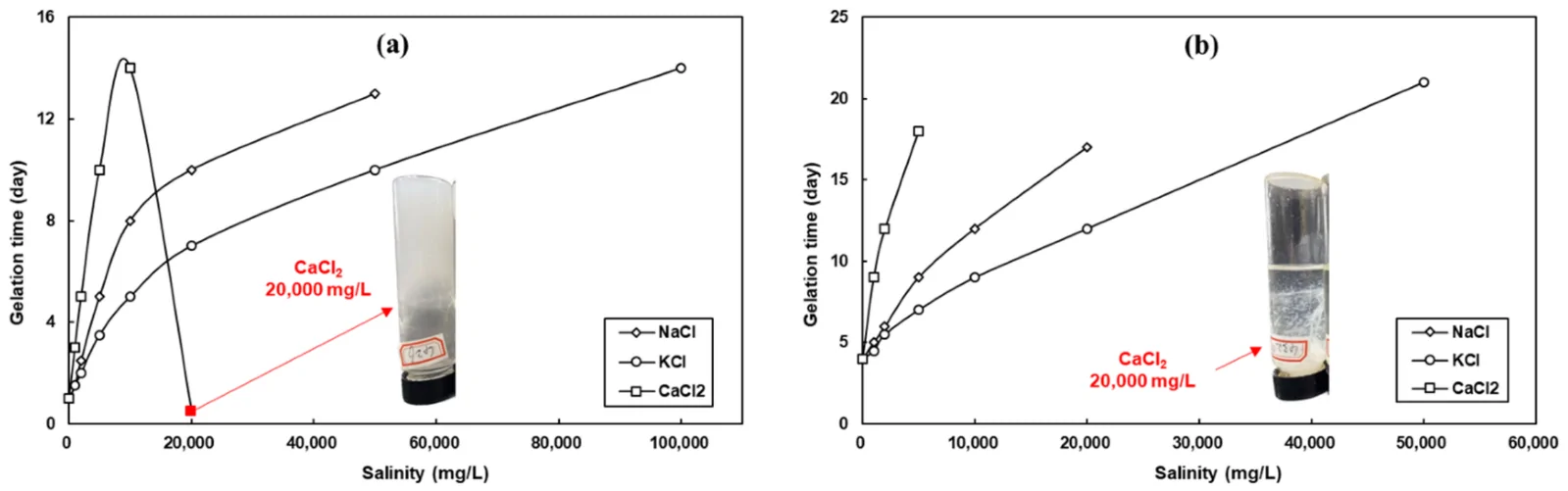
Effect of individual salt concentrations on gelation time: (**a**) 1.0 wt.% HPAM + 0.4 wt.% PEI; (**b**) 0.4 wt.% HPAM + 0.16 wt.% PEI [69] (Reproduced with permission from Hui Guo, Chao Xue, Dong Wang. *Processes*; published by MDPI, 2026).

**Figure 20:**
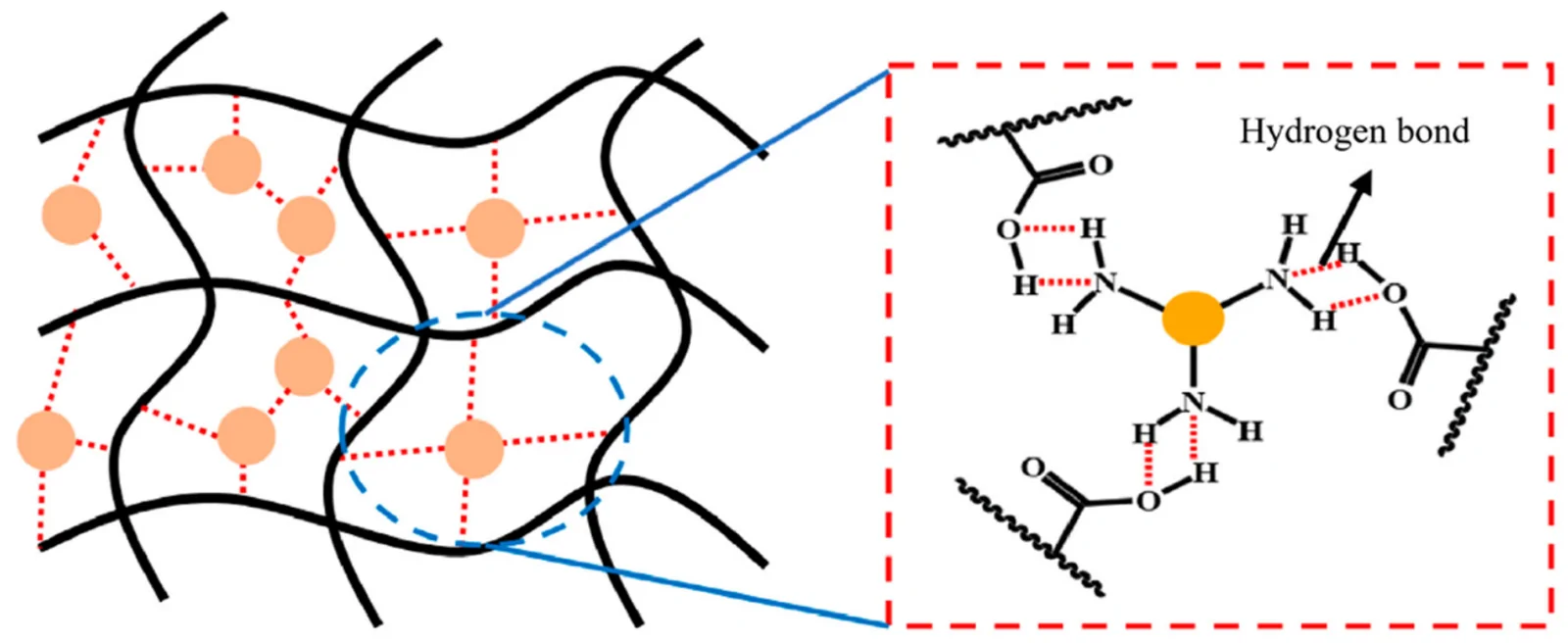
Schematic diagram of the crosslinker between HPAM and PEI [71] (Reproduced with permission from Yuguo Yu, Jianrui Hou, Huan Luo, et al. *Oilfield Chemistry (English Edition)*; published by Editorial Office of Oilfield Chemistry, 2012).

**Figure 21:**
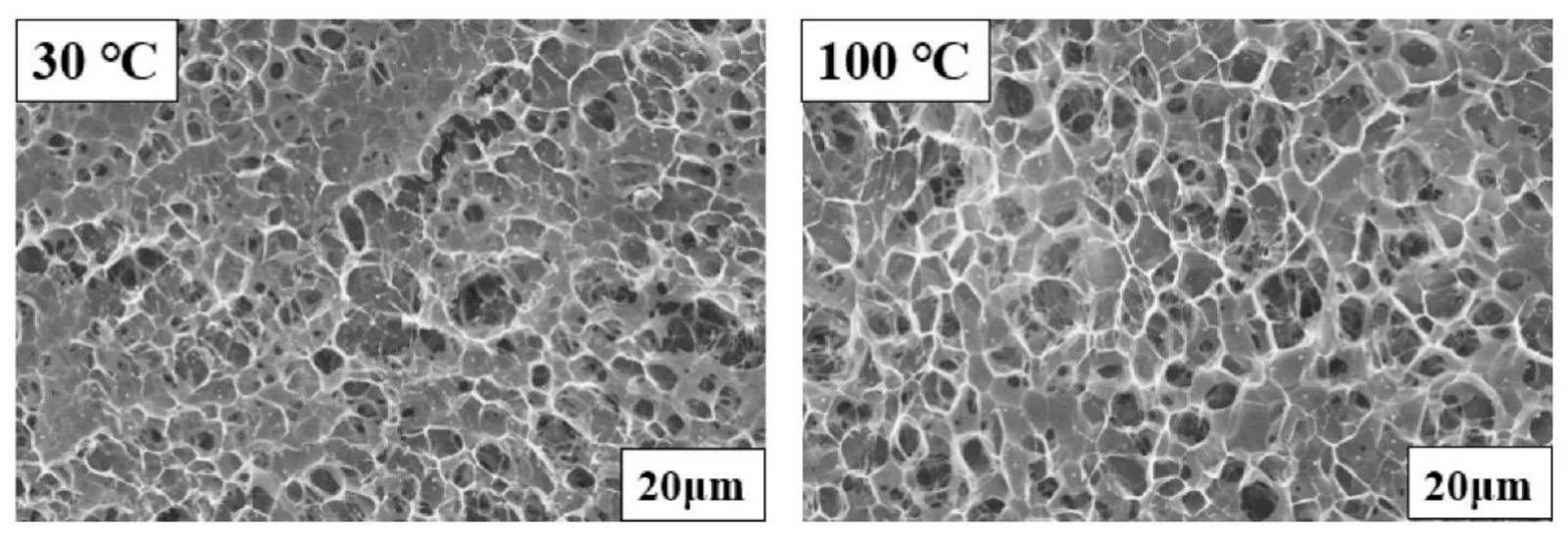
SEM images of the HPAM/PEI system after gelation at different temperatures [22] (Reproduced with permission from Jian Xu, Jie Han, Xiang Wang, et al. *Colloids and Surfaces A: Physicochemical and Engineering Aspects*; published by Elsevier, 2026).

**Figure 22:**
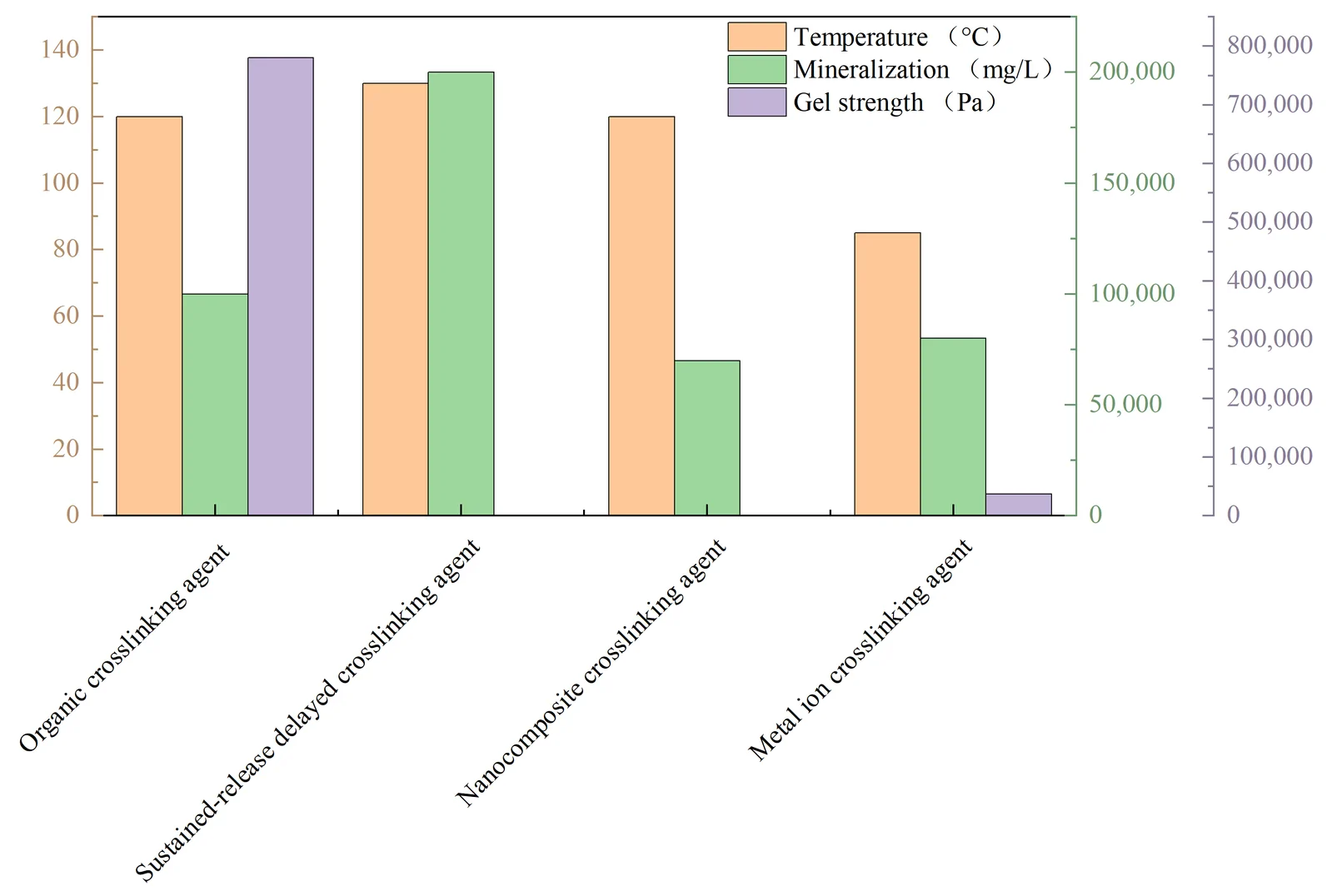
Performance comparison of different crosslinkers for PAM gel systems [25,77,78,79] (Original figure compiled by the authors of this review, with data extracted from references [25,77,78,79]. No secondary reproduction permission is required).

**Figure 24:**
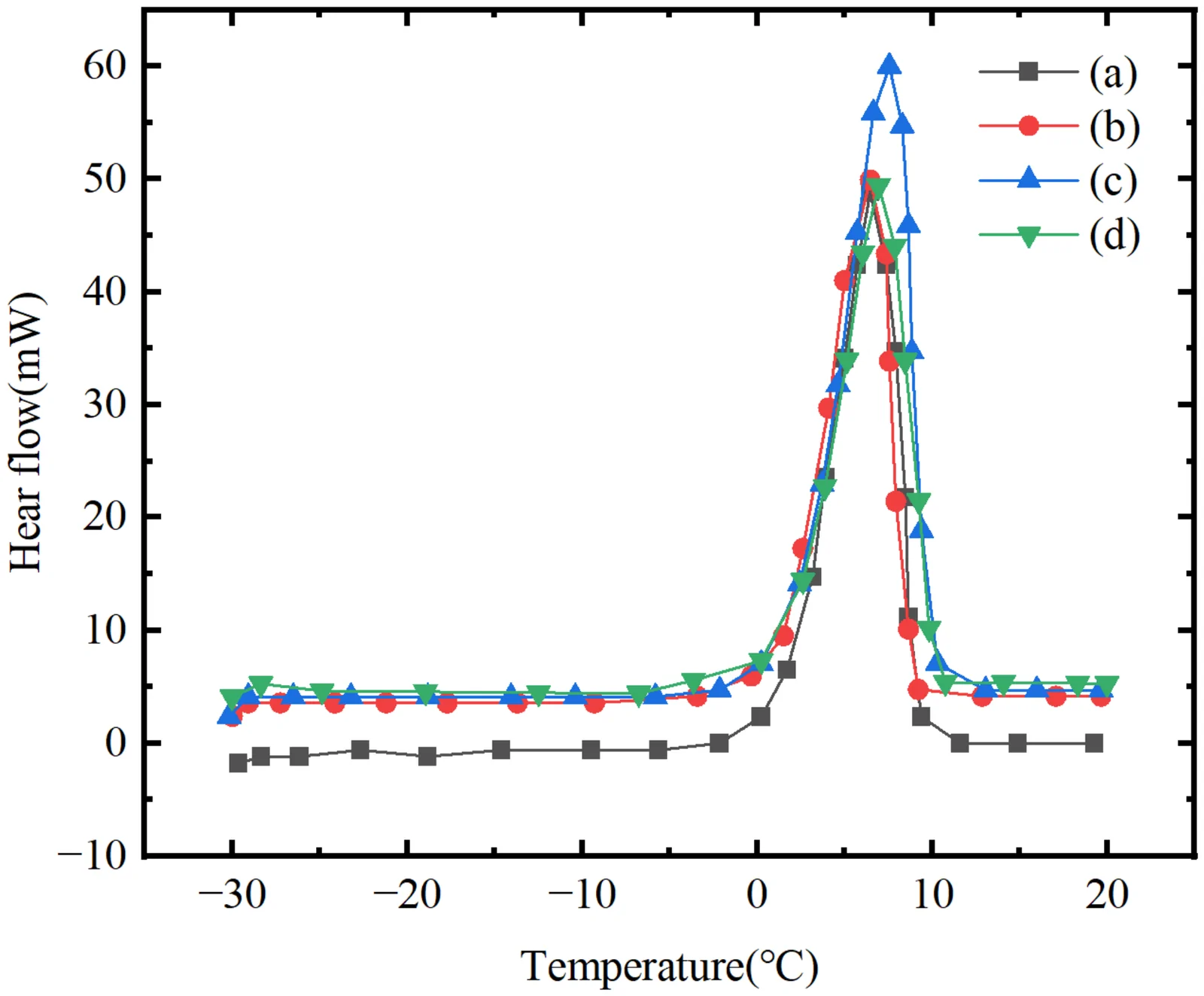
DSC curves of gel samples prepared with silica nanoparticles at different concentrations (scanning temperature range: −30 to 20 °C): (a) without silica nanoparticles; (b) containing 0.1 wt%; (c) containing 0.2 wt%; (d) containing 0.3 wt% silica nanoparticles [47] (Reproduced with permission from Wen Qiao, Guang Zhang, Jun Li, et al. *Gels*; published by MDPI, 2022).

**Figure 25:**
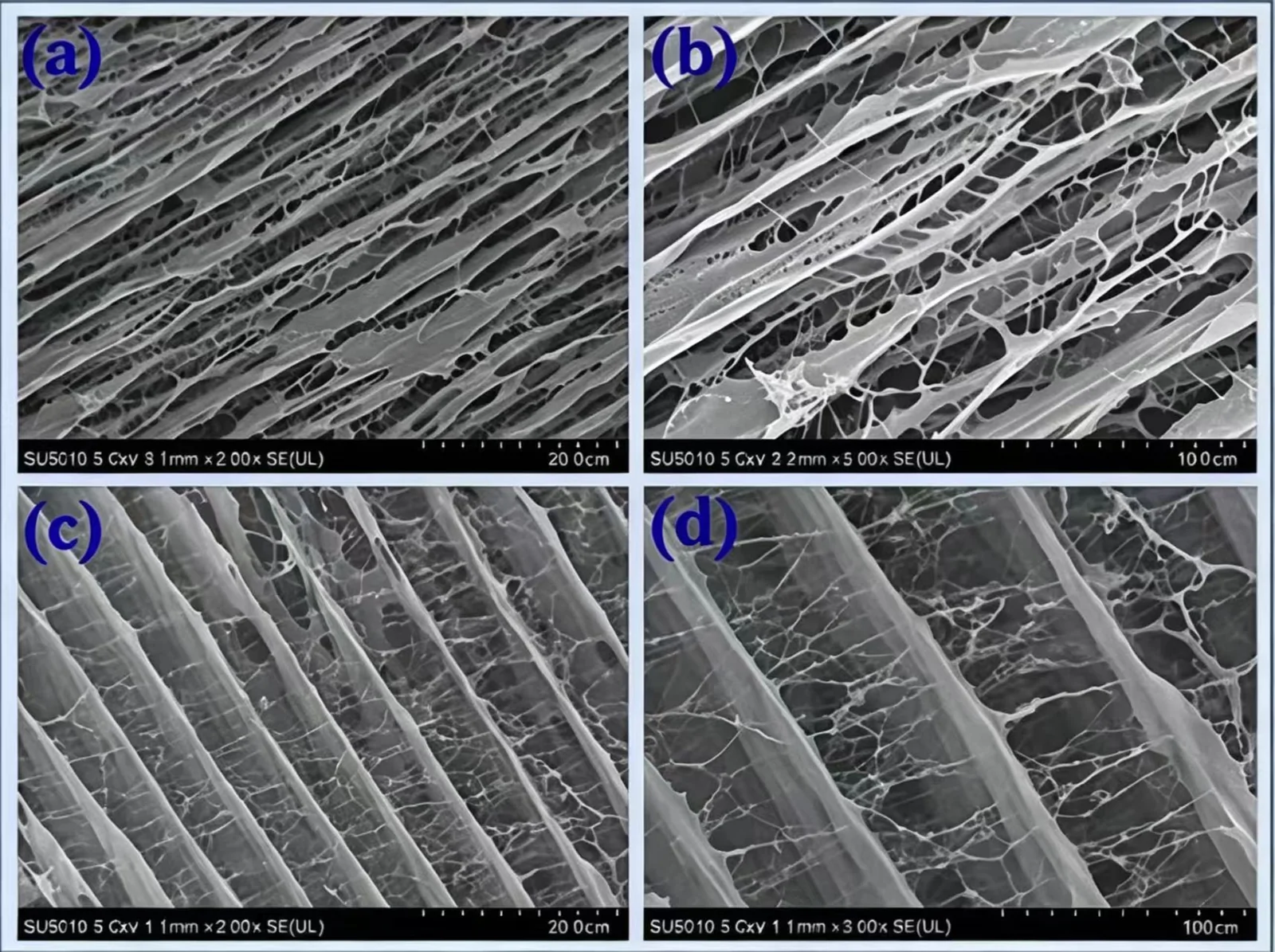
Micromorphology of gelant at different magnifications: for (**a**,**b**) gelant without nanoparticles, (**c**,**d**) gelant with 0.2 wt% nanoparticles [91] (Reproduced with permission from Qiang Wu, Jian Ge. *Journal of Molecular Liquids*; published by Elsevier, 2021).

**Figure 26:**
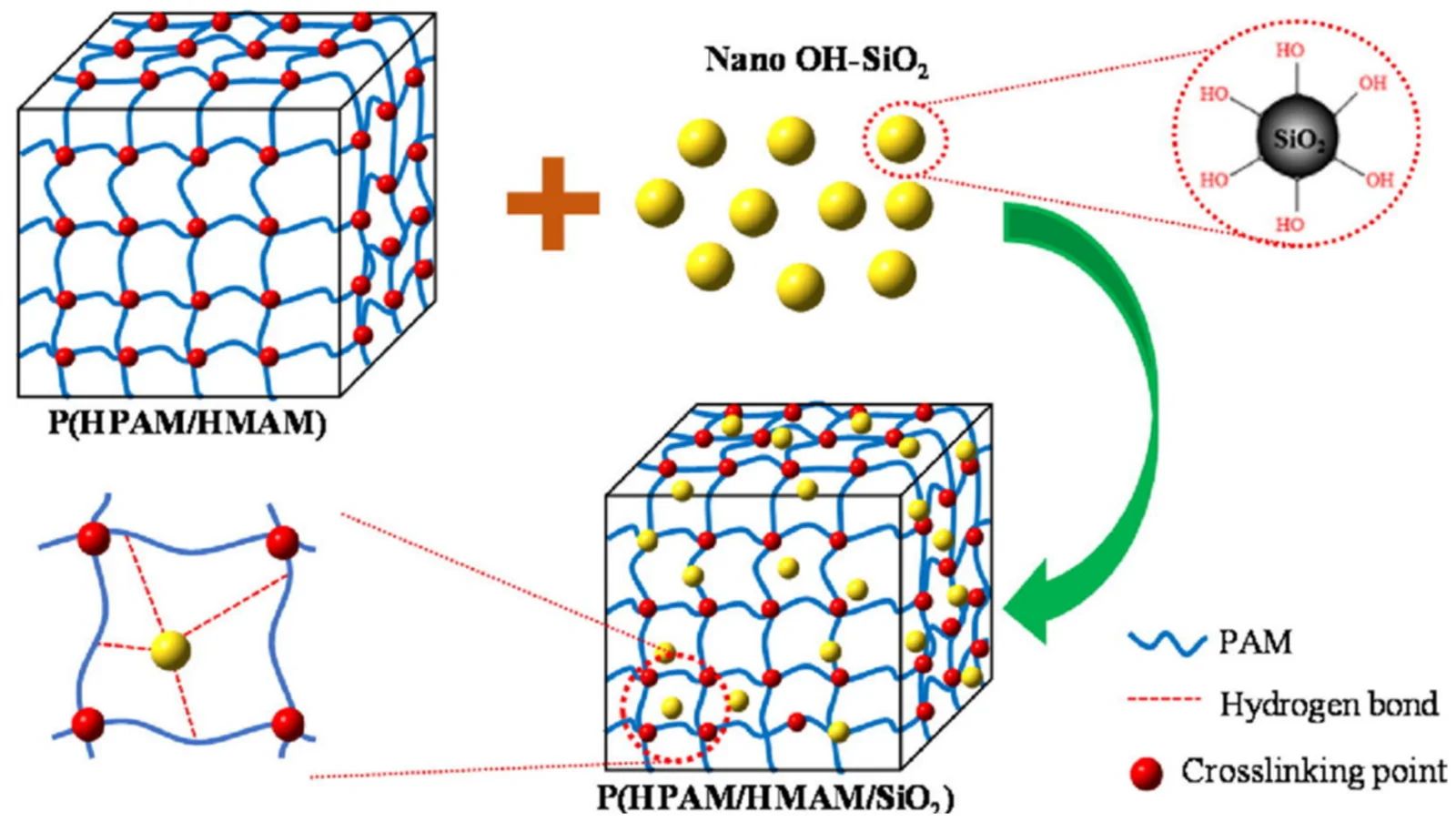
Nano OH-SiO_2_ adheres to the P (HPAM/HMAM) structure [87] (Reproduced with permission from Jian Liu, Shuo Wang, Lei He, et al. *Journal of Petroleum Science and Engineering*; published by Elsevier, 2022).

**Figure 27:**
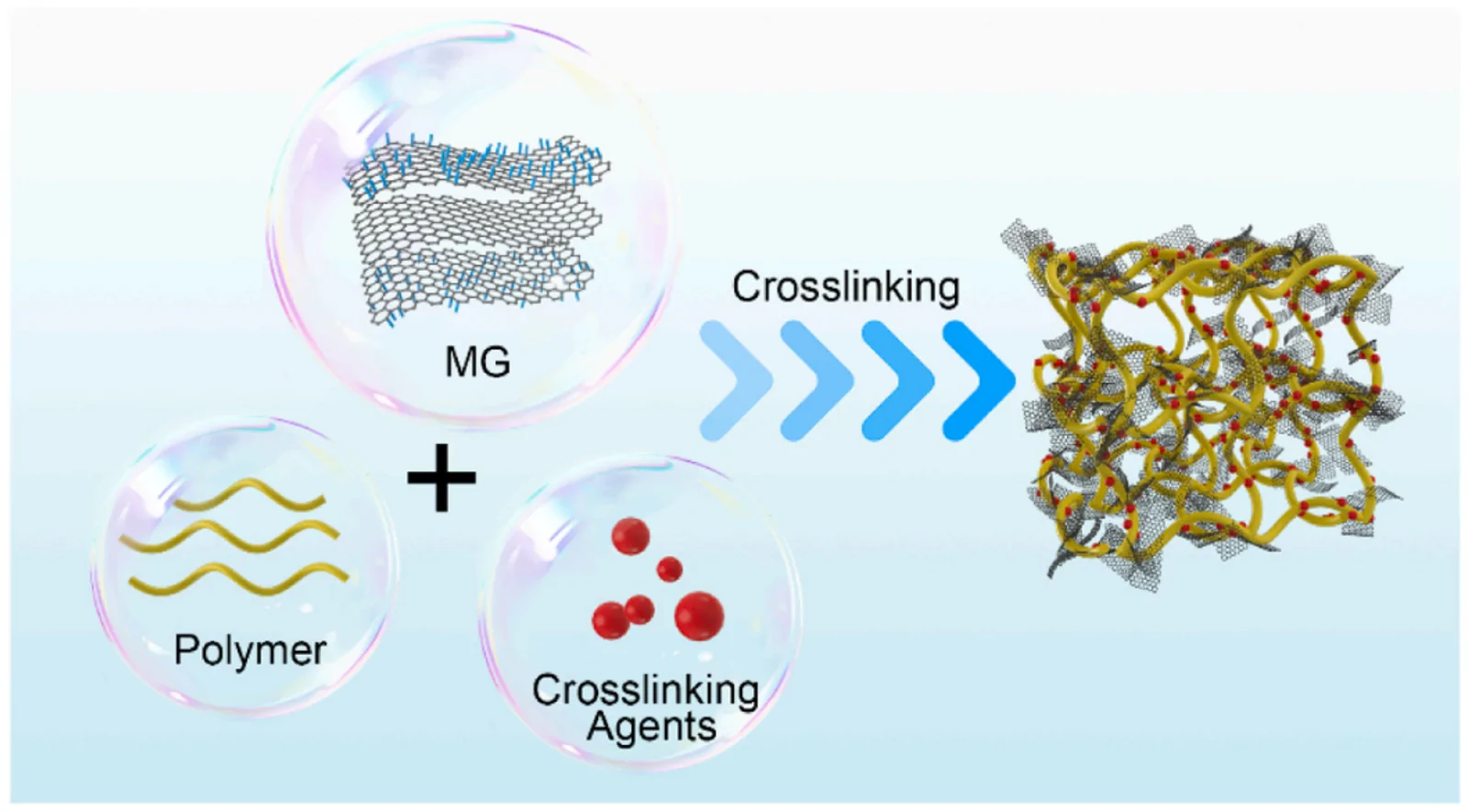
Mechanism for increasing the gel strength with the MG particles and enhancing the strength of the gel [46] (Reproduced with permission from Shuai Lu, Qiang Liu, Peng Li, et al. *Colloids and Surfaces A: Physicochemical and Engineering Aspects*; published by Elsevier, 2024).

**Figure 28:**
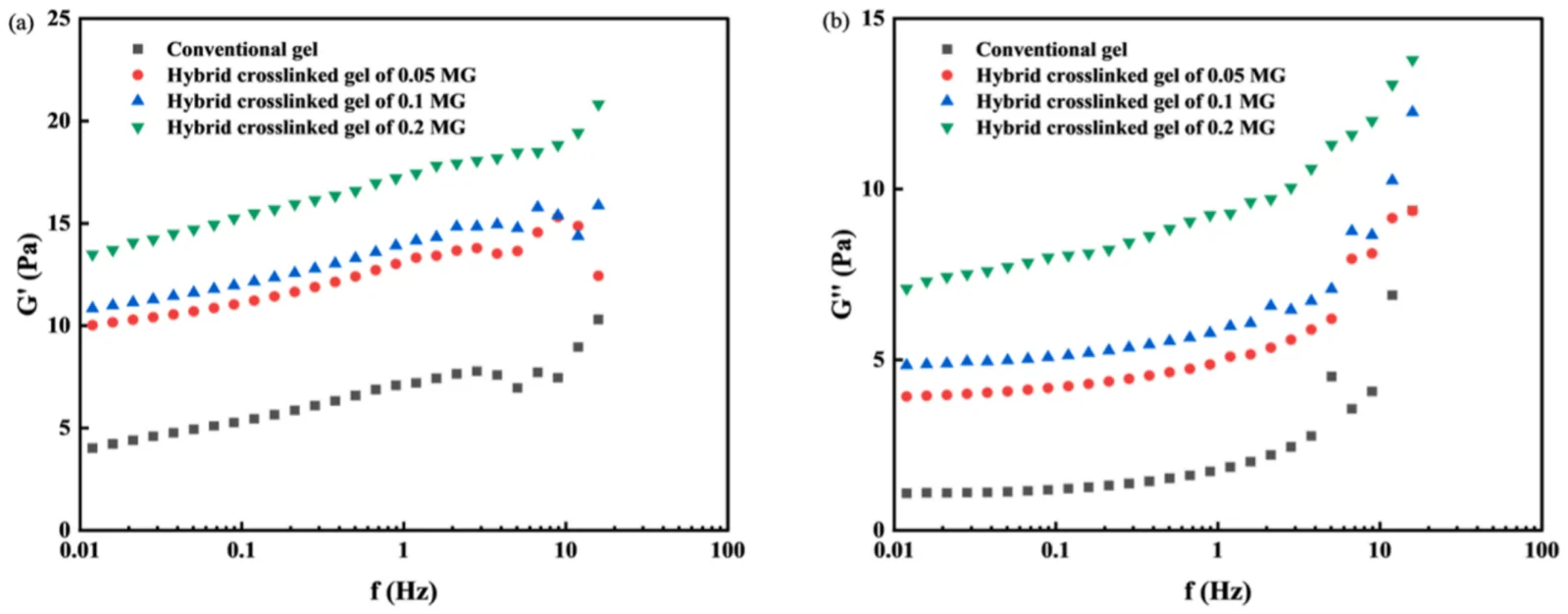
Storage moduli (G′) and loss moduli (G″) of gels with different concentrations of MG: (**a**) storage moduli (G′) and (**b**) loss moduli (G″) [46] (Reproduced with permission from Shuai Lu, Qiang Liu, Peng Li, et al. *Colloids and Surfaces A: Physicochemical and Engineering Aspects*; published by Elsevier, 2024).

**Figure 29:**
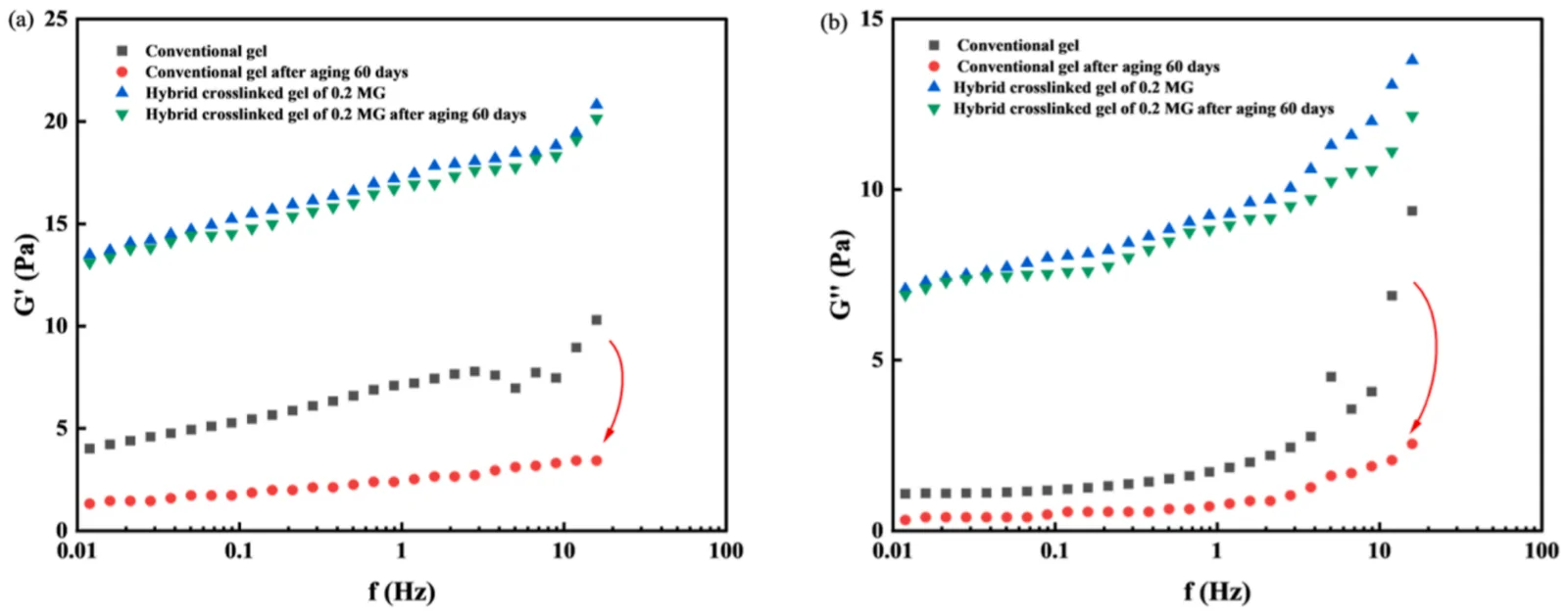
Storage moduli (G′) and loss moduli (G″) of gels with different concentrations of MG after ageing for 60 days: (**a**) storage moduli (G′) and (**b**) loss moduli (G″) [46] (Reproduced with permission from Shuai Lu, Qiang Liu, Peng Li, et al. *Colloids and Surfaces A: Physicochemical and Engineering Aspects*; published by Elsevier, 2024).

**Figure 30:**
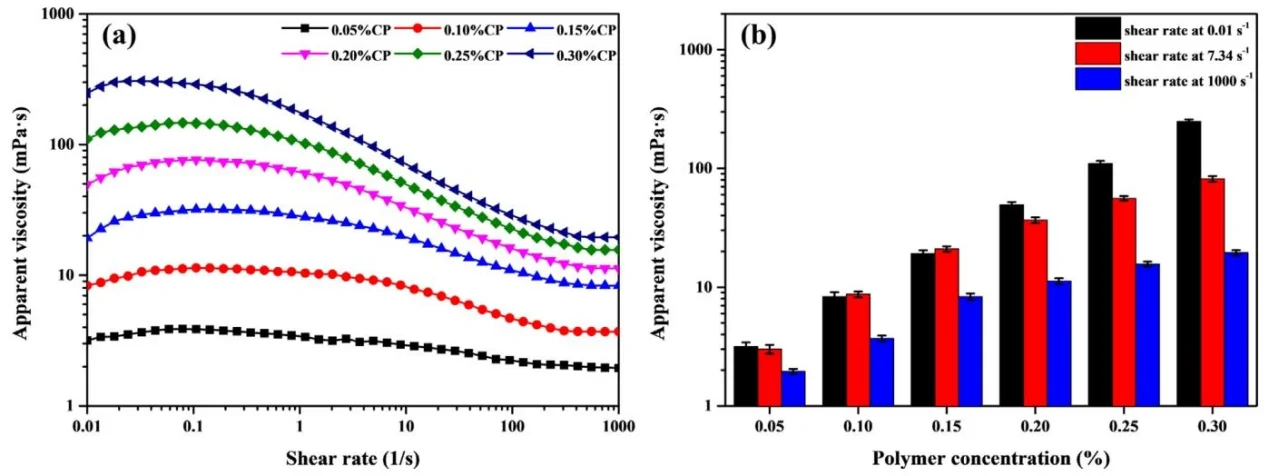
Effects of concentration on rheology and apparent viscosity: (**a**) Rheological curves of conventional polymer flooding systems at different concentrations; (**b**) Effect of polymer concentration on apparent viscosity at different shear rates [99] (Reproduced with permission from Jian Li, Guang Zhao, Nan Sun, et al. *Journal of Molecular Liquids*; published by Elsevier, 2022).

**Figure 31:**
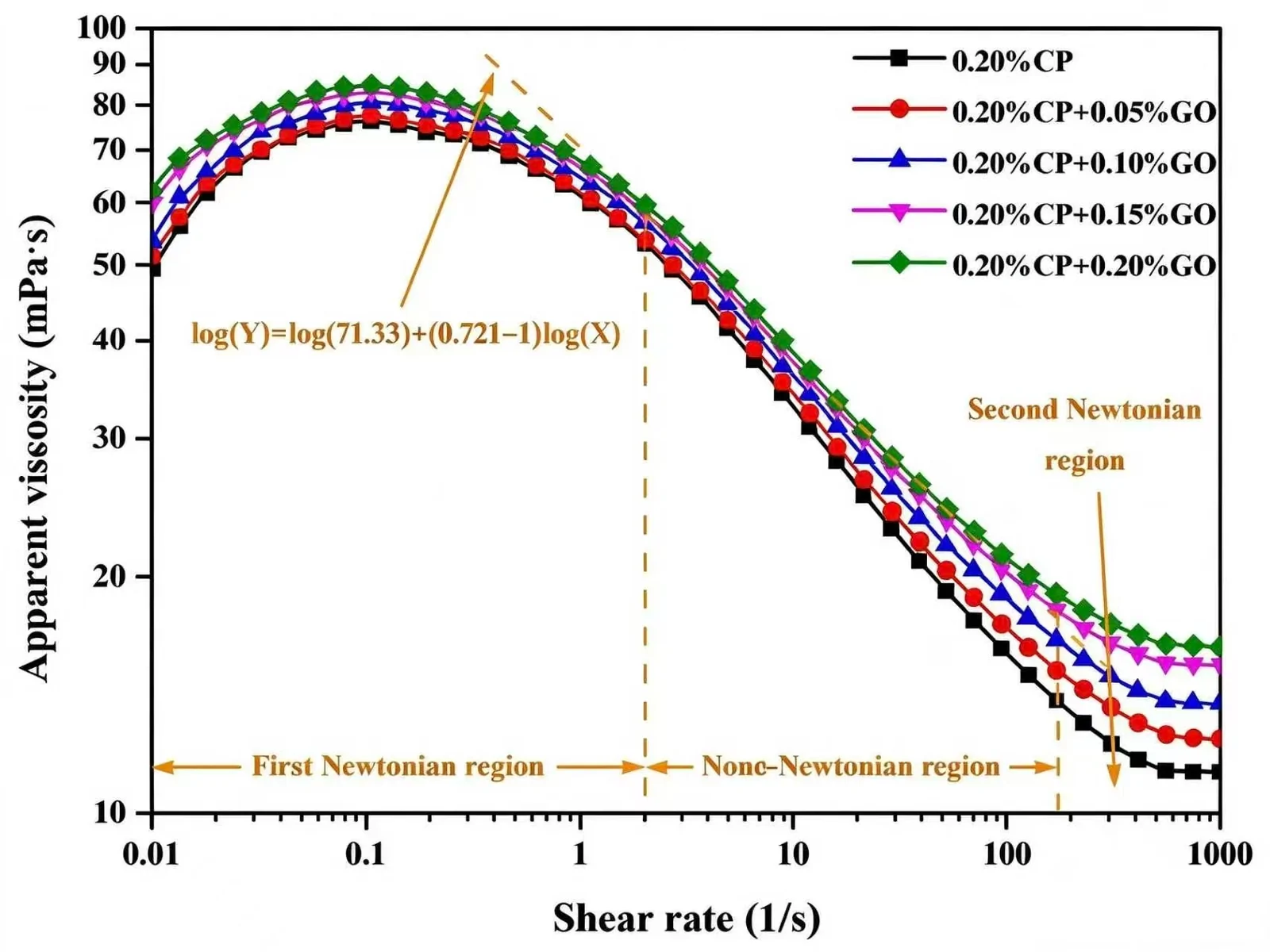
Rheological curves of polymer flooding systems reinforced with graphene oxide nanoparticles [99] (Reproduced with permission from Jian Li, Guang Zhao, Nan Sun, et al. *Journal of Molecular Liquids*; published by Elsevier, 2022).

**Figure 32:**
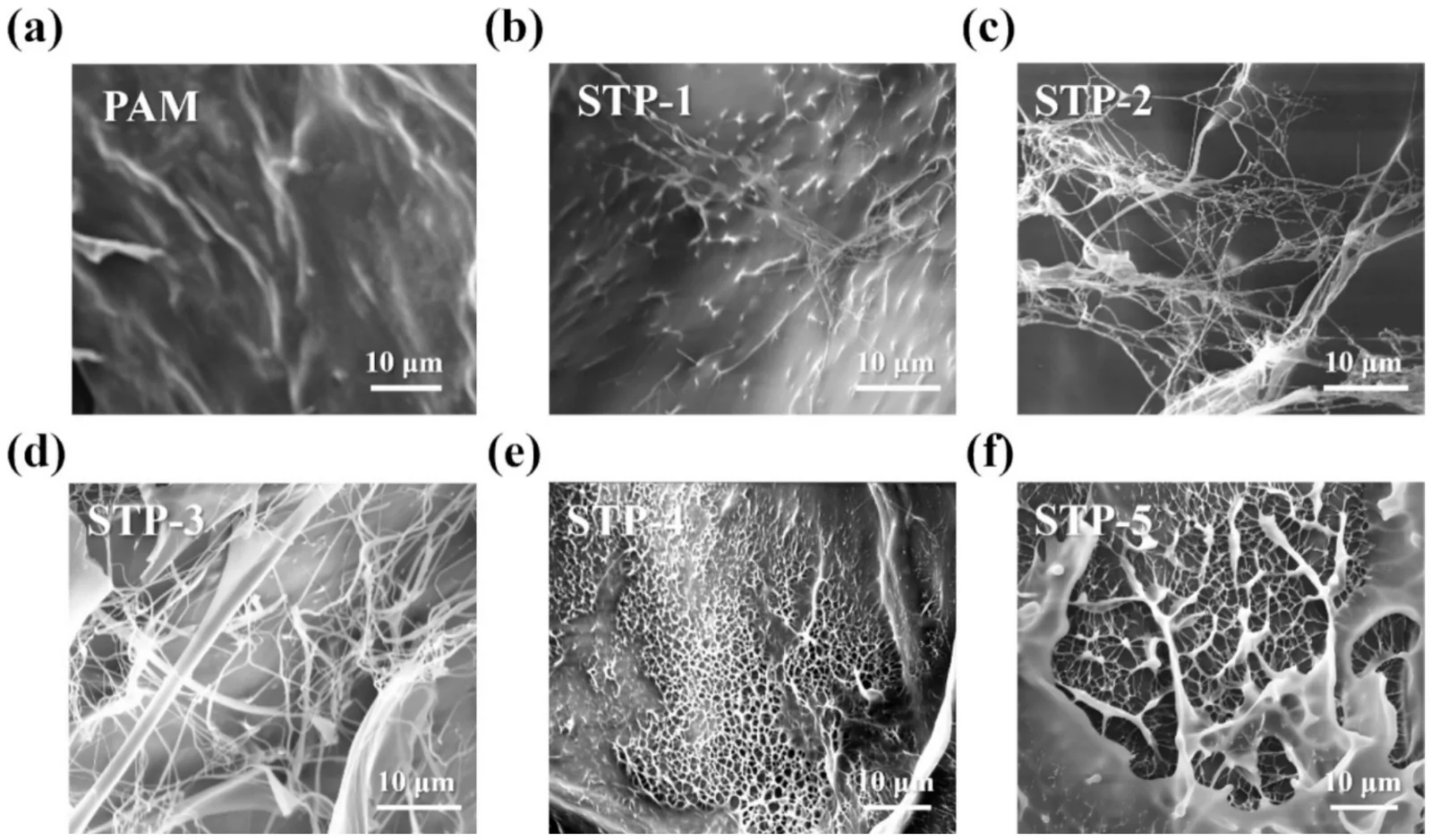
Microscopic morphology of the cross-section of PAM hydrogel and STP. (**a**) PAM, (**b**) STP-1, (**c**) STP-2, (**d**) STP-3, (**e**) STP-4 and (**f**) STP-5 [7] (Reproduced with permission from Jian Li, Guang Zhao, Nan Sun, et al. *Journal of Molecular Liquids*; published by Elsevier, 2022).

**Figure 33:**
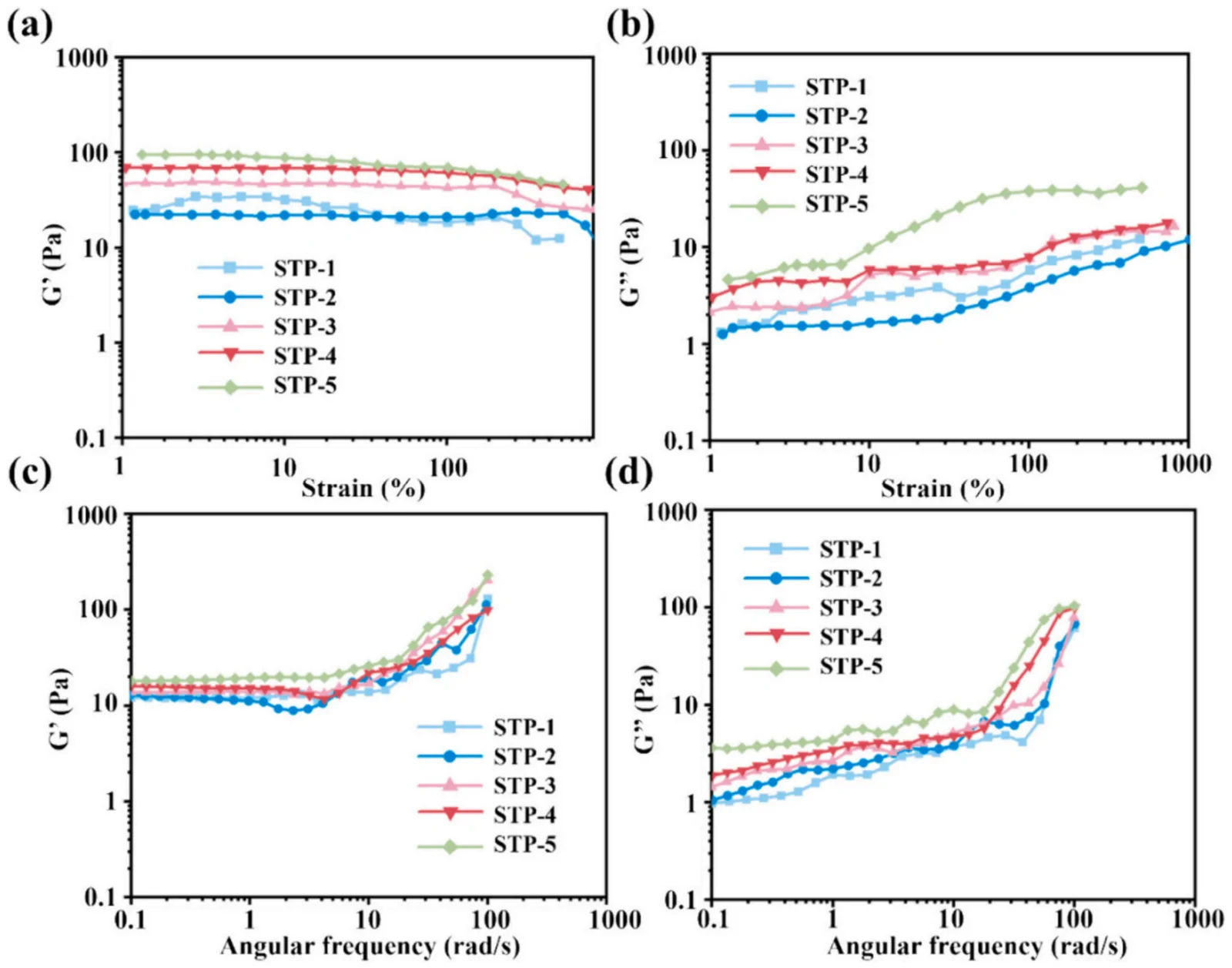
Effect of biomass components on viscoelastic properties of STPs. (**a**,**c**) Elastic, (**b**,**d**) viscous [7] (Reproduced with permission from Jian Li, Guang Zhao, Nan Sun, et al. *Journal of Molecular Liquids*; published by Elsevier, 2022).

**Figure 34:**
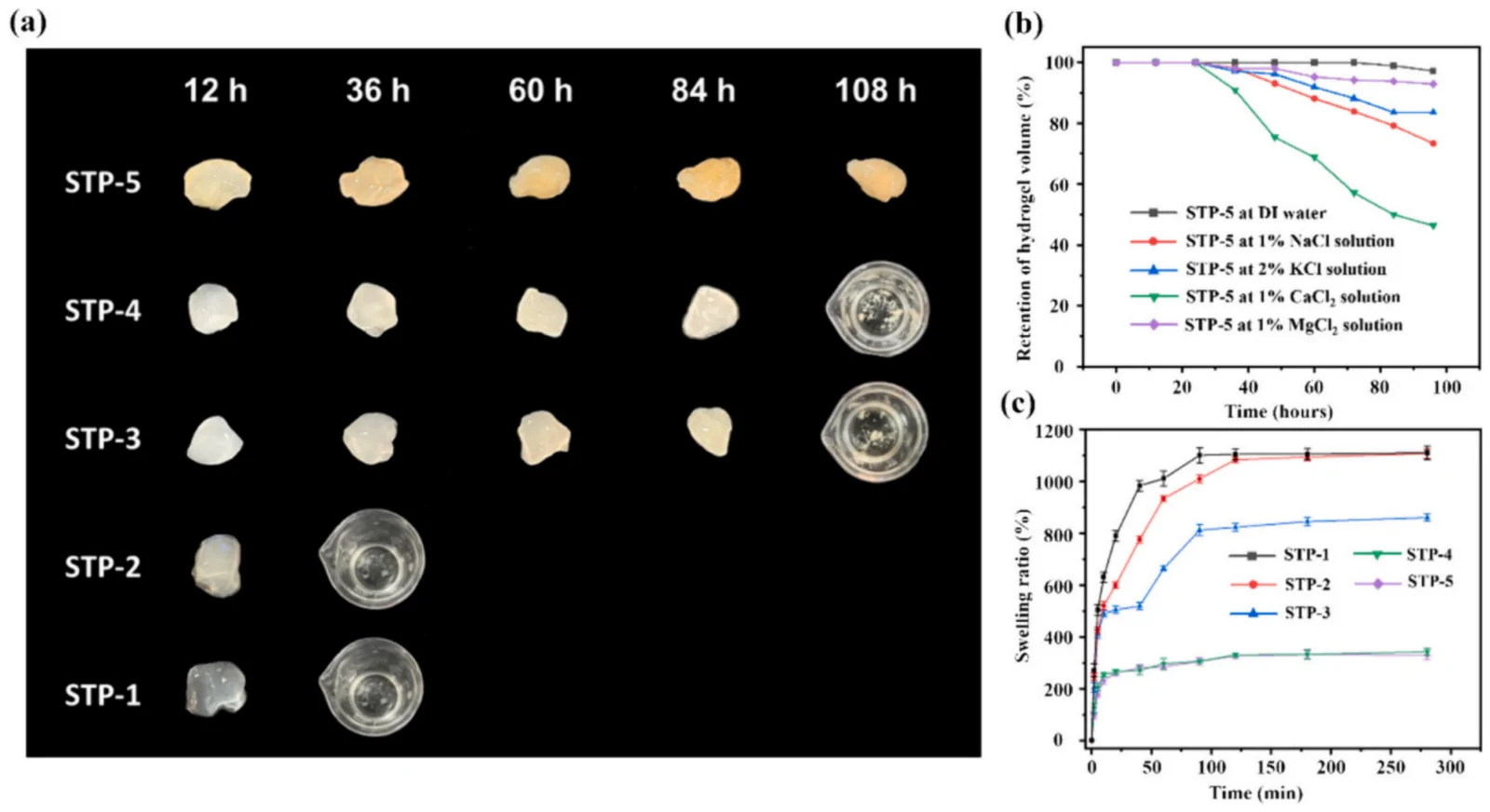
(**a**) High-temperature resistance of STP. (**b**) Hydrolytic thermal stability of hydrogel with respect to time in different solutions (namely, 1 wt% NaCl, 2 wt% KCI, 1 wt% (CaCl2), and 1 wt% (MgCl2) at 140 °C. (**c**) Swelling ratio of hydrogels in a mineralized environment at 23 °C [7] (Reproduced with permission from Jian Li, Guang Zhao, Nan Sun, et al. *Journal of Molecular Liquids*; published by Elsevier, 2022).

**Figure 35:**
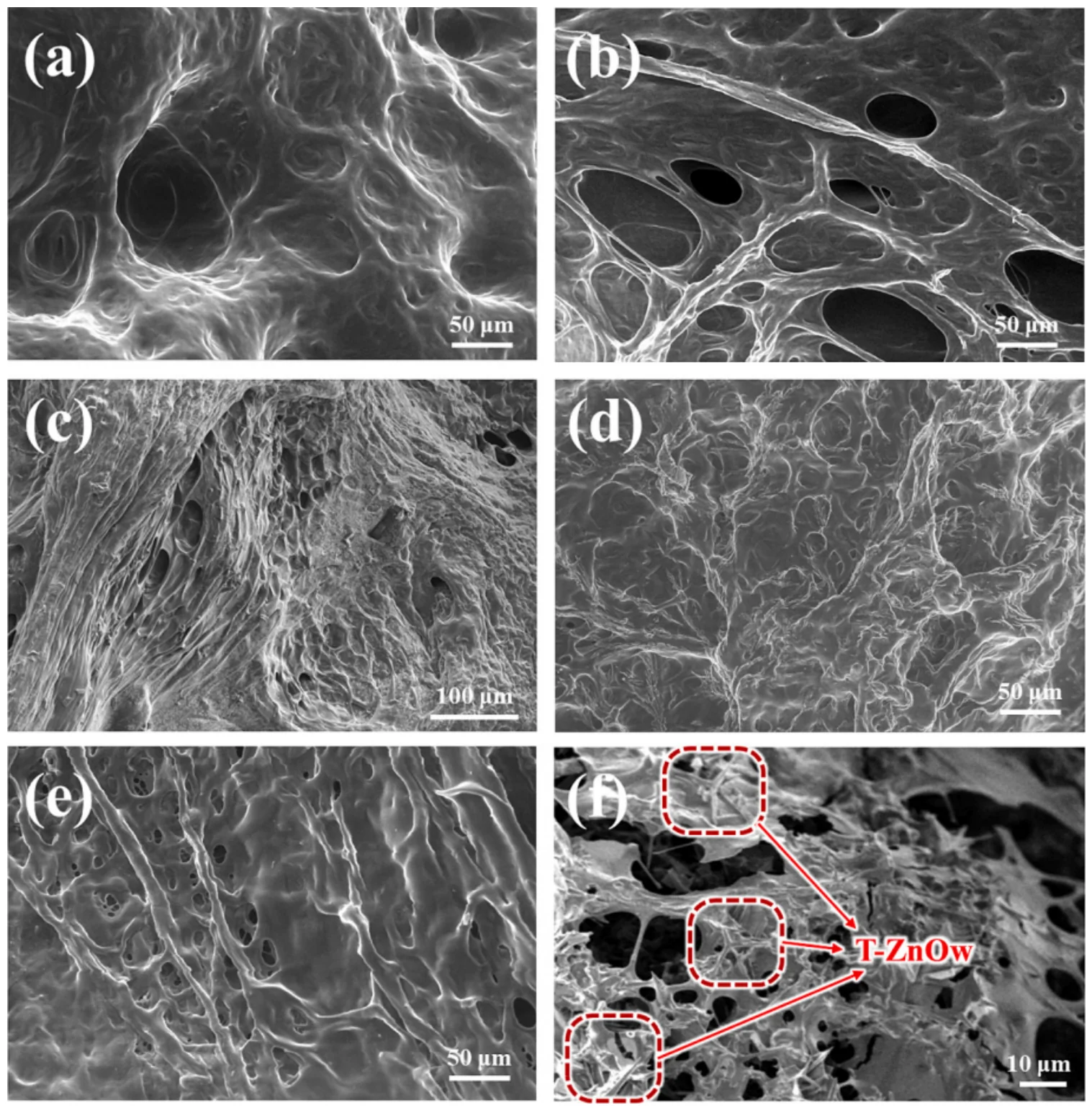
Microscopic morphology. (**a**) Gel without T-ZnOw. (**b**) Gel without T-ZnOw was aged at 130 °C for 60 days. (**c**,**d**) Gel with 0.10 wt% T-ZnOw. (**e**) Gel with 0.10 wt% T-ZnOw was aged at 130 °C for 60 days. (**f**) T-ZnOw in the gel [100] (Reproduced with permission from Chang Zou, Yang Liu, Ning Yang, et al. *Fuel*; published by Elsevier, 2024).

**Table 2:** Gelation time of HPAM/PEI gel systems at different pH levels.

Gel Systems	pH	Gelation Time (Day)
1.0 wt.% HPAM + 0.4 wt.% PEI	4.2	<1 h
	6.3	17
	8.0	5
0.4 wt.% HPAM + 0.16 wt.% PEI	4.1	<1 h
	6.3	/
	8.4	8

Data from *Study on a Polymer Gel System for Deep Profile Control in High-Temperature and High-Salinity Reservoirs. Processes*; Hui Guo, Chao Xue, Dong Wang; MDPI; 2026.

**Table 5:** Environmental-safety performance comparison of typical PAM crosslinkers.

Crosslinker	Toxicity (Acute & Chronic)	Biodegradability	Environmental Persistence	Corrosivity
PEI	Low acute toxicity, but branched high-MW PEI causes skin irritation and chronic aquatic hazards; residual PEI in produced water impairs aquatic organisms [80].	Poor biodegradability; branched high-MW PEI is barely degraded in situ, and only partial chain scission occurs under strong oxidation [67].	Moderate persistence: gel-bound PEI is immobilized, while free residual PEI persists for weeks-months in produced water [42].	Mild-moderate corrosivity; alkaline PEI accelerates Al-alloy downhole-tool corrosion, with low attack on carbon steel at neutral pH [44,67].
Cr^3+^	Low acute toxicity vs. Cr^6+^; retains chronic aquatic toxicity; aerobic conditions risk partial oxidation to toxic Cr^6+^.	Non-biodegradable metal ion; Cr^3+^ may desorb in high-salinity brine via migration or precipitation [81].	High persistence. Non-degradable Cr^3+^ can remain adsorbed within rock pores for years [81].	Low intrinsic corrosivity; oxygen-rich brine with dissolved Cr^3+^ promotes pipeline pitting corrosion [81].
Phenol-formaldehyde crosslinker	High hazard. Unreacted phenol-formaldehyde monomers are irritants and suspected carcinogens; fully cross-linked matrix shows lowered toxicity [42].	Poor biodegradability; cross-linked resin backbone is recalcitrant; only free small monomers undergo partial microbial degradation [42].	High persistence. Cross-linked resin resists hydrolysis and biodegradation; resin fragments persist underground [42].	Moderate-high corrosivity. Acidic HMTA hydrolysates aggravate pipeline corrosion; phenol degrades rubber seals [42].
Nano-composite-modified crosslinker	Toxicity is filler-dependent. GO exhibits dose-dependent cytotoxicity; T-ZnOw releases toxic Zn^2+^ in acidic brine; nano-SiO_2_ has low toxicity [7,82].	Poor biodegradability; inorganic nanofillers are non-biodegradable; only the polymer matrix may experience partial hydrolysis [7,82].	High persistence. Inorganic nanoparticles are non-biodegradable; they migrate and get trapped within reservoir pore spaces [7,82].	Low-to-mild corrosivity. Inert oxide particles may trigger under-deposit pipeline corrosion after sedimentation [7,82].
Novel delayed-release/green crosslinkers	Low-to-moderate toxicity. Biomass-derived agents show low acute toxicity; some synthetic retarders remain irritants [8,83].	Variable biodegradability: biomass-based agents degrade readily; synthetic delayed-release crosslinkers degrade poorly [8,83].	Low-to-moderate persistence. Hydrolyzable bonds enable partial in situ degradation; toxicology of degradation by-products remains unvalidated [8,83].	Mild-to-low corrosivity; neutral-to-weakly-alkaline formulations induce minimal corrosion to carbon-steel tubulars [8,83].

**Table 7:** Comparison of core performance between conventional gel and 0.2 wt% MG hybrid gel.

Performance Indicator	Conventional Gel (Without MG)	0.2 wt%MG Hybrid Gel	Improvement Effect
Gelation Time (140 °C)	8 h	4 h	Reduced by 50%
Storage Modulus (G′)	-	Increased by more than 2 times	-
Maximum Tolerable Temperature (DSC Test)	137.2 °C	154.9 °C	Increased by 17.7 °C
Bound Water Content	4.11%	21.24%	Increased by 5.1 times

Data from *Preparation and enhancement mechanisms of a novel modified nanographite hybrid polymer gel for profile control in deep reservoirs. Colloids and Surfaces A: Physicochemical and Engineering Aspects*; Shuai Lu, Qiang Liu, Peng Li, et al.; Elsevier; 2024.

**Table 8:** Key features and performance enhancement of nano-modifiers for PAM-based gels.

Nanophase Materials	Structural Characteristics and Reinforcement Mechanisms	Key Performance Improvements (Representative Data)	Representative Literature
Nano-SiO_2_	Amorphous silica particles with a particle size of 1–100 nm and a surface rich in reactive silanol groups; these particles form ionic and hydrogen bonds with the PAM molecular chains, thereby providing physical reinforcement, auxiliary crosslinker, and microstructure modulation.	Adding 1% SiO_2_ reduces the gelation time at 80 °C to 8 h (at 150 °C to 6 h); a 0.3 wt% SiO_2_ addition doubles the surface viscosity by more than twofold, increases the bound water content from 22.5% to 39.9%, and raises the maximum service temperature from 137.8 °C to 155.5 °C; the addition of 0.4% HCSIL200 increases the elastic modulus from 36.1 Pa to 57.3 Pa (an increase of nearly twofold).	[87,88,89,90,91,93,94]
Modified nano-graphene (MG)	Two-dimensional nanosheets are grafted with –SO_3_H and –OH groups via a diazotization reaction; the hydroxyl groups then undergo chemical crosslinking with the –CONH_2_ groups of PAM to form a dense three-dimensional network, while the sulfonic acid groups convert free water into bound water through hydrogen bonding.	0.2 wt% MG reduced the gelation time from 8 h to 4 h (a 50% reduction), increased the G′ value by more than twofold, raised the maximum allowable temperature from 137.2 °C to 154.9 °C (+17.7 °C), and increased the bound water content from 4.11% to 21.24% (an increase of 5.1-fold); after 60 days of aging at 140 °C, the modulus showed no significant degradation.	[43,46,95,97,99]
TEMPO-oxidized cellulose nanofibers (TOCN)	Plant-based fibers possess a surface rich in –OH groups, a high length-to-diameter ratio, and biodegradability; their properties are synergistically enhanced through C–O–C covalent crosslinking, physical skeleton support, and hydrogen bonding.	Swelling ratio: approximately 1100% → 280%; structural integrity maintained in a 140 °C saline solution for 108 h; ultimate strain tolerance reaches 1000%; compressive strength and G′ are significantly enhanced.	[7,43]
Four zinc oxide whiskers (T-ZnOw)	Four needle-like three-dimensional structures, with needle lengths ranging from 5–30 μm and root diameters ranging from 0.5–5 μm; characterized by low density and high specific surface area; enhanced by a three-dimensional “rivet-type” multi-point physical anchoring network, offering superior stability compared to spherical or sheet-like nanomaterials.	0.15 wt% T-ZnOw subjected to 60-day aging at 130 °C: elastic modulus retention rate: 44.6% → 76.5% (maximum elastic modulus: 6.2 → 21.8 Pa); strength grade: E → F; peak inflection temperature: 126.72 → 156.67 °C; no significant dehydration observed.	[100]

## Data Availability

The data are openly available in the article.

## Abbreviations

The following abbreviations are used in this manuscript:

mD	millidarcy
*G*′	storage modulus
*G*″	loss modulus
PAM	polyacrylamide
WOS	web of science
PEI	polyethyleneimine
HQ	hydroquinone
HMTA	hexamethylenetetramine
HPAM	hydrolyzed polyacrylamide
PPG	pre-crosslinked particulate gel
Slg	scleroglucan
SSS	sodium styrene sulfonate
EOR	enhanced oil recovery
AM	homo-polymerization of acrylamide
NPAM	non-ionic polyacrylamide
CPAM	cationic polyacrylamide
APAM	anionic polyacrylamide
AmPAM	amphoteric polyacrylamide
-CO-NR-	amide-type covalent bridge bonds
-CONH_2_	amide groups
-COO^−^	carboxyl groups
[Cr(H_2_O)_6_]^3+^	hydrated chromium ions
Cr^3+^	trivalent chromium ions
GO	graphene oxide
CTAB	cetyltrimethylammonium bromide
SiO_2_	silica
C- SiO_2_	CTAB- SiO_2_
nano-SiO_2_	nano-silicon dioxide
THMBPA	Water-soluble hydroxymethyl polyphenols
DSC	differential scanning calorimetry
SEM	scanning electron microscope
CG	crude glycerol
-NH2, -NHR	amine groups
NH3	ammonia
-C-NH-	amide bonds
NMR	nuclear magnetic resonance
SPG	seamless polymer gel
-CH2OH	hydroxymethyl groups
-CH_2_-NH-	nitrogen-methylene bridge
PAM-PEI	PAM cross-linked with PEI
PAM-HQ-HMTA	PAM cross-linked with HQ-HMTA
STP	high-temperature-resistant composite hydrogel
TEMPO	2,2,6,6-tetramethylpiperidine-1-oxyl radical
PAAS	polyacrylic acid-tannic acids
TOCN	TEMPO-oxidized cellulose nanofibers
-COOH	Carboxyl group
PAMGO	polyacrylamide graphene oxide
SRC	sustained-release crosslinker
WTP	water-soluble thixotropic polymer
HTHS	High temperature high sanity
HTHP	High temperature high pressure
β-CCs	β-cyclodextrin-carbon dots composite crosslinker
MG	modified nano-graphite
-SO3H	raft sulfonic acid group
-OH	Hydroxide
T-ZnOW	ur-needle zinc oxide whiskers
Ea	activation energy
DOSY	diffusion ordered spectroscopy
